# Synthesis and Evaluation of New Pyrazoline Derivatives as Potential Anticancer Agents in HepG-2 Cell Line

**DOI:** 10.3390/molecules22030467

**Published:** 2017-03-16

**Authors:** Weijie Xu, Ying Pan, Hong Wang, Haiyan Li, Qing Peng, Duncan Wei, Cheng Chen, Jinhong Zheng

**Affiliations:** 1 Department of Chemistry, Shantou University Medical College, Shantou 515041, Guangdong, China; 13536920534@163.com (W.X.); ypan@stu.edu.cn (Y.P.); 13592851552@163.com (H.W.); weiduncan2012@163.com (D.W.); 13411963192@163.com (C.C.); 2 Department of Pharmacology, Shantou University Medical College, Shantou 515041, Guangdong, China; 13066360950@163.com; 3 Department of Hepatobiliary Surgery II, Zhujiang Hospital of Southern Medical University, Guangzhou 510280, Guangdong, China; zhujiangzhuanhua@163.com

**Keywords:** pyrazoline, anticancer activity, HepG-2 cells, apoptosis

## Abstract

Cancer is a major public health concern worldwide. Adverse effects of cancer treatments still compromise patients’ quality of life. To identify new potential anticancer agents, a series of novel pyrazoline derivatives were synthesized and evaluated for cytotoxic effects on HepG-2 (human liver hepatocellular carcinoma cell line) and primary hepatocytes. Compound structures were confirmed by ^1^H-NMR, mass spectrometry, and infrared imaging. An in vitro assay demonstrated that several compounds exerted cytotoxicity in the micromolar range. Benzo[*b*]thiophen-2-yl-[5-(4-hydroxy-3,5-dimethoxy-phenyl)-3-(2-hydroxy-phenyl)-4,5-dihydo-pyrazol-1-yl]-methanone (**b17**) was the most effective anticancer agent against HepG-2 cells owing to its notable inhibitory effect on HepG-2 with an IC_50_ value of 3.57 µM when compared with cisplatin (IC_50_ = 8.45 µM) and low cytotoxicity against primary hepatocytes. Cell cycle analysis and apoptosis/necrosis evaluation using this compound revealed that **b17** notably arrested HepG-2 cells in the G_2_/M phase and induced HepG-2 cells apoptosis. Our findings indicate that compound **b17** may be a promising anticancer drug candidate.

## 1. Introduction

Worldwide, liver cancer is the third most common cause of cancer deaths. It is the fifth and seventh most common cancer in men and women, respectively [1]. Cancer is a complex disease caused by various factors, such as high stress, bad dietary habits, aging, and smoking; uncontrolled, rapid, and pathological proliferation of abnormally transformed cells is a direct cause of a large group of diseases [2,3,4]. Great progress has been made in medical treatments, but cancer is still a major cause of mortality. Resistance to chemotherapeutic agents, lack of selectivity, and serious adverse effects are the primary challenges in the fight against cancer [2,3,4,5,6]. Therefore, new anticancer agents are continually developed and tested to selectively destroy tumor cells or at least limit their proliferation.

**Figure 1:**
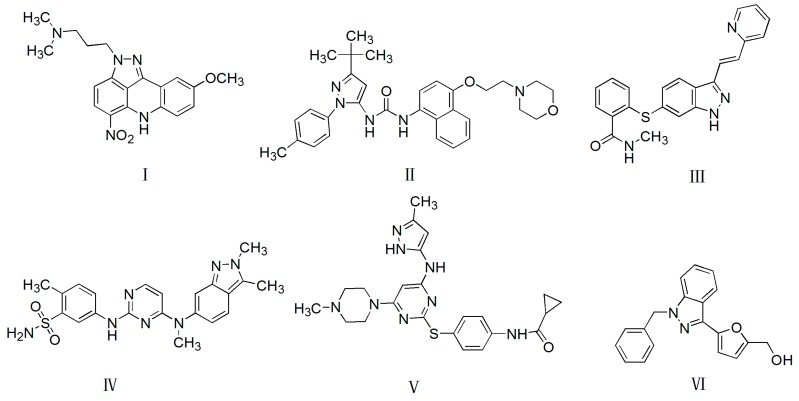
Structures of pyrazoloacridine (PZA) (**I**); doramapimod (BIRB-796) (**II**); axitinib (AG013736) (**III**); pazopanib (GW786034) (**IV**); tozasertib (VX-680) (**V**); and 3-(5’-hydroxymethyl-2’-furyl)-1-benzyl indazole (YC-1) (**VI**).

Pyrazolines are a class of compounds exhibiting a wide spectrum of activities. Several pyrazolines act as anticancer agents [7,8], tubulin assembly inhibitors [9]. Pyrazoloacridine (PZA) (I) (Figure 1) is a new anticancer drug currently undergoing Phase II clinical trials [10,11,12]. Doramapimod (BIRB-796) (II) is a selective p38α mitogen-activated protein kinase (MAPK) inhibitor undergoing Phase III clinical trials [13,14]. Axitinib (AG013736) (III), a vascular endothelial growth factor receptor (VEGFR) inhibitor, used in clinical treatment, is exploited by Pfizer [15,16]. Pazopanib (GW786034) (IV), a VEGFR inhibitor, is exploited by GlaxoSmithKline [17,18]. Tozasertib (VX-680, MK-0457) (V) is an Aurora kinase inhibitor [19,20] and 3-(5′-hydroxymethyl-2’-furyl)-1-benzyl indazole (YC-1) (VI) is a hypoxia-inducible factor (HIF)-1 inhibitor as well as a VEGF inhibitor [21,22]. They are promising anticancer drug candidates. Recent studies report that a large number of sulfonamide derivatives and heterocyclic compounds show antitumor activity and display a common chemical motif of aromatic/heterocyclic sulfonamide. The compounds’ antitumor action is attributed to varying mechanisms, such as cell cycle perturbation, disruption of microtubule assembly, and functional suppression of the transcriptional activator nuclear factor (NF)-Y (a protein that binds to DNA and regulates gene expression by promoting or suppressing transcription) [23,24,25,26]. In this report, we describe the synthesis of new pyrazoline derivatives (**b1**–**19**) with substituted sulfonyl moieties, heterocyclic rings, or other closely related variants in their molecular structures. We show the synthesis and evaluation of a new class of pyrazolines acting as potential antitumor agents against a human liver hepatocellular carcinoma cell line (HepG-2) and primary hepatocytes. We also carried out an analysis of the cell cycle, apoptosis, and necrosis processes using the most effective compound, **b17**.

## 2. Results and Discussion

### 2.1. Synthesis of New Pyrazoline Derivatives

**Scheme 1:**
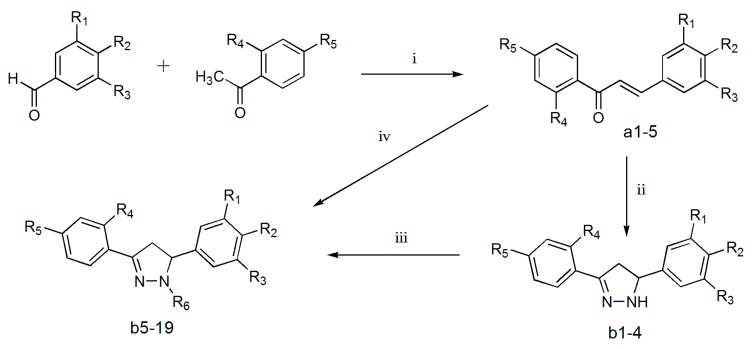
General synthesis of compounds **b1**–**19**. *Reagents and conditions*: (i) substituted phenylethanone (0.01 mol), substituted benzaldehyde (0.012 mol), piperidine (1 mL), 160 °C, 20 min or 30% NaOH, ethanol, r.t., 24 h; (ii) ethanol, hydrazine hydrate, reflux, 4 h; (iii) substituted benzoyl chloride or substituted benzenesulfonyl chloride, 80 °C, ethanol, pyridine, 1 h; (iv) ethanol, phenylhydrazine, tetrabutylammonium bromide (TBAB), reflux, 1 h.

The synthesis of new pyrazoline derivatives (**b1**–**19**) was carried out according to the steps shown in Scheme 1. First, chalcone derivatives (**a1**–**5**) were obtained via the base-catalyzed Claisen-Schmidt condensation with corresponding ketones and aldehydes. Chalcone ring-closure reactions were carried out with hydrazine or phenylhydrazine with tetrabutylammonium bromide (TBAB) as a catalyst to obtain compounds **b1**–**4**, **b18**, and **b19**. Next, compound **b1** was treated with corresponding acyl chlorides or sulfonyl chlorides at 80 °C with ethanol as a solvent and pyridine as a catalyst to yield compounds **b5**–**17**. Compounds **b1**–**19** were elucidated by infrared (IR), ^1^H-NMR, and mass spectrometry imaging. Thus, the synthetic procedure was shown to be versatile and applicable to the preparation of many derivatives. Compounds **a1**–**5** are shown in Table 1; compounds **b1**–**19** are shown in Table 2.

### 2.2. MTT Assay

**Figure 2:**
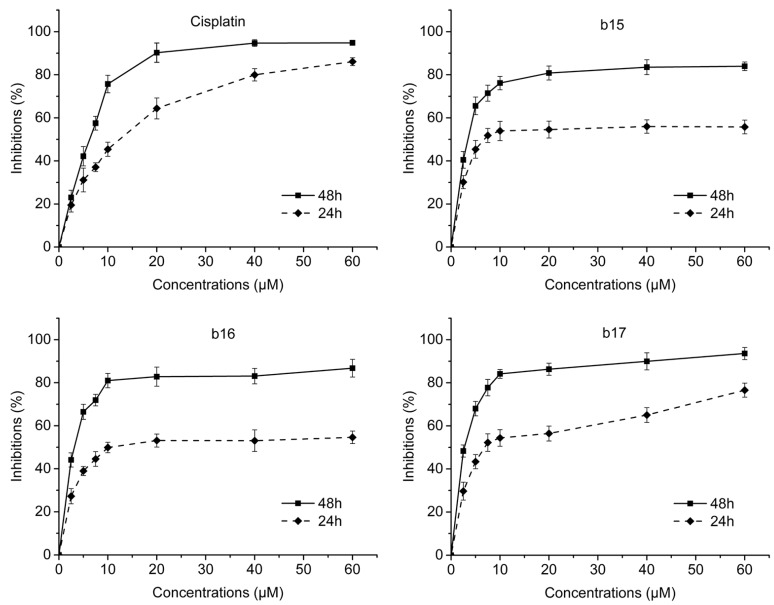
Inhibitory effects of compounds **b15**–**17** and cisplatin on HepG-2 cells after 24 h and 48 h.

To identify the most promising antitumor agent among the synthesized pyrazoline derivatives (**b1**–**19**), their cytotoxic effects were tested on HepG-2 cells; cisplatin was used as a positive control. We found that compounds **b5**, **b9**, and **b14**–**18** displayed IC_50_ values lower than 50 µM against HepG-2 cells at 48 h. The most effective cytotoxic agent was compound **b17**, with an IC_50_ value of 3.57 µM at 48 h; cisplatin, the positive control, had an IC_50_ value of 8.45 µM at 48 h (Table 3). The pyrazoline derivatives also displayed dose-dependent and time-dependent trends. We selected the three most effective compounds, **b15**–**17**, along with cisplatin to generate growth-inhibitory curves (Figure 2).

### 2.3. MTS Assay

According to the results of MTT (thiazolyl blue tetrazolium bromide) assay on HepG-2 cell line, the cytotoxicity of compounds **b5**, **b9**, and **b14**–**18** were evaluated against primary hepatocytes using the MTS assay. (Isolated primary hepatocytes were unstable. Using the MTS assay, the solubilization steps were eliminated because the MTS formazan product is soluble in tissue culture medium. It can avoid further damage to cells.) Compounds **b5**, **b9**, and **b14**–**18** showed lower cytotoxicological activity against primary hepatocytes. The IC_50_ value of compound **b17** against primary hepatocytes was 33.47 µM at 48 h. In terms of their anticancer potential, compound **b17** can be considered as the most promising anticancer agent against HepG-2 due to its low cytotoxicity against primary hepatocytes (Table 3).

### 2.4. Cell Cycle Analysis

**Figure 3:**
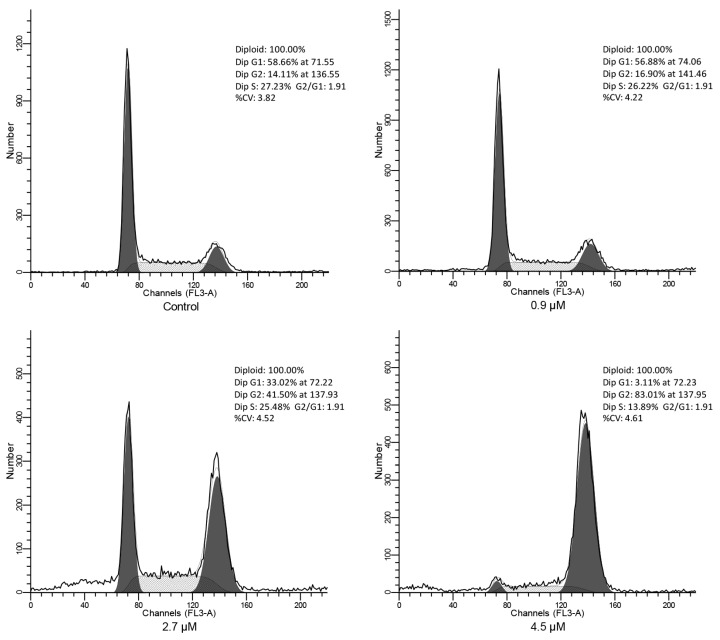
Effects of compound **b17** on cell cycle progression in HepG-2 cells. Cells were treated with **b17** at concentrations of 0 µM, 0.9 µM, 2.7 µM, and 4.5 µM for 24 h.

To determine whether compound **b17** would affect the cell cycle in HepG-2 cells, we examined cell cycle progression using flow cytometry (Figure 3). HepG-2 cells were treated with compound **b17** at concentrations of 0.9 µM, 2.7 µM, and 4.5 µM for 24 h. A notable decrease in cells in the G_1_ and S phases was observed. At 4.5 µM, up to 83.01% of the cells were arrested in the G_2_/M phase (the data shown in Figure 3 is about living cells after exclusion of dead cells). These findings indicate that compound **b17** may be a potent anticancer agent.

### 2.5. Annexin-V Assay

**Figure 4:**
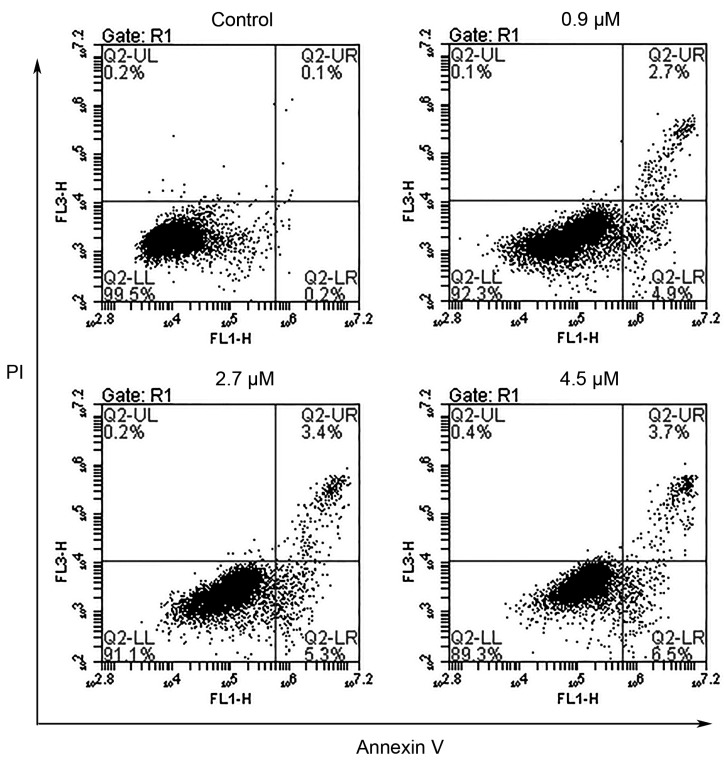
Compound **b17** induced apoptosis in HepG-2 cells at concentrations of control, 0.9 µM, 2.7 µM, and 4.5 µM in a 12 h exposure. PI: propidium iodide.

A biparametric cytofluorimetric analysis was performed to determine the mode of cell death induced by compound **b17** using propidium iodide (PI) and fluorescent immunolabeling of the protein annexin V. HepG-2 cells were treated with compound **b17** at concentrations of 0.9 µM, 2.7 µM, and 4.5 µM for 12 h. The percentage of cell apoptosis was 0.3% at 0 µM for compound **b17**, and 7.6%, 8.7%, and 10.2% at 0.9 µM, 2.7 µM, and 4.5 µM, respectively (Figure 4). Thus, we conclude that compound **b17** can induce apoptosis.

## 3. Experimental Section

### 3.1. Chemical Reagents and Equipment

All reagents were purchased from commercial suppliers. Melting points were determined using an Electrothermal 9100 melting point apparatus (Weiss-Gallenkamp, Loughborough, UK). IR spectra were recorded on a Bruker Tensor 27 Fourier IR spectrometer (Bruker, Karlsruhe, Germany). ^1^H-NMR spectra were recorded on a Bruker Avance 300 spectrometer (Bruker) using tetramethylsilane (TMS) as the internal standard. Mass spectra were recorded on a SCIEX Triple Quad 6500+ LC/MS/MS system (SCIEX, Los Angeles, CA, USA). HRMS (high-resolution mass spectrometry) was performed on a Thermo Scientific Q Exactive (Thermo, Waltham, MA, USA).

### 3.2. General Procedures for the Synthesis of Compounds **a1**–**5**

Compounds **a1**–**4** were obtained by a mixture of substituted phenylethanone (0.01 mol), substituted benzaldehyde (0.012 mol), and piperidine (1 mL) as a catalyst, and stirred at 160 °C for 20 min. Next, 30% aqueous sodium hydroxide solution (30 mL) was added and stirred for 15 min [27]. Compound **a5** was prepared by appropriate substituted phenylethanone (0.01 mol), substituted benzaldehyde (0.012 mol), and 30% aqueous sodium hydroxide solution (10 mL) in ethanol (30 mL), and stirred at 30 °C for 24 h. The progress of the reaction was checked by thin-layer chromatography (TLC). Upon completion, the reaction mixture was poured onto crushed ice, followed by neutralization with HCl [28]. The precipitated solid was filtered, washed with water, and dried by vacuum pump. The product was finally crystallized from ethanol.

### 3.3. General Procedures for the Synthesis of Compounds **b1**–**19**

Hydrazine hydrate (0.04 mol, 1.96 g) was added to a solution of compound **a1** (0.01 mol, 3.00 g) in ethanol (10 mL). The mixture was refluxed under stirring for 4 h, and the reaction mixture was subsequently poured onto crushed ice; the precipitate was filtered out, and product **b1** was later crystallized from ethanol. Compounds **b2**–**4** were synthesized following this procedure. Compounds **b5**–**17** were prepared by treating compound **b1** (0.01 mol, 3.14 g) with corresponding substituted benzoyl chloride or substituted benzenesulfonyl chloride (0.015 mol) at 80 °C in ethanol as solvent with pyridine as catalyst for 1 h to yield the final *N*-substituted targeted compounds. The *N*-phenyl-substituted pyrazolines **b18** and **b19** were prepared by direct cyclization of **a1** and **a5**, respectively, with phenylhydrazine in the presence of TBAB as a catalyst [29].

*4-[5-(2-Hydroxyphenyl)-3,4-dihydro-2H-pyrazol-3-yl]-2,6-dimethoxy-phenol* (**b1**). Yield: 70.62%; M.p. 140.3–140.7 °C. IR (KBr) ν_max_ (cm^−1^): 3277 (aromatic –OH), 2994, 2946 (aliphatic C–H asymmetric), 1618, 1498, 1461, 1424 (C=N and C=C), 1352, 1258, 1210, 1130 (C–N). ^1^H-NMR (400 MHz, δ ppm, DMSO-*d*_6_): 2.96–3.03 (1H, m, pyrazoline C_4_–H_A_), 3.54–3.58 (1H, m, pyrazoline C_4_–H_B_), 3.76 (6H, s, –OCH_3_), 4.74–4.81 (1H, m, pyrazoline C_5_–H*_x_*), 6.70 (2H, s), 6.88–6.93 (2H, m), 7.24 (1H, t, *J* = 8.00 Hz), 7.31 (1H, d, *J* = 8.00 Hz), 7.78 (1H, s, –NH–), 8.31 (1H, s, –OH), 11.21 (1H, s, –OH). MS (ESI) (*m*/*z*): 315 [M + H]^+^.

*2,6-Dimethoxy-4-[5-(4-nitrophenyl)-3,4-dihydro-2H-pyrazol-3-yl]-phenol* (**b2**). Yield: 71.22%; M.p. 140.7–141.1 °C. IR (KBr) ν_max_ (cm^−1^): 3319 (aromatic –OH), 2971, 2844 (aliphatic C–H asymmetric), 1614, 1464, 1430, 1407 (C=N and C=C), 1340, 1223, 1166, 1117 (C–N). ^1^H-NMR (400 MHz, δ ppm, DMSO-*d*_6_): 2.89–2.96 (1H, m, pyrazoline C_4_–H_A_), 3.43–3.50 (1H, m, pyrazoline C_4_–H_B_), 3.74 (6H, s, –OCH_3_), 4.85–4.91 (1H, m, pyrazoline C_5_–H*_x_*), 6.65 (2H, s), 7.82 (2H, d, *J* = 8.00 Hz ), 8.19 (1H, s, –NH–), 8.22 (2H, d, *J* = 8.00 Hz), 8.32 (1H, s, –OH). MS (ESI) (*m*/*z*): 344 [M + H]^+^.

*2-Bromo-4-[5-(2-hydroxyphenyl)-3,4-dihydro-2H-pyrazol-3-yl]-6-methoxy-phenol* (**b3**). Yield: 70.01%; M.p. 181.8–183.9 °C. IR (KBr) ν_max_ (cm^−1^): 3314 (aromatic –OH), 2966, 2938 (aliphatic C–H asymmetric), 1620, 1499, 1424, 1348 (C=N and C=C), 1262, 1187, 1153, 1049 (C-N). ^1^H-NMR (400 MHz, δ ppm, DMSO-*d*_6_): 2.98–3.05 (1H, m, pyrazoline C_4_–H_A_), 3.55–3.61 (1H, m, pyrazoline C_4_-H_B_ ), 3.83 (3H, s, –OCH_3_), 4.76–4.81 (1H, m, pyrazoline C_5_–H*_x_*), 6.88–6.93 (2H, m), 7.04 (1H, s), 7.10 (1H, s), 7.22–7.26 (1H, t, *J* = 8.00 Hz), 7.30 (1H, d, *J* = 8.00 Hz), 7.81 (1H, s, –NH–), 9.42 (1H, s, –OH), 11.15 (1H, s, –OH). MS (ESI) (*m*/*z*): 363 [M + H]^+^.

*2-Bromo-6-methoxy-4-[5-(4-nitrophenyl)-3,4-dihydro-2H-pyrazol-3-yl]-phenol* (**b4**). Yield: 76.11%; M.p. 208.6–208.9 °C. IR (KBr) ν_max_ (cm^−1^): 3401, 3325 (aromatic –OH), 2965, 2934 (aliphatic C–H asymmetric), 1614, 1466, 1451, 1416 (C=N and C=C), 1333, 1274, 1187, 1049 (C-N). ^1^H-NMR (400 MHz, δ ppm, DMSO-*d*_6_): 2.91–2.98 (1H, m, pyrazoline C_4_–H_A_), 3.44–3.51 (1H, m, pyrazoline C_4_–H_B_), 3.82 (3H, s, –OCH_3_), 4.87–4.93 (1H, m, pyrazoline C_5_–H*_x_*), 6.99 (1H, s), 7.06 (1H, s), 7.82 (2H, d, *J* = 8.00 Hz), 8.22 (2H, d, *J* = 8.00 Hz), 8.24 (1H, s, –NH–), 9.39 (1H, s, –OH). MS (ESI) (*m*/*z*): 392 [M + H]^+^.

*4-[2-Benzenesulfonyl-5-(2-hydroxyphenyl)-3,4-dihydro-2H-pyrazol-3-yl]-2,6-dimethoxy-phenol* (**b5**). Yield: 72.44%; M.p. 163.1–164.4 °C. IR (KBr) ν_max_ (cm^−1^): 3447, 3194 (aromatic –OH), 2969, 2937 (aliphatic C–H asymmetric), 1619, 1461, 1355, 1219 (C=N and C=C), 1171, 1116, 1056, 1002 (C–N). ^1^H-NMR (400 MHz, δ ppm, DMSO-*d*_6_): 3.27–3.32 (1H, m, pyrazoline C_4_–H_A_), 3.67–3.72 (1H, m, pyrazoline C_4_–H_B_), 3.77 (6H, s, –OCH_3_), 4.84–4.89 (1H, m, pyrazoline C_5_–H*_x_*), 6.70 (2H, s), 6.90 (1H, t, *J* = 8.00 Hz), 6.96 (1H, d, *J* = 8.00 Hz), 7.35 (1H, t, *J* = 8.00 Hz), 7.44 (1H, d, *J* = 8.00 Hz), 7.66 (1H, t, *J* = 8.00 Hz), 7.74 (2H, t, *J* = 8.00 Hz), 7.85 (2H, d, *J* = 8.00 Hz), 8.42 (1H, s, –OH), 10.31 (1H, s, –OH). MS (ESI) (*m*/*z*): 455 [M + H]^+^.

*4-[5-(2-Hydroxyphenyl)-2-(2-nitro-benzenesulfonyl)-3,4-dihydro-2H-pyrazol-3-yl]-2,6-dimethoxy-phenol* (**b6**). Yield: 40.03%; M.p. 151.1–152.8 °C. IR (KBr) ν_max_ (cm^−1^): 3441, 3211 (aromatic –OH), 2930, 2837 (aliphatic C–H asymmetric), 1619, 1462, 1431, 1376 (C=N and C=C), 1304, 1180, 1113, 1008 (C–N). ^1^H-NMR (400 MHz, δ ppm, DMSO-*d*_6_): 3.42–3.44 (1H, m, pyrazoline C_4_–H_A_), 3.76 (6H, s, –OCH_3_), 3.91–3.98 (1H, m, pyrazoline C_4_–H_B_), 5.19–5.24 (1H, m, pyrazoline C_5_–H*_x_*), 6.67 (2H, s), 6.91–6.98 (2H, m), 7.36 (1H, t, *J* = 8.00 Hz), 7.55 (1H, d, *J* = 8.00 Hz), 7.84 (1H, t, *J* = 8.00 Hz), 7.93 (1H, t, *J* = 8.00 Hz), 7.99–8.03 (2H, m), 8.45 (1H, s, –OH), 10.16 (1H, s, –OH). MS (ESI) (*m*/*z*): 500 [M + H]^+^.

*4-[5-(2-Hydroxyphenyl)-2-(3-nitro-benzenesulfonyl)-3,4-dihydro-2H-pyrazol-3-yl]-2,6-dimethoxy-phenol* (**b7**). Yield: 51.37%; M.p. 131.9–133.5 °C. IR (KBr) ν_max_ (cm^−1^): 3466, 3198 (aromatic –OH), 2971, 2938 (aliphatic C–H asymmetric), 1619, 1493, 1463, 1428 (C=N and C=C), 1357, 1216, 1177, 1116 (C–N). ^1^H-NMR (400 MHz, δ ppm, DMSO-*d*_6_): 3.43–3.46 (1H, m, pyrazoline C_4_–H_A_), 3.72 (6H, s, –OCH_3_), 3.75–3.78 (1H, m, pyrazoline C_4_–H_B_), 4.95–4.99 (1H, m, pyrazoline C_5_–H*_x_*), 6.61 (2H, s), 6.91 (1H, t, *J* = 8.00 Hz), 6.96 (1H, d, *J* = 8.00 Hz), 7.35 (1H, d, *J* = 8.00 Hz), 7.52 (1H, d, *J* = 8.00 Hz), 7.92 (1H, t, *J* = 8.00 Hz), 8.21 (1H, d, *J* = 8.00 Hz), 8.34 (1H, s), 8.45 (1H, s, –OH), 8.51 (1H, d, *J* = 8.00 Hz), 10.24 (1H, s, –OH). MS (ESI) (*m*/*z*): 500 [M + H]^+^.

*4-[5-(2-Hydroxyphenyl)-2-(4-nitro-benzenesulfonyl)-3,4-dihydro-2H-pyrazol-3-yl]-2,6-dimethoxy-phenol* (**b8**). Yield: 63.51%; M.p. 195.5–197.0 °C. IR (KBr) ν_max_ (cm^−1^): 3385 (aromatic –OH), 2969, 2846 (aliphatic C–H asymmetric), 1620, 1464, 1430, 1368 (C=N and C=C), 1314, 1207, 1065, 1012 (C–N). ^1^H-NMR (400 MHz, δ ppm, DMSO-*d*_6_): 3.41–3.47 (1H, m, pyrazoline C_4_–H_A_), 3.73 (6H, s, –OCH_3_), 3.74–3.79 (1H, m, pyrazoline C4–H_B_), 4.90–4.95 (1H, m, pyrazoline C_5_–H*_x_*), 6.63 (2H, s), 6.90 (1H,t, *J* = 8.00 Hz), 6.95 (1H, d, *J* = 8.00 Hz), 7.34 (1H, t, *J* = 8.00 Hz), 7.51 (1H, d, *J* = 8.00 Hz), 8.04 (2H, d, *J* = 8.00 Hz), 8.41 (1H, d, *J* = 8.00 Hz), 8.45 (1H, s, –OH), 10.22 (1H, s, –OH). MS (ESI) (*m*/*z*): 500 [M + H]^+^.

*4-[5-(2-Hydroxyphenyl)-2-(toluene-4-sulfonyl)-3,4-dihydro-2H-pyrazol-3-yl]-2,6-dimethoxy-phenol* (**b9**). Yield: 71.43%; M.p. 194.6–194.7 °C. IR (KBr) ν_max_ (cm^−1^): 3453, 3192 (aromatic –OH), 2969, 2938 (aliphatic C–H asymmetric), 1615, 1493, 1462, 1429 (C=N and C=C), 1358, 1218, 1115, 1026 (C–N). ^1^H-NMR (400 MHz, δ ppm, DMSO-*d*_6_): 2.38 (3H, s, –CH_3_), 3.41–3.44 (1H, m, pyrazoline C_4_–H_A_), 3.67–3.75 (1H, m, pyrazoline C_4_–H_B_), 3.76 (6H, s, –OCH_3_), 4.77–4.83 (1H, m, pyrazoline C_5_–H*_x_*), 6.69 (2H, s), 6.90 (1H, t, *J* = 8.00 Hz), 6.96 (1H, d, *J* = 8.00 Hz), 7.34 (1H, t, *J* = 8.00 Hz), 7.42–7.46 (3H, m), 7.72 (2H, d, *J* = 8.00 Hz), 8.41 (1H, s, –OH), 10.33 (1H, s, –OH). MS (ESI) (*m*/*z*): 469 [M + H]^+^.

*[5-(4-Hydroxy-3,5-dimethoxyphenyl)-3-(2-hydroxyphenyl)-4,5-dihydro-pyrazol-1-yl]-(2-nitrophenyl)-methanone* (**b10**). Yield: 71.43%; M.p. 194.6–194.7 °C. IR (KBr) ν_max_ (cm^−1^): 3532, 3190 (aromatic –OH), 2936, 2836 (aliphatic C–H asymmetric), 1666 (C=O), 1618, 1524, 1441, 1342 (C=N and C=C), 1253, 1224, 1146, 1106 (C–N). ^1^H-NMR (400 MHz, δ ppm, DMSO-*d*_6_): 3.32–3.34 (1H, m, pyrazoline C_4_–H_A_), 3.78 (6H, s, –OCH_3_), 3.96–4.06 (1H, m, pyrazoline C_4_–H_B_), 5.56–5.60 (1H, m, pyrazoline C_5_–H*_x_*), 6.62 (2H, s), 6.84–6.88 (2H, m), 7.29 (1H, t, *J* = 8.00 Hz), 7.38 (1H, d, *J* = 8.00 Hz), 7.70 (1H, d, *J* = 8.00 Hz), 7.77 (1H, t, *J* = 8.00 Hz), 7.90 (1H, t, *J* = 8.00 Hz), 8.20 (1H, s), 8.37 (1H, s, –OH), 9.79 (1H, s, –OH). MS (ESI) (*m*/*z*): 464 [M + H]^+^.

*[5-(4-Hydroxy-3,5-dimethoxyphenyl)-3-(2-hydroxyphenyl)-4,5-dihydro-pyrazol-1-yl]-(3-nitrophenyl)-methanone* (**b11**). Yield: 70.03%; M.p. 207.7–209.3 °C. IR (KBr) ν_max_ (cm^−1^): 3212, 3078 (aromatic –OH), 2930, 2840 (aliphatic C–H asymmetric), 1667 (C=O), 1610, 1525, 1425, 1341 (C=N and C=C), 1310, 1214, 1159, 1113 (C–N). ^1^H-NMR (400 MHz, δ ppm, DMSO-*d*_6_): 3.35–3.37 (1H, m, pyrazoline C_4_–H_A_), 3.75 (6H, s, –OCH_3_), 3.97–4.05 (1H, m, pyrazoline C_4_–H_B_), 5.63–5.67 (1H, m, pyrazoline C_5_–H*_x_*), 6.63 (2H, s), 6.90–6.95 (2H, m), 7.33 (1H, t, *J* = 8.00 Hz), 7.56 (1H, d, *J* = 8.00 Hz), 7.82 (1H, t, *J* = 8.00 Hz), 8.28–8.30 (1H, m), 8.40–8.42 (2H, m), 8.62 (1H, s), 10.03 (1H, s, –OH). MS (ESI) (*m*/*z*): 464 [M + H]^+^.

*[5-(4-Hydroxy-3,5-dimethoxyphenyl)-3-(2-hydroxyphenyl)-4,5-dihydro-pyrazol-1-yl]-(4-nitrophenyl)-methanone* (**b12**). Yield: 68.02%; M.p. 250.1–251.2 °C. IR (KBr) ν_max_ (cm^−1^): 3378, 3266 (aromatic –OH), 2936, 2842 (aliphatic C–H asymmetric), 1650 (C=O), 1614, 1518, 1444, 1336 (C=N and C=C), 1258, 1243, 1210, 1110 (C–N). ^1^H-NMR (400 MHz, δ ppm, DMSO-*d*_6_): 3.40–3.46 (1H, m, pyrazoline C_4_–H_A_), 3.76 (6H, s, –OCH_3_), 3.98–4.05 (1H, m, pyrazoline C_4_–H_B_), 5.65–5.66 (1H, m, pyrazoline C_5_–H*_x_*), 6.62 (2H, s), 6.90–6.94 (2H, m), 7.34 (1H, t, *J* = 8.00 Hz), 7.54 (1H, d, *J* = 8.00 Hz), 8.05 (2H, d, *J* = 8.00 Hz), 8.36 (2H, d, *J* = 8.00 Hz), 8.40 (1H, s, –OH), 10.03 (1H, s, –OH). MS (ESI) (*m*/*z*): 464 [M + H]^+^.

*(3,5-Dinitrophenyl)-[5-(4-hydroxy-3,5-dimethoxyphenyl)-3-(2-hydroxyphenyl)-4,5-dihydro-pyrazol-1-yl]-methanone* (**b13**). Yield: 51.38%; M.p. 240.4–240.9 °C. IR (KBr) ν_max_ (cm^−1^): 3336, 3109 (aromatic –OH), 2939, 2843 (aliphatic C–H asymmetric), 1647 (C=O), 1614, 1543, 1465, 1432 (C=N and C=C), 1341, 1219, 1112, 1031 (C–N). ^1^H-NMR (400 MHz, δ ppm, DMSO-*d*_6_): 3.41–3.43 (1H, m, pyrazoline C_4_–H_A_), 3.75 (6H, s, –OCH_3_), 3.98–4.06 (1H, m, pyrazoline C_4_–H_B_), 5.63–5.67 (1H, m, pyrazoline C_5_–H*_x_*), 6.64 (2H, s), 6.90 (1H, t, *J* = 8.00 Hz), 6.96 (1H, d, *J* = 8.00 Hz), 7.33 (1H, t, *J* = 8.00 Hz), 7.65 (1H, d, *J* = 8.00 Hz), 8.39 (1H, s, –OH), 8.97 (1H, s), 9.07 (2H, s), 10.06 (1H, s, –OH). MS (ESI) (*m*/*z*): 509 [M + H]^+^.

*[5-(4-Hydroxy-3,5-dimethoxyphenyl)-3-(2-hydroxyphenyl)-4,5-dihydro-pyrazol-1-yl]-naphthalen-2-yl-methanone* (**b14**). Yield: 63.51%; M.p. 185.5–186.5 °C. IR (KBr) ν_max_ (cm^−1^): 3421 (aromatic –OH), 2931, 2835 (aliphatic C–H asymmetric), 1635 (C=O), 1616, 1522, 1465, 1426 (C=N and C=C), 1328, 1218, 1118, 1031 (C–N). ^1^H-NMR (400 MHz, δ ppm, DMSO-*d*_6_): 3.39–3.44 (1H, m, pyrazoline C_4_–H_A_), 3.75 (6H, s, –OCH_3_), 3.98–4.05 (1H, m, pyrazoline C_4_–H_B_), 5.67–5.71 (1H, m, pyrazoline C_5_–H*_x_*), 6.65 (2H, s), 6.89–6.94 (2H, m), 7.30–7.34 (1H, m), 7.49–7.52 (1H, m), 7.60–7.67 (2H, m), 7.86–7.88 (1H, m), 8.01–8.04 (2H, m), 8.07 (1H, d, *J* = 8.00 Hz), 8.38 (1H, s), 8.41 (1H, s, –OH), 10.13 (1H, s, –OH). MS (ESI) (*m*/*z*): 469 [M + H]^+^.

*[5-(4-Hydroxy-3,5-dimethoxyphenyl)-3-(2-hydroxyphenyl)-4,5-dihydro-pyrazol-1-yl]-thiophen-2-yl-methanone* (**b15**). Yield: 63.41%; M.p. 196.1–197.2 °C. IR (KBr) ν_max_ (cm^−1^): 3375, 3199 (aromatic –OH), 2942, 2836 (aliphatic C–H asymmetric), 1632 (C=O), 1612, 1516, 1435, 1336 (C=N and C=C), 1245, 1209, 1116, 1043 (C–N). ^1^H-NMR (400 MHz, δ ppm, DMSO-*d*_6_): 3.32–3.33 (1H, m, pyrazoline C_4_–H_A_), 3.69 (6H, s, –OCH_3_), 3.93–4.00 (1H, m, pyrazoline C_4_–H_B_), 5.57–5.61 (1H, m, pyrazoline C_5_–H*_x_*), 6.50 (2H, s), 6.94–6.99 (2H, m), 7.20–7.23 (1H, m), 7.33–7.37 (1H, m), 7.78–7.80 (1H, m), 7.92–7.93 (1H, m), 7.99–8.00 (1H, m), 8.37 (1H, s, –OH), 10.19 (1H, s, –OH). MS (ESI) (*m*/*z*): 425 [M + H]^+^.

*Furan-2-yl-[5-(4-hydroxy-3,5-dimethoxyphenyl)-3-(2-hydroxyphenyl)-4,5-dihydro-pyrazol-1-yl]-methanone* (**b16**). Yield: 63.41%; M.p. 201.1–203.7 °C. IR (KBr) ν_max_ (cm^−1^): 3355, 3128 (aromatic –OH), 2940, 2837 (aliphatic C–H asymmetric), 1620 (C=O), 1600, 1519, 1458, 1426 (C=N and C=C), 1340, 1248, 1209, 1117 (C–N). ^1^H-NMR (400 MHz, δ ppm, DMSO-*d*_6_): 3.29–3.34 (1H, m, pyrazoline C_4_–H_A_), 3.70 (6H, s, –OCH_3_), 3.88–3.97 (1H, m, pyrazoline C_4_–H_B_), 5.57–5.61 (1H, m, pyrazoline C_5_–H*_x_*), 6.51 (2H, s), 671–6.72 (1H, m), 6.93–7.00 (2H, m), 7.35 (1H, t, *J* = 8.00 Hz), 7.51–7.52 (1H, m), 7.64 (1H, d, *J* = 8.00 Hz), 7.96 (1H, s), 8.36 (1H, s, –OH), 10.47 (1H, s, –OH). MS (ESI) (*m*/*z*): 409 [M + H]^+^.

*Benzo[b]thiophen-2-yl-[5-(4-hydroxy-3,5-dimethoxyphenyl)-3-(2-hydroxyphenyl)-4,5-dihydro-pyrazol-1-yl]-methanone* (**b17**). Yield: 66.67%; M.p. 169.2–170.1 °C. IR (KBr) ν_max_ (cm^−1^): 3392, 3262 (aromatic –OH), 2943, 2842 (aliphatic C–H asymmetric), 1690 (C=O), 1616, 1514, 1440, 1376 (C=N and C=C), 1338, 1237, 1113, 1049 (C–N). ^1^H-NMR (400 MHz, δ ppm, DMSO-*d*_6_): 3.39–3.40 (1H, m, pyrazoline C_4_–H_A_), 3.70 (6H, s, –OCH_3_), 3.96–4.03 (1H, m, pyrazoline C_4_–H_B_), 5.63–5.67 (1H, m, pyrazoline C_5_–H*_x_*), 6.53 (2H, s), 6.98–7.01 (2H, m), 7.35–7.39 (1H, m), 7.44–7.52 (2H, m), 7.85–7.87 (1H, m), 8.03–8.08 (2H, m), 8.37 (1H, s), 8.40 (1H, s, –OH), 10.23 (1H, s, –OH). ^1^^3^C-NMR (600 MHz, δ ppm, DMSO-*d*_6_): 40.41, 44.01, 56.02, 103.17, 116.72, 117.00, 119.64, 122.54, 124.85, 125.49, 126.63, 129.30, 130.80, 132.04, 132.07, 135.01, 135.23, 137.92, 141.85, 148.11, 156.77, 156.86, 158.08. MS (ESI) (*m*/*z*): 475 [M + H]^+^. HRMS: *m*/*z* [M − H]¯ calcd for C_26_H_22_N_2_O_5_S: 473.1177; found: 474.1249.

*4-[5-(2-Hydroxyphenyl)-2-phenyl-3,4-dihydro-2H-pyrazol-3-yl]-2,6-dimethoxy-phenol* (**b18**). Yield: 48.46%; M.p. 169.7–170.8 °C. IR (KBr) ν_max_ (cm^−1^): 3400 (aromatic –OH), 2939, 2842 (aliphatic C–H asymmetric), 1493, 1464, 1431 (C=N and C=C), 1373, 1245, 1113, 1026 (C–N). ^1^H-NMR (400 MHz, δ ppm, DMSO-*d*_6_): 3.25–3.30 (1H, m, pyrazoline C_4_–H_A_), 3.70 (6H, s, –OCH_3_), 4.00–4.07 (1H, m, pyrazoline C_4_-H_B_), 5.25–5.29 (1H, m, pyrazoline C_5_–H*_x_*), 6.63 (2H, s), 6.81 (1H, t, *J* = 8.00 Hz), 6.92–7.01 (4H, m), 7.23 (2H, t, *J* = 8.00 Hz), 7.30 (1H, t, *J* = 8.00 Hz), 7.44 (1H, d, *J* = 8.00 Hz), 8.39 (1H, s, –OH), 10.62 (1H, s, –OH). MS (ESI) (*m*/*z*): 391 [M + H]^+^.

*1-Phenyl-3-p-tolyl-5-(3,4,5-trimethoxyphenyl)-4,5-dihydro-1H-pyrazole* (**b19**). Yield: 50.39%; M.p. 137.9–140.3 °C. IR (KBr) ν_max_ (cm^−1^): 2934, 2834 (aliphatic C–H asymmetric), 1495, 1462, 1418 (C=N and C=C), 1348, 1319, 1235, 1125 (C–N). ^1^H-NMR (400 MHz, δ ppm, DMSO-*d*_6_): 2.34 (3H, s, –CH_3_), 3.09–3.15 (1H, m, pyrazoline C_4_–H_A_), 3.63 (3H, s, –OCH_3_), 3.70 (6H, s, –OCH_3_), 3.83–3.91 (1H, m, pyrazoline C_4_–H_B_), 5.29–5.33 (1H, m, pyrazoline C_5_-H*_x_*), 6.62 (2H, s), 6.74 (1H, t, *J* = 8.00 Hz), 7.04 (2H, d, *J* = 8.00 Hz), 7.18 (2H, t, *J* = 8.00 Hz), 7.25 (2H, d, *J* = 8.00 Hz), 7.65 (2H, d, *J* = 8.00 Hz). MS (ESI) (*m*/*z*): 403 [M + H]^+^.

**b1–19** ^1^H-NMR, **b17** ^13^H-NMR and **b17** HRMS are as provided as Supplementary Materials.

### 3.4. Pharmacology

#### 3.4.1. Cell Culture and Treatment

Primary hepatocytes were isolated from male SD rats weighing 250–300 g using a modified in situ collagenase perfusion method and then the cells were cultured in Dulbecco’s Modified Eagle’s Medium (DMEM) with 2 g/L bovine serum albumin (BSA) and 50 mg/L gentamicin. HepG-2 cells were incubated in RPMI 1640 medium supplemented with 10% fetal bovine serum (FBS) and 1% penicillin–streptomycin liquid. Cells were incubated at 37 °C in a humidified atmosphere of 95% air and 5% CO_2_. Stock solutions of compounds and cisplatin were prepared in dimethyl sulfoxide (DMSO), then added to fresh culture medium to obtain final concentrations.

#### 3.4.2. MTT Assay

To evaluate the compounds’ cytotoxicity, 50 µM and 100 µM concentrations of each compound were used to target the more effective compounds for further study. Next, compounds **b14** and **b18** were prepared in five different concentrations (20 µM, 40 µM, 60 µM, 80 µM, 100 µM), and compounds **b15**, **b16**, and **b17**, along with cisplatin, were prepared in seven different concentrations (2.5 µM, 5 µM, 7.5 µM, 10 µM, 20 µM, 40 µM, 60 µM). A suspension of growing cells (50,000/mL) was prepared, and 100 µL/well dispensed into 96-well plates, yielding a density of 5000 cells/well. The cells were incubated for 24 h before adding the pyrazoline derivatives. The optimal cell number for cytotoxicity assays was determined in preliminary experiments. At the end of the incubation period, the medium was removed and 100 µL of different concentrations of pyrazoline derivatives were added to wells for 24 h and 48 h. Following the exposure period, 20 µL of MTT (5 mg/mL) were added to cells and incubated for a further 4 h at 37 °C. The medium was removed and the formazan crystals were solubilized by adding 150 µL DMSO to each well. After 10 min of shaking, the absorbance was measured at 550 nm using a FilterMax F5 Multi-Mode Microplate Reader (Energy Chemical, Shanghai, China). Cells were treated with each compound with four replicates per concentration, and each experiment was conducted at least three times. Dose–inhibition rate curves and IC_50_ values, defined as the drug concentrations which reduced absorbance to 50% of control values, were then generated.

#### 3.4.3. MTS Assay

The cytotoxicological activity of compounds **b5**, **b9**, and **b14**–**18** were evaluated against primary hepatocytes using the MTS assay (Cell Titer 96^®^ Aqueous Cell Proliferation Assay, Promega, Cat. No. G5421) as a fast and sensitive quantification of cell proliferation and viability. The concentrations of compounds were prepared in seven different concentrations (2.5 µM, 5 µM, 10 µM, 20 µM, 40 µM, 80 µM, 160 µM). Cells were seeded into 96-well plates at a density of 26,000 cells/well. The plates were incubated for 24 h prior to any treatment. At the end of this period, the medium was removed and 100 µL of different concentrations of pyrazoline derivatives were added to wells for 48 h. Following the exposure period, 20 μL of the combined MTS/PMS solution was added to cells and incubated for a further 2 h at 37 °C, then the absorbance was recorded at 490 nm using an ELISA Plate Reader (ELISA, Emeryville, CA, USA). The values of IC_50_, the effective concentration at which 50% of the primary hepatocytes were inhibited, were calculated to evaluate the cytotoxic activities.

#### 3.4.4. Cell Cycle Analysis

Based on the results of the cytotoxicity assay, compound **b17** was selected for further mechanistic study with HepG-2 cells. For flow cytometry analysis of DNA content, approximately 1.5 × 10^5^ HepG-2 cells/well in exponential growth mode were plated in 6-well plates and allowed to adhere, then treated with different concentrations of compound **b17** for 24 h. After incubation, the cells were collected, centrifuged, and fixed with ice-cold ethanol (70%). The cells were treated with RNase A and stained with PI. Samples were analyzed on a BD Accuri C6 flow cytometer (BD, New York, NY, USA). DNA histograms were analyzed using ModFit for Windows.

#### 3.4.5. Annexin-V Assay

Approximately 1.5 × 10^5^ HepG-2 cells/well were plated in 6-well plates and allowed to adhere. After 24 h, the medium was replaced with fresh culture medium containing compound **b17** at final concentrations of 0 µM, 0.9 µM, 2.7 µM, and 4.5 µM. Cells were harvested after 12 h. The cells were trypsinized, washed in phosphate-buffered saline (PBS), and centrifuged at 1000 rpm for 5 min. The cells were then resuspended in 200 µL of staining solution (containing 10 µL PI and 5 µL Annexin V–PE in binding buffer), mixed gently, and incubated for 15 min at 25 °C in the dark. The samples were later analyzed with a BD Accuri™ C6 flow cytometer.

## 4. Conclusions

In the present study, we synthesized 19 new pyrazoline derivatives and investigated their antiproliferative effects on HepG-2 cells. We found that compound **b17** was the most effective anticancer agent following a 48 h exposure with an IC_50_ value of 3.57 µM compared with the cisplatin value of 8.45 µM. Compound **b17** was therefore selected for cell cycle analysis and apoptosis/necrosis evaluation. We observed that HepG-2 cells treated with compound **b17** could be arrested in the G_2_/M phase. In addition, compound **b17** can be regarded as a potent inducer of apoptosis in the cells. These results provide an important foundation for further development of compound **b17** as a potent antitumor agent. We also found that compounds with a heterocyclic ring, such as **b15** and **b16**, exhibited better pharmacological activity than most other pyrazoline derivatives synthesized in this study. Compounds **b15** and **b16** will be tested further to improve characterization of their antitumor activity.

## Figures and Tables

**Table 1:** Structure of the compounds **a1**–**5**.

Compounds	R_1_	R_2_	R_3_	R_4_	R_5_
**a1**	–OCH_3_	–OH	–OCH_3_	–OH	H
**a2**	–OCH_3_	–OH	–OCH_3_	H	–NO_2_
**a3**	–Br	–OH	–OCH_3_	–OH	H
**a4**	–Br	–OH	–OCH_3_	H	–NO_2_
**a5**	–OCH_3_	–OCH_3_	–OCH_3_	H	–CH_3_

**Table 2:** Structure of the compounds **b1**–**19**.

Compounds	R_1_	R_2_	R_3_	R_4_	R_5_	R_6_
**b1**	–OCH_3_	–OH	–OCH_3_	–OH	H	H
**b2**	–OCH_3_	–OH	–OCH_3_	H	–NO_2_	H
**b3**	–Br	–OH	–OCH_3_	–OH	H	H
**b4**	–Br	–OH	–OCH_3_	H	–NO_2_	H
**b5**	–OCH_3_	–OH	–OCH_3_	–OH	H	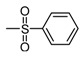
**b6**	–OCH_3_	–OH	–OCH_3_	–OH	H	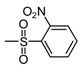
**b7**	–OCH_3_	–OH	–OCH_3_	–OH	H	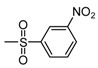
**b8**	–OCH_3_	–OH	–OCH_3_	–OH	H	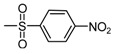
**b9**	–OCH_3_	–OH	–OCH_3_	–OH	H	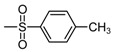
**b10**	–OCH_3_	–OH	–OCH_3_	–OH	H	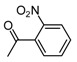
**b11**	–OCH_3_	–OH	–OCH_3_	–OH	H	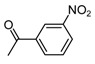
**b12**	–OCH_3_	–OH	–OCH_3_	–OH	H	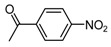
**b13**	–OCH_3_	–OH	–OCH_3_	–OH	H	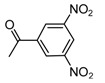
**b14**	–OCH_3_	–OH	–OCH_3_	–OH	H	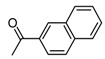
**b15**	–OCH_3_	–OH	–OCH_3_	–OH	H	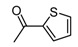
**b16**	–OCH_3_	–OH	–OCH_3_	–OH	H	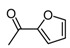
**b17**	–OCH_3_	–OH	–OCH_3_	–OH	H	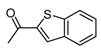
**b18**	–OCH_3_	–OH	–OCH_3_	–OH	H	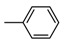
**b19**	–OCH_3_	–OCH_3_	–OCH_3_	–H	–CH_3_	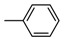

**Table 3:** The cytotoxic effects of the compounds **b1**–**19** on HepG-2 cell line and primary hepatocytes.

Compounds	IC_50_/μmol·L^−1^		Compounds	IC_50_/μmol·L^−1^	
	HepG-2	Primary Hepatocytes		HepG-2	Primary Hepatocytes
**b1**	>50	—	**b12**	>50	—
**b2**	>50	—	**b13**	>50	—
**b3**	>50	—	**b14**	17.99 ± 1.37	22.65 ± 1.21
**b4**	>50	—	**b15**	4.51 ± 1.49	19.24 ± 0.08
**b5**	28.76 ± 1.32	35.13 ± 2.21	**b16**	4.61 ± 1.27	20.73 ± 1.72
**b6**	>50	—	**b17**	3.57 ± 1.39	33.47 ± 2.33
**b7**	>50	—	**b18**	28.47 ± 1.34	50.71 ± 3.21
**b8**	>50	—	**b19**	>50	—
**b9**	12.01 ± 1.83	29.66 ± 2.43	**Cisplatin**	8.45 ± 1.05	—
**b10**	>50	—			
**b11**	>50	—			

“—”: Not determined.

## Supplementary Materials

Supplementary materials are available online.
