# Supplementary material for: Synthesis and Evaluation of New Pyrazoline Derivatives as Potential Anticancer Agents in HepG-2 Cell Line
Source: Molecules. 2017 Mar 16;22(3):467. doi: 10.3390/molecules22030467 (PMC6155299; doi:10.3390/molecules22030467)
Supplement: Supplementary file 1 [file molecules-22-00467-s001.zip › Supplementary Materials/b1-19(1H-NMR).pdf]

Synthesis and Evaluation of New Pyrazoline Derivatives as Potential Anticancer Agents in HepG-2 Cell Line

Weijie Xu [1,†], Ying Pan [1,†], Hong Wang [1,†], Haiyan Li [2], Qing Peng [3], Duncan Wei [1], Cheng Chen [1] and Jinhong Zheng [1,*]

1 Department of Chemistry, Shantou University Medical College, Shantou 515041, Guangdong, China; 13536920534@163.com (W.X.); ypan@stu.edu.cn (Y.P.); 13592851552@163.com (H.W.); weiduncan2012@163.com (D.W.); 13411963192@163.com (C.C.); jhzheng@stu.edu.cn (J.Z)

2 Department of Pharmacology, Shantou University Medical College, Shantou 515041, Guangdong, China; 13066360950@163.com (H.L.)

3 Department of Hepatobiliary Surgery II, Zhujiang Hospital of Southern Medical University, Guangzhou 510280, Guangdong, China; zhujiangzhuanhua@163.com (Q.P.)

* Correspondence: jhzheng@stu.edu.cn; Tel.: +86-754-8890-0499; Fax: +86-754-8855-7562

† These authors contributed equally to this work.

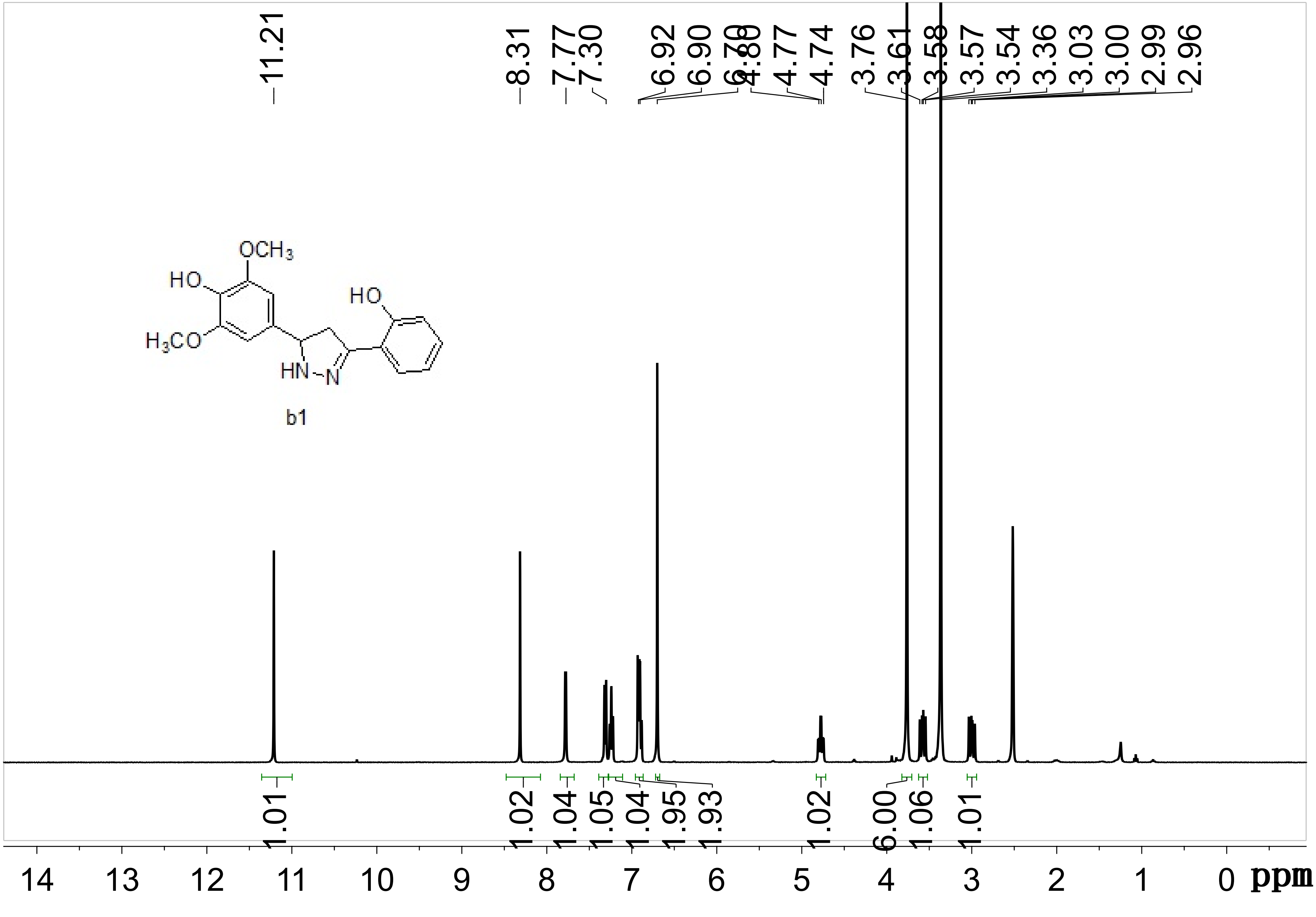
11.21
8.31
7.77
7.30
6.92
6.90
6.70
4.80
4.77
4.74
3.76
3.61
3.58
3.57
3.54
3.36
3.03
3.00
2.99
2.96
OCH3
HO
H3CO
HO
HN
N
b1
1.01
1.02
1.04
1.05
1.04
1.95
1.93
1.02
6.00
1.06
1.01
14
13
12
11
10
9
8
7
6
5
4
3
2
1
0
ppm

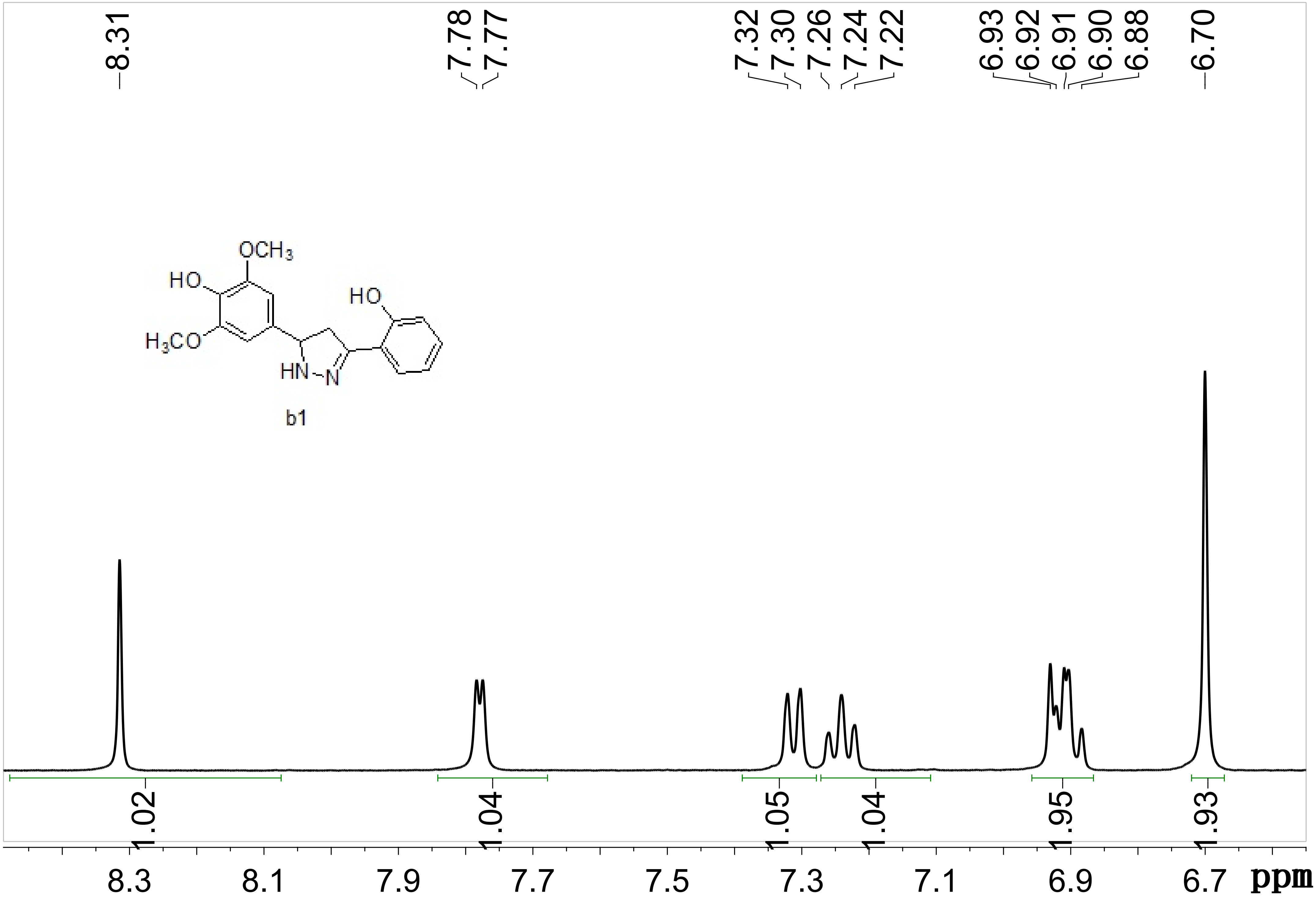
8.31
7.78
7.77
7.32
7.30
7.26
7.24
7.22
6.93
6.92
6.91
6.90
6.88
6.70
OCH3
HO
HO
H3CO
HN
N
b1
1.02
1.04
1.05
1.04
1.95
1.93
8.3
8.1
7.9
7.7
7.5
7.3
7.1
6.9
6.7
ppm

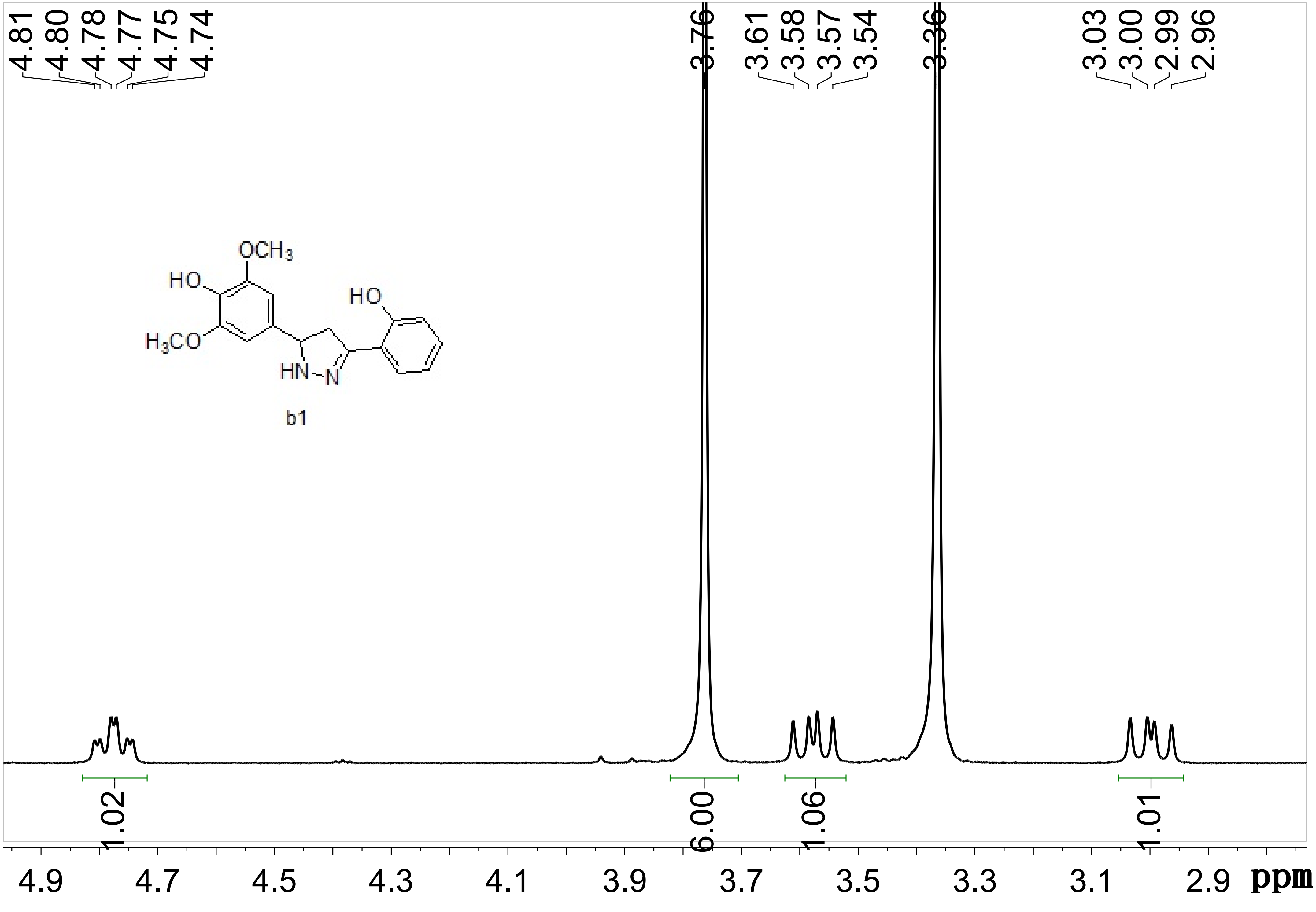

4.81
4.80
4.78
4.77
4.75
4.74
3.76
3.61
3.58
3.57
3.54
3.36
3.03
3.00
2.99
2.96
OCH3
HO
H3CO
HO
HN
N
b1
1.02
6.00
1.06
1.01
4.9
4.7
4.5
4.3
4.1
3.9
3.7
3.5
3.3
3.1
2.9
ppm

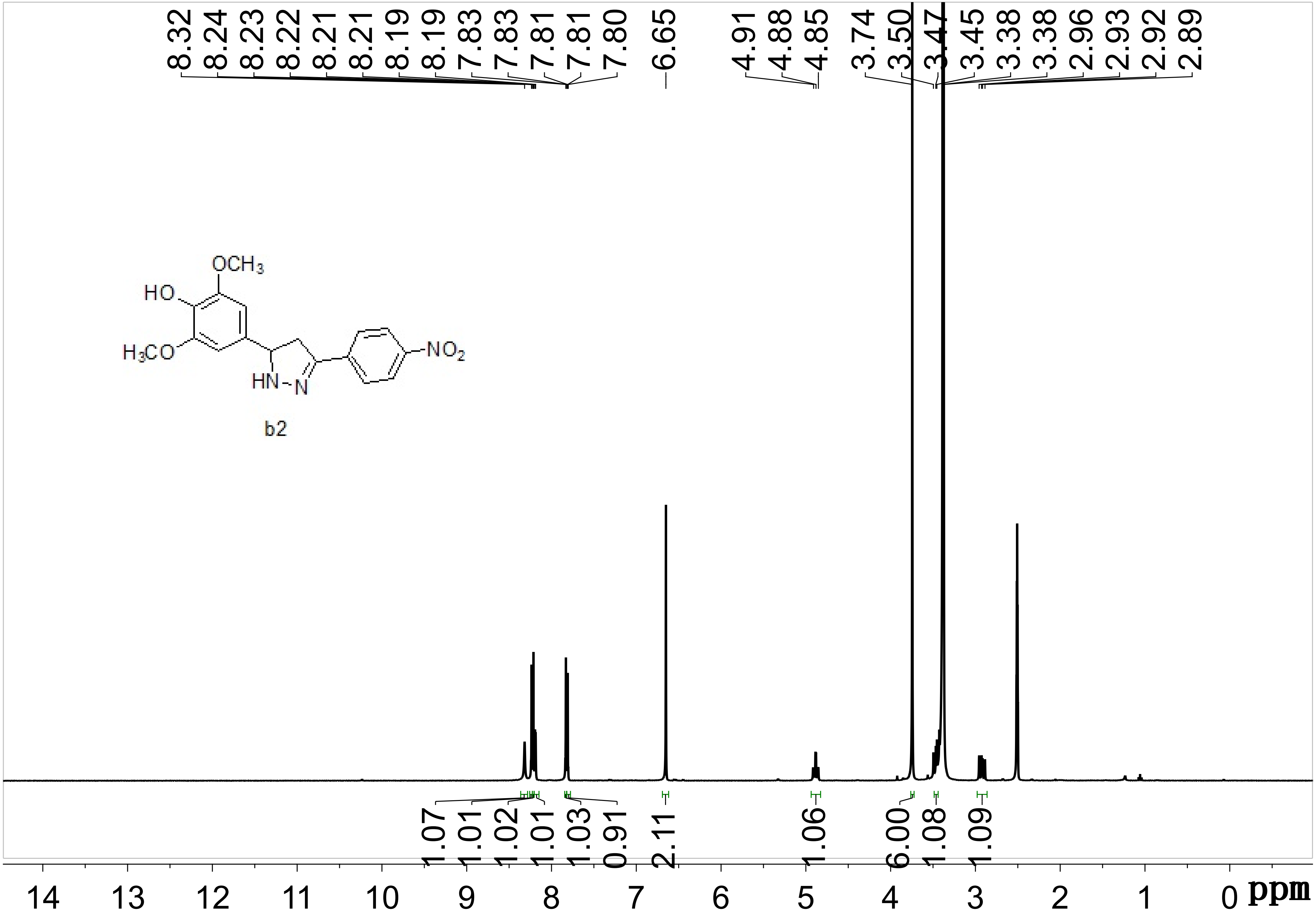

8.32 8.24 8.23 8.22 8.21 8.21 8.19 8.19 7.83 7.83 7.81 7.81 7.80
6.65
4.91 4.88 4.85
3.74 3.50 3.47 3.45 3.38 3.38 2.96 2.93 2.92 2.89
OCH3
HO
H3CO
HN
N
NO2
b2
1.07 1.01 1.02 1.01 1.03 0.91
2.11
1.06
6.00 1.08
1.09
14 13 12 11 10 9 8 7 6 5 4 3 2 1 0 ppm

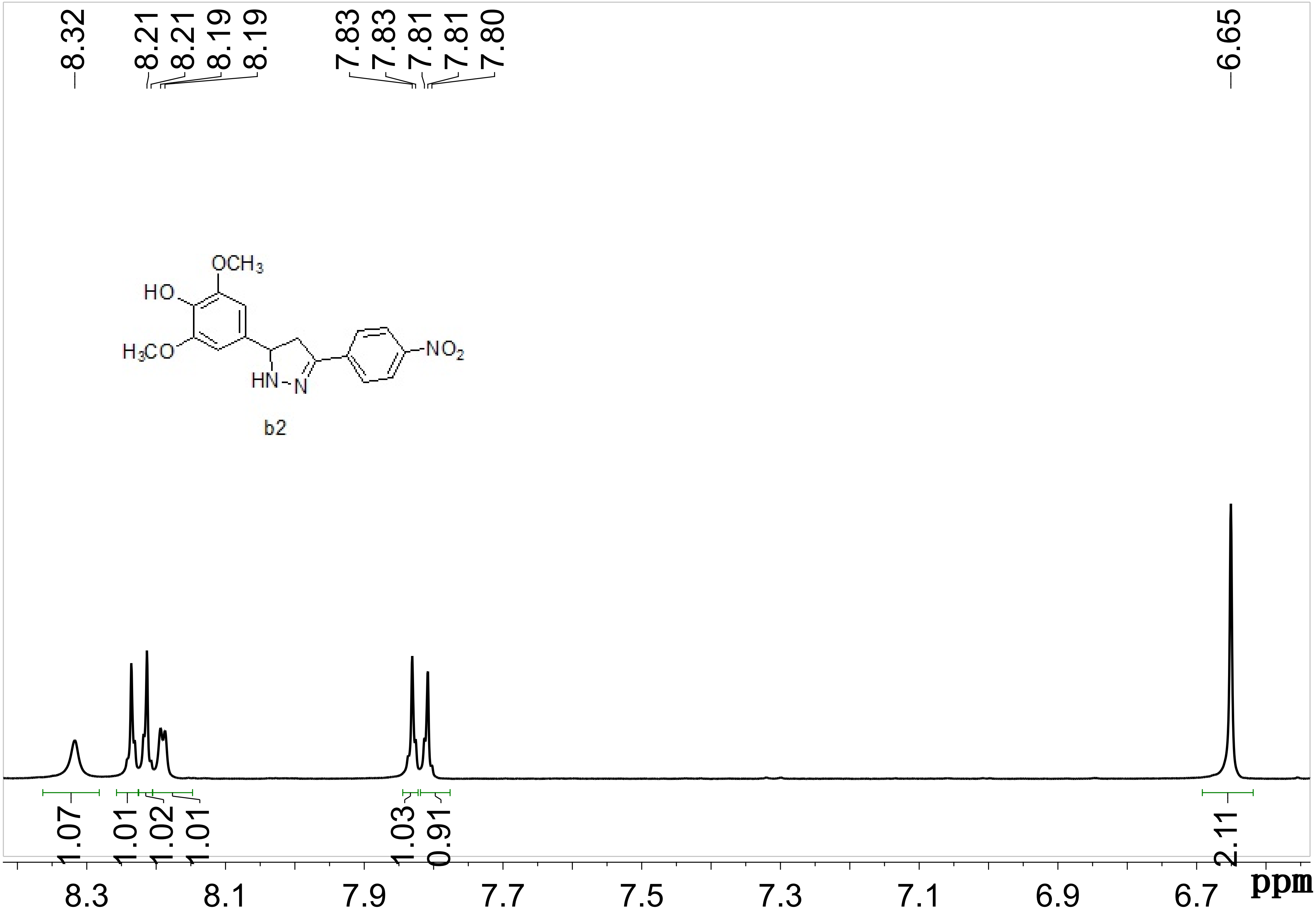
8.32
8.21
8.21
8.19
8.19
7.83
7.83
7.81
7.81
7.80
6.65
OCH3
HO
H3CO
NO2
HN
N
b2
1.07
1.01
1.02
1.01
1.03
0.91
2.11
8.3
8.1
7.9
7.7
7.5
7.3
7.1
6.9
6.7
ppm

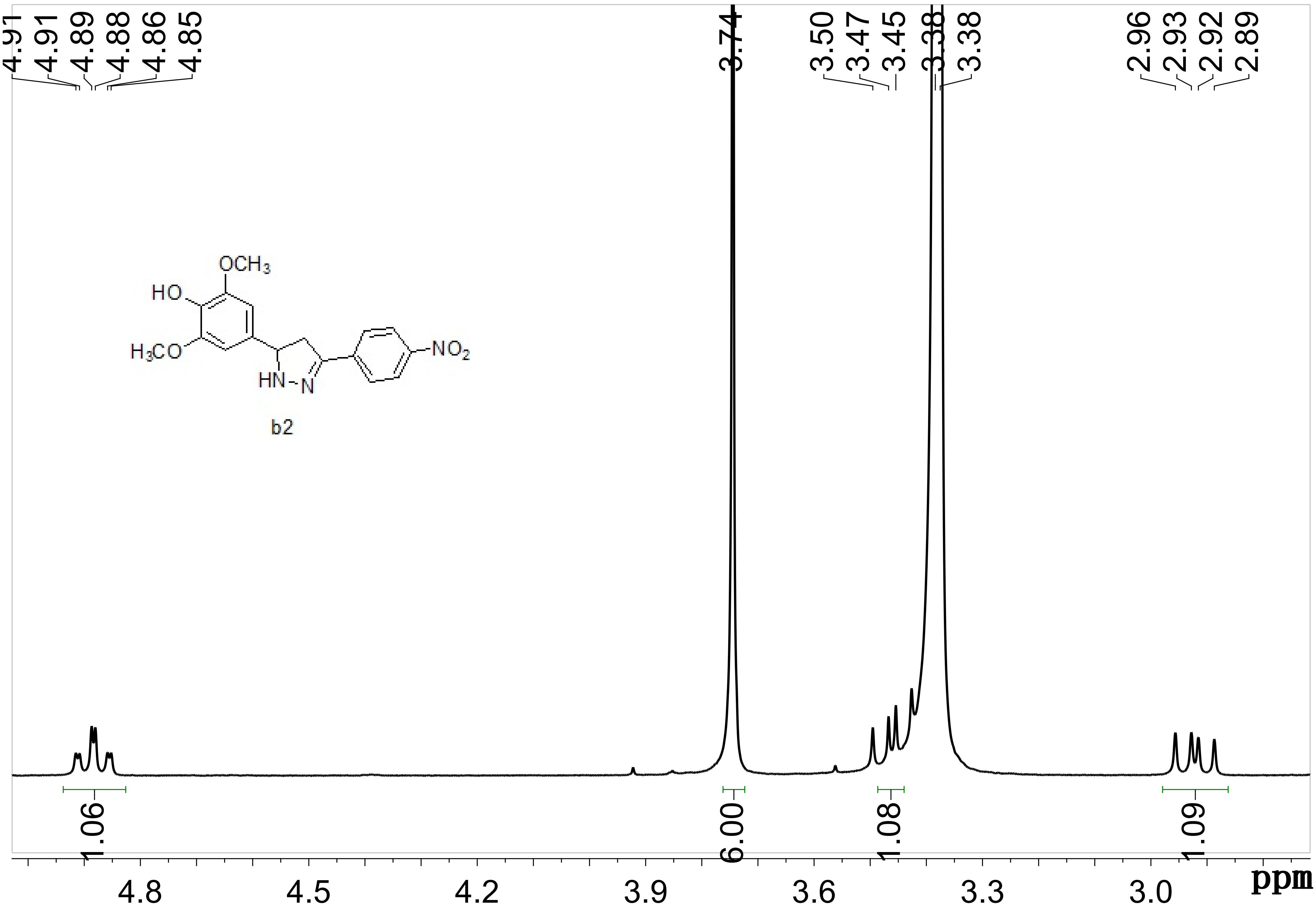
4.91
4.91
4.89
4.88
4.86
4.85
3.74
3.50
3.47
3.45
3.38
3.38
2.96
2.93
2.92
2.89
OCH3
HO
H3CO
HN
N
NO2
b2
1.06
6.00
1.08
1.09
4.8
4.5
4.2
3.9
3.6
3.3
3.0
ppm

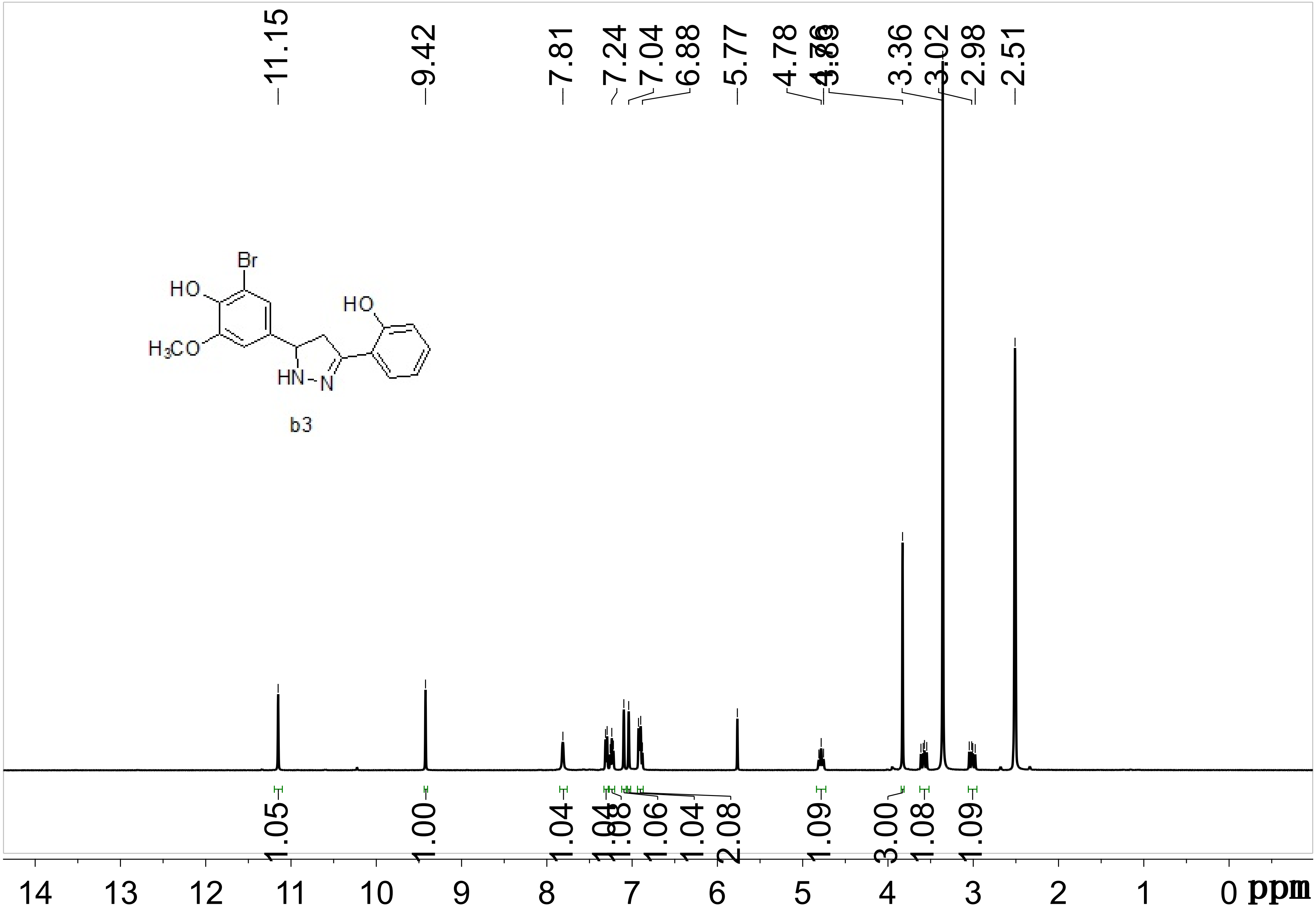

11.15
9.42
7.81
7.24
7.04
6.88
5.77
4.78
4.76
3.36
3.02
2.98
2.51
Br
HO
HO
H3CO
HN
N
b3
1.05
1.00
1.04
1.04
1.08
1.06
1.04
2.08
1.09
3.00
1.08
1.09
14 13 12 11 10 9 8 7 6 5 4 3 2 1 0 ppm

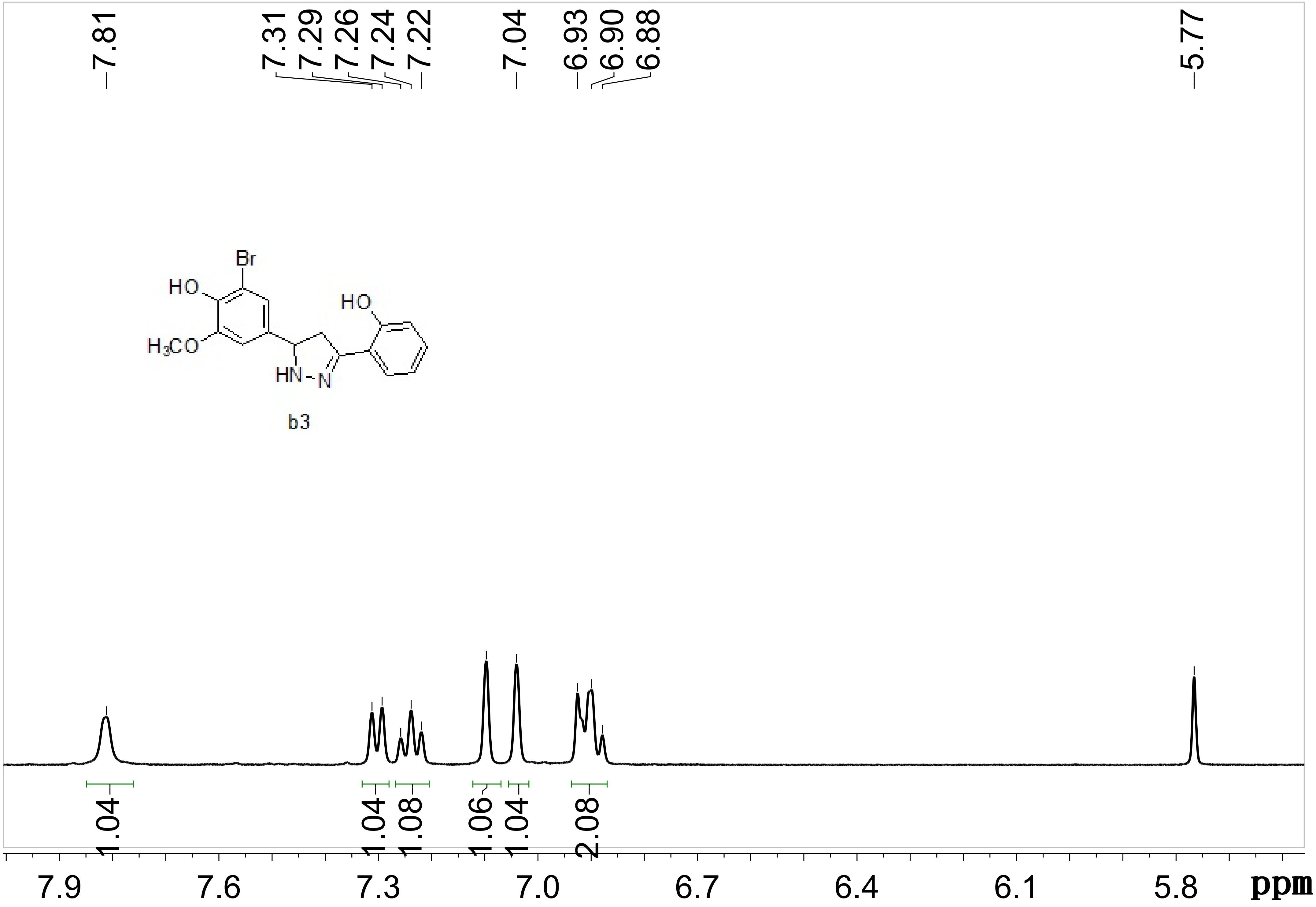
7.81
7.31
7.29
7.26
7.24
7.22
7.04
6.93
6.90
6.88
5.77
Br
HO
HO
H3CO
HN
N
b3
1.04
1.04
1.08
1.06
1.04
2.08
7.9
7.6
7.3
7.0
6.7
6.4
6.1
5.8
ppm

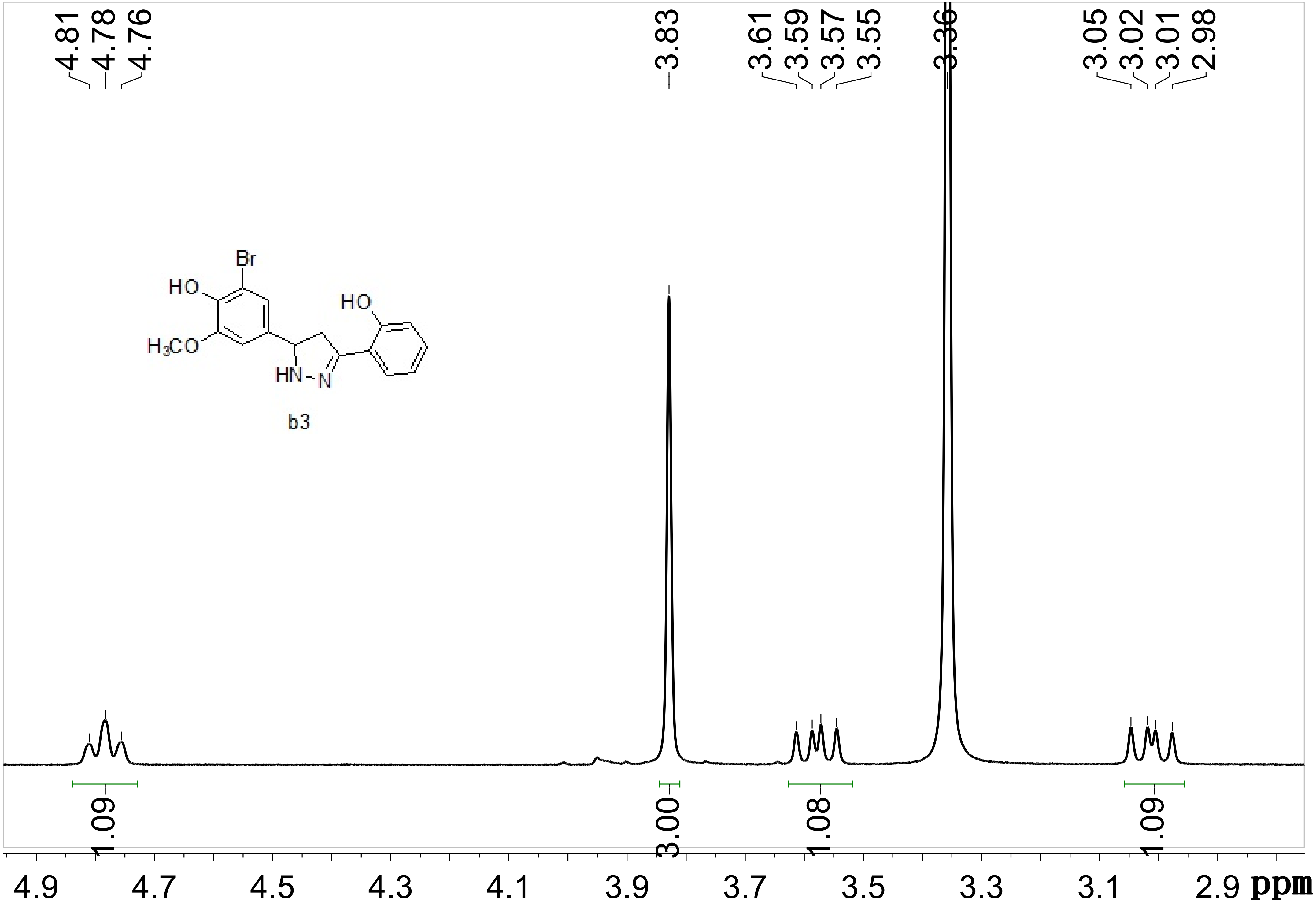
4.81
4.78
4.76
3.83
3.61
3.59
3.57
3.55
3.36
3.05
3.02
3.01
2.98
Br
HO
HO
H3CO
HN
N
b3
1.09
3.00
1.08
1.09
4.9
4.7
4.5
4.3
4.1
3.9
3.7
3.5
3.3
3.1
2.9
ppm

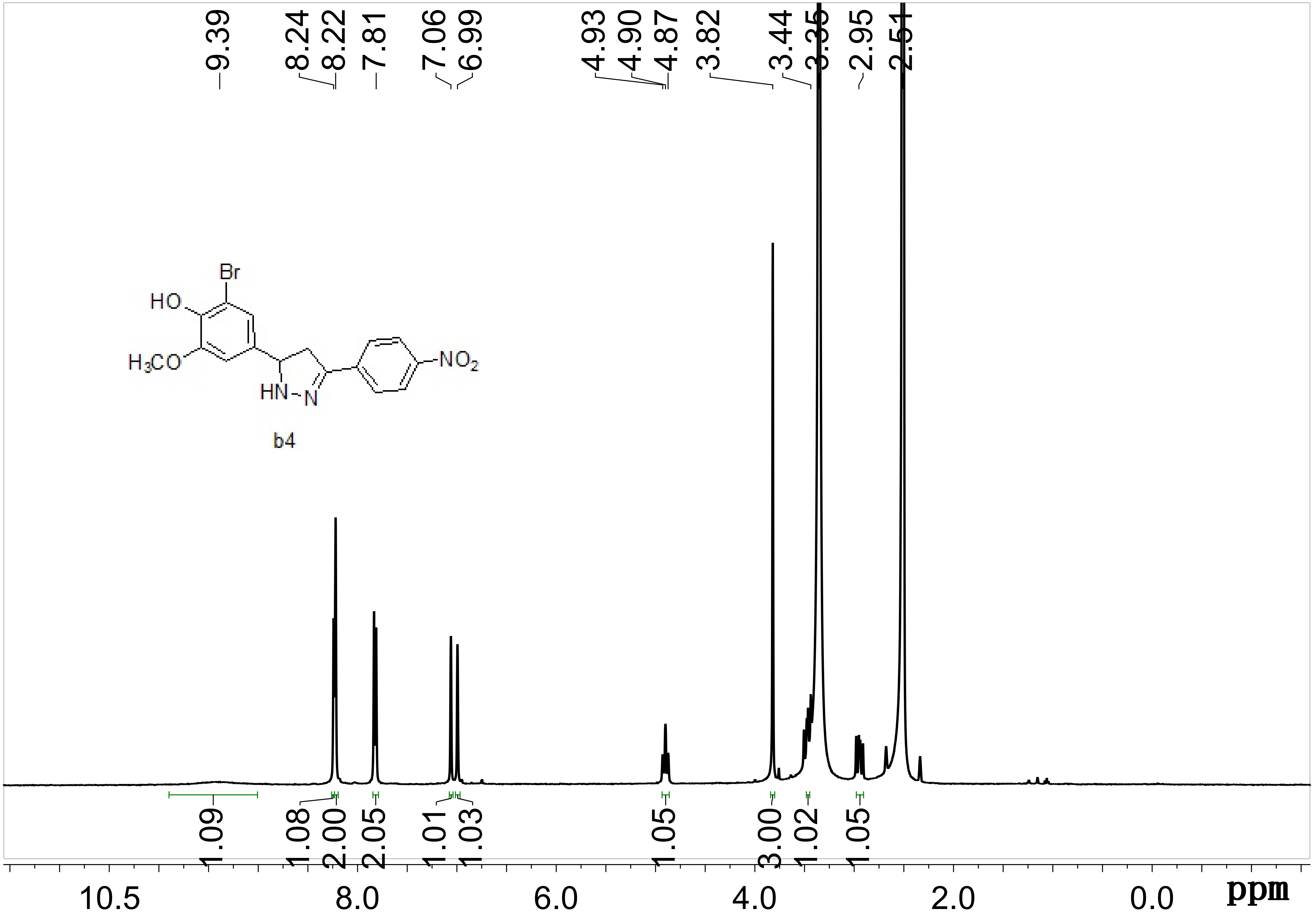
9.39
8.24
8.22
7.81
7.06
6.99
4.93
4.90
4.87
3.82
3.44
2.95
Br
HO
H_3CO
NO_2
HN
N
b4
1.09
1.08
2.00
2.05
1.01
1.03
1.05
3.00
1.02
1.05
10.5
8.0
6.0
4.0
2.0
0.0
ppm

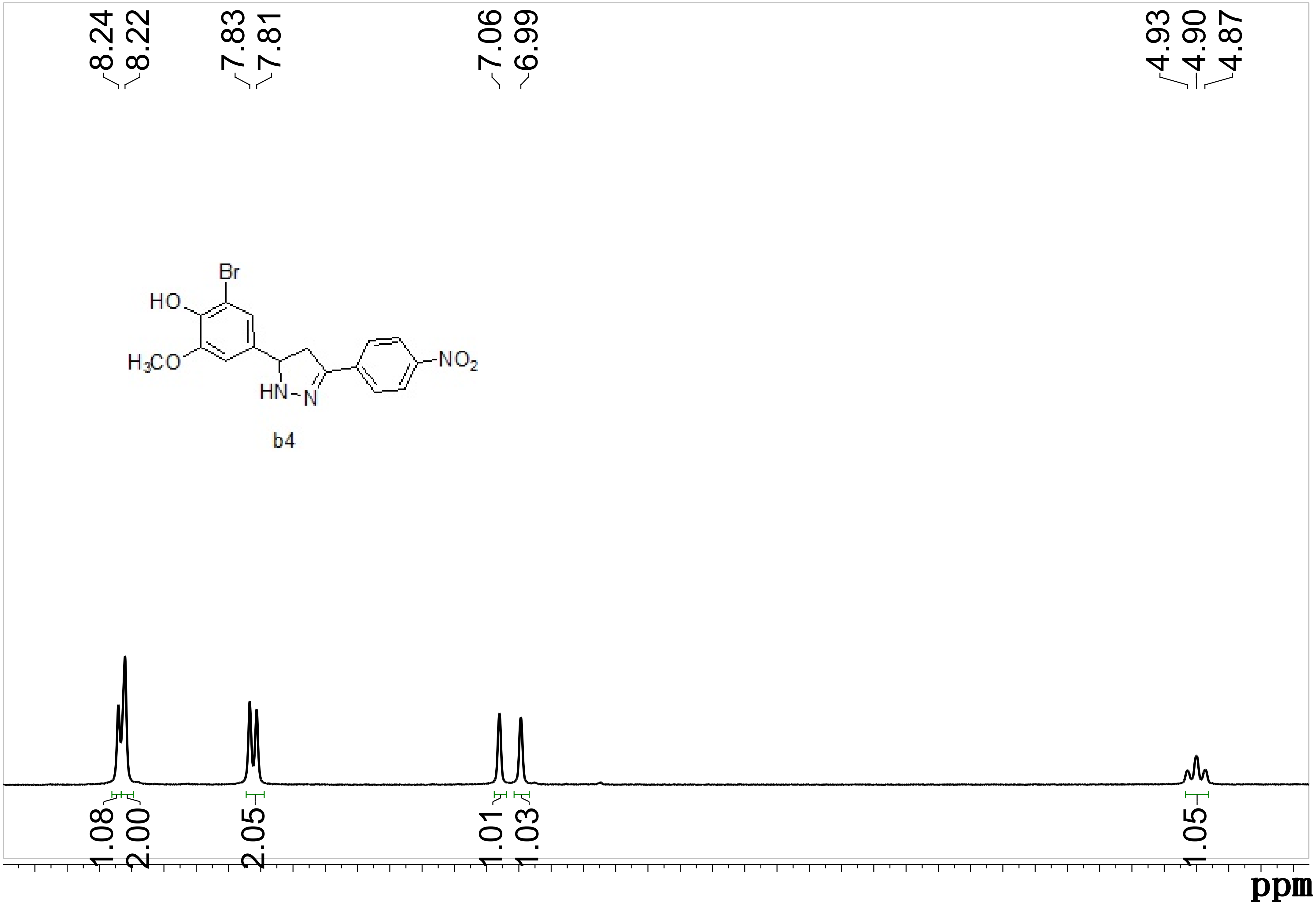
8.24
8.22
7.83
7.81
7.06
6.99
4.93
4.90
4.87
Br
HO
H3CO
HN
N
NO2
b4
1.08
2.00
2.05
1.01
1.03
1.05
ppm

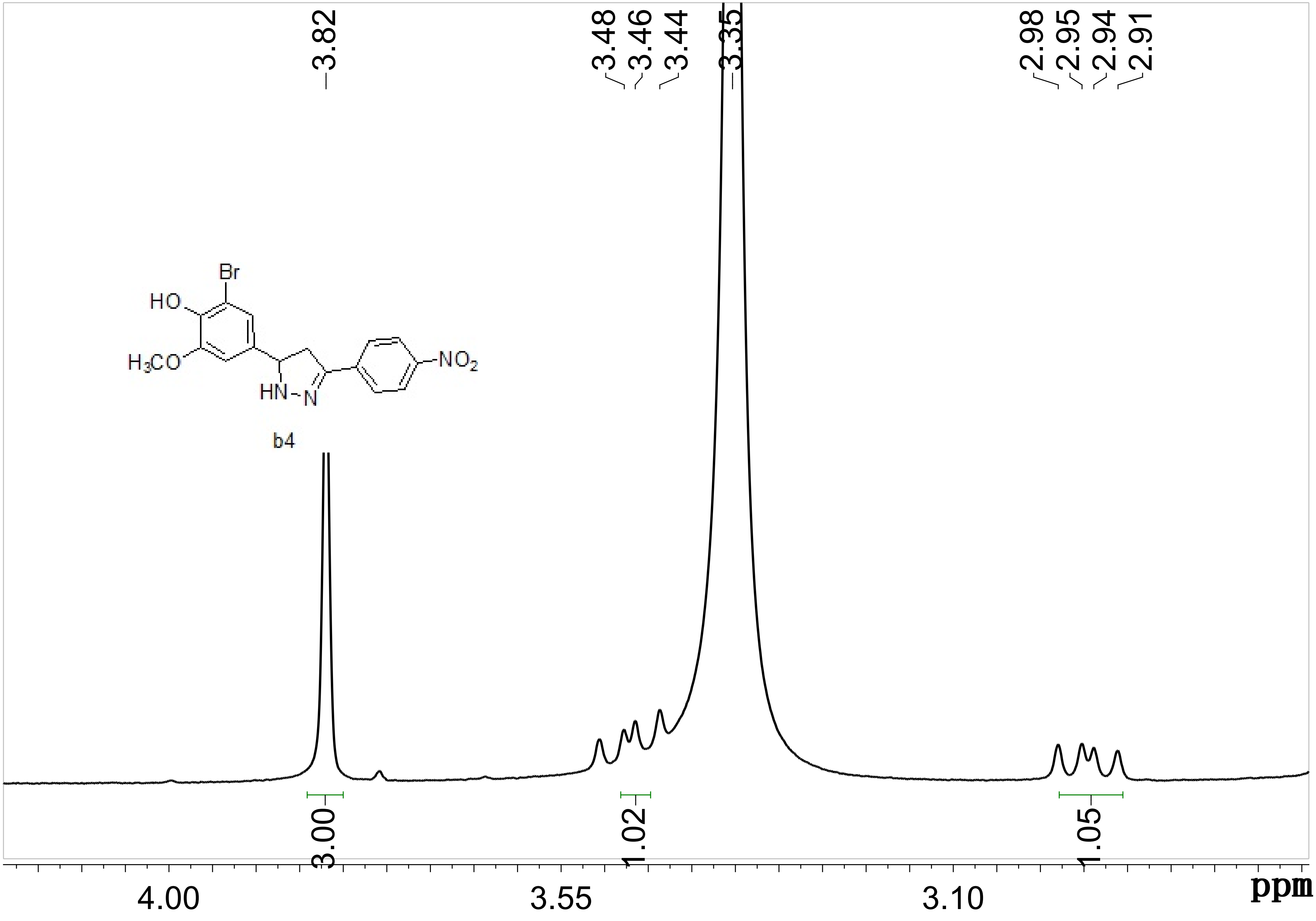
3.82
3.48
3.46
3.44
3.35
2.98
2.95
2.94
2.91
Br
HO
H3CO
NO2
HN
N
b4
3.00
1.02
1.05
4.00
3.55
3.10
ppm

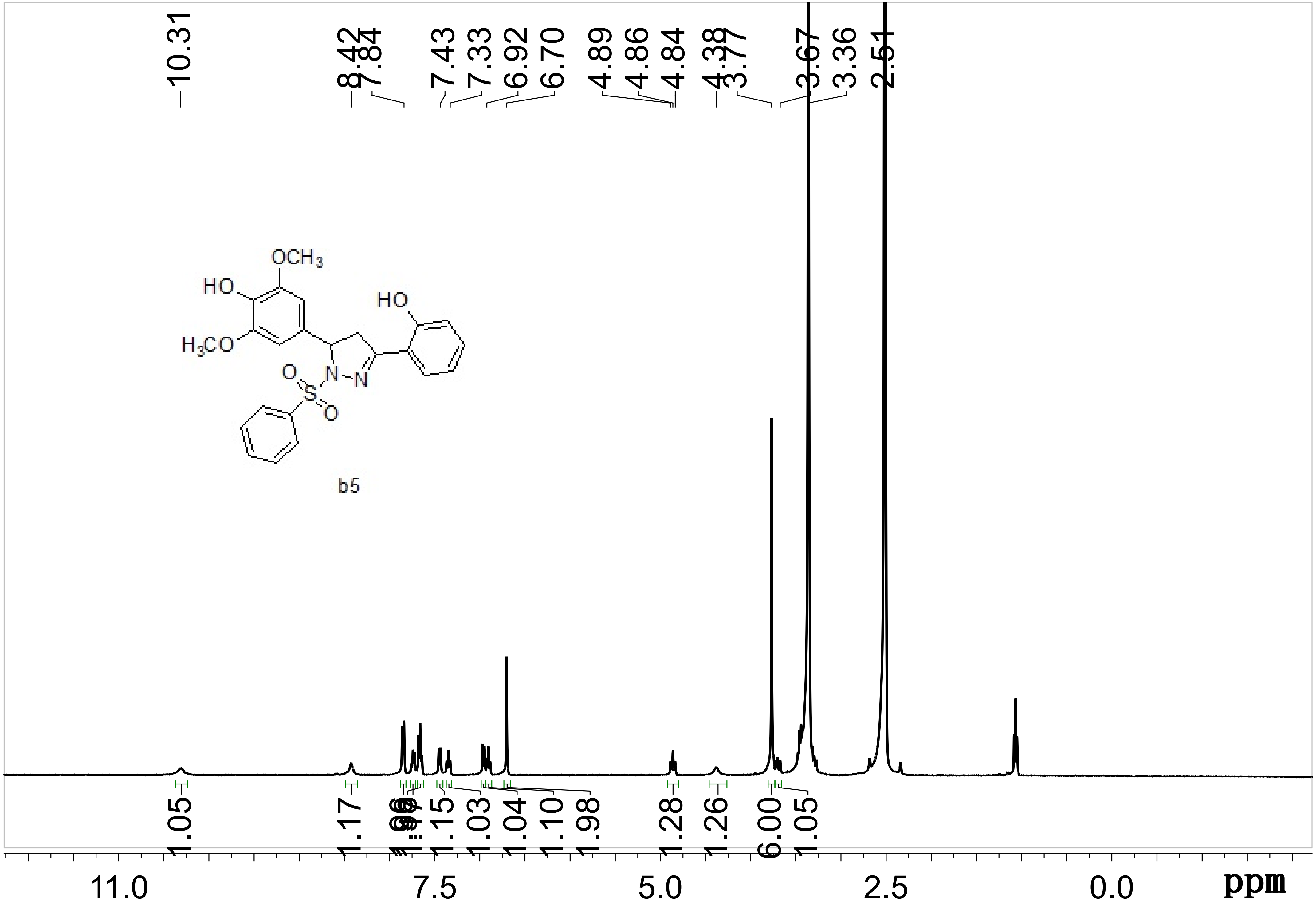
10.31
8.42
7.84
7.43
7.33
6.92
6.70
4.89
4.86
4.84
4.38
3.77
3.67
3.36
2.51
OCH3
HO
H3CO
HO
O
S
O
N
N
b5
1.05
1.17
1.15
1.03
1.04
1.10
1.98
1.28
1.26
6.00
1.05
11.0
7.5
5.0
2.5
0.0
ppm

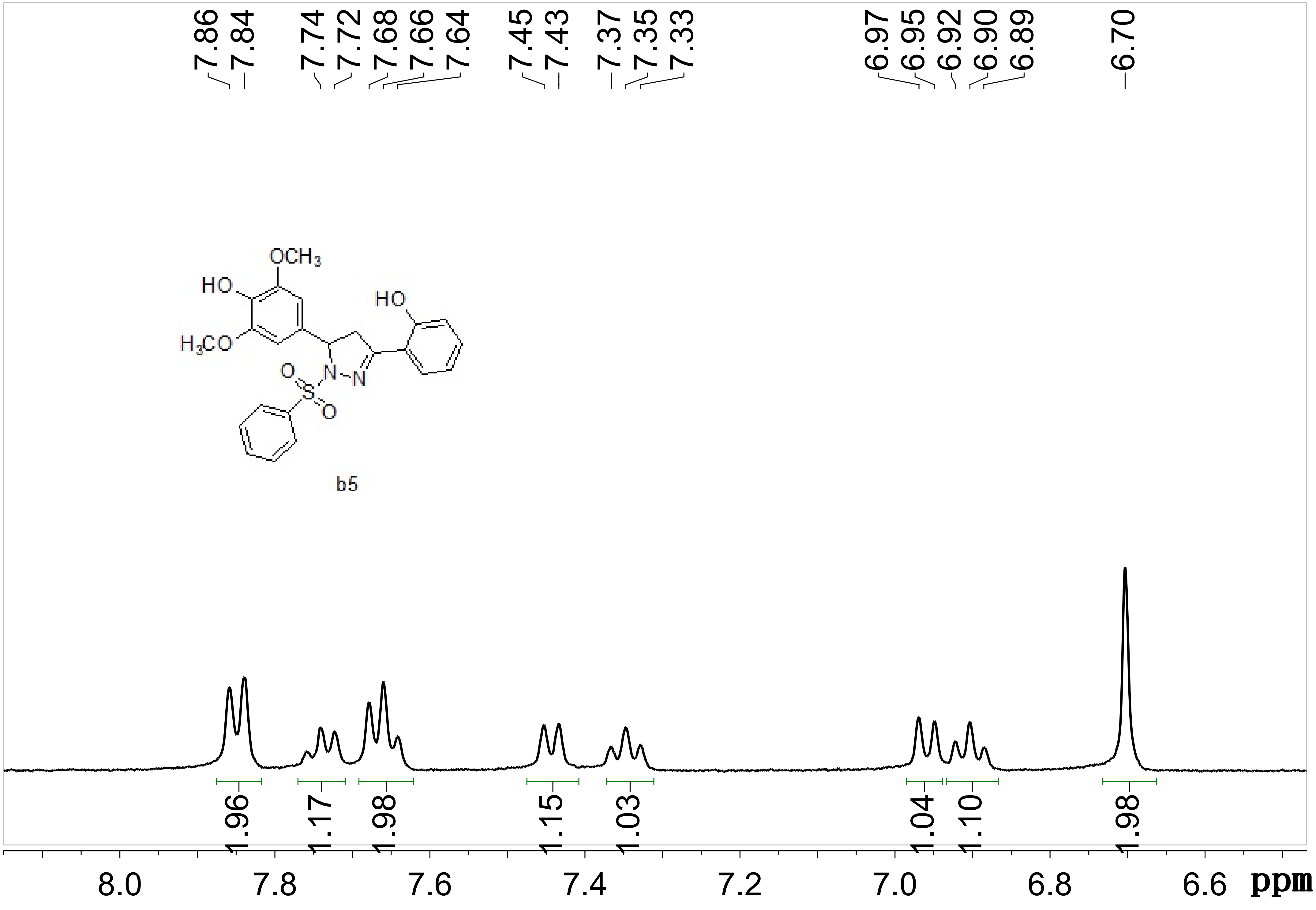
7.86
7.84
7.74
7.72
7.68
7.66
7.64
7.45
7.43
7.37
7.35
7.33
6.97
6.95
6.92
6.90
6.89
6.70
OCH3
HO
HO
H3CO
N
N
O
S
O
b5
1.96
1.17
1.98
1.15
1.03
1.04
1.10
1.98
8.0
7.8
7.6
7.4
7.2
7.0
6.8
6.6
ppm

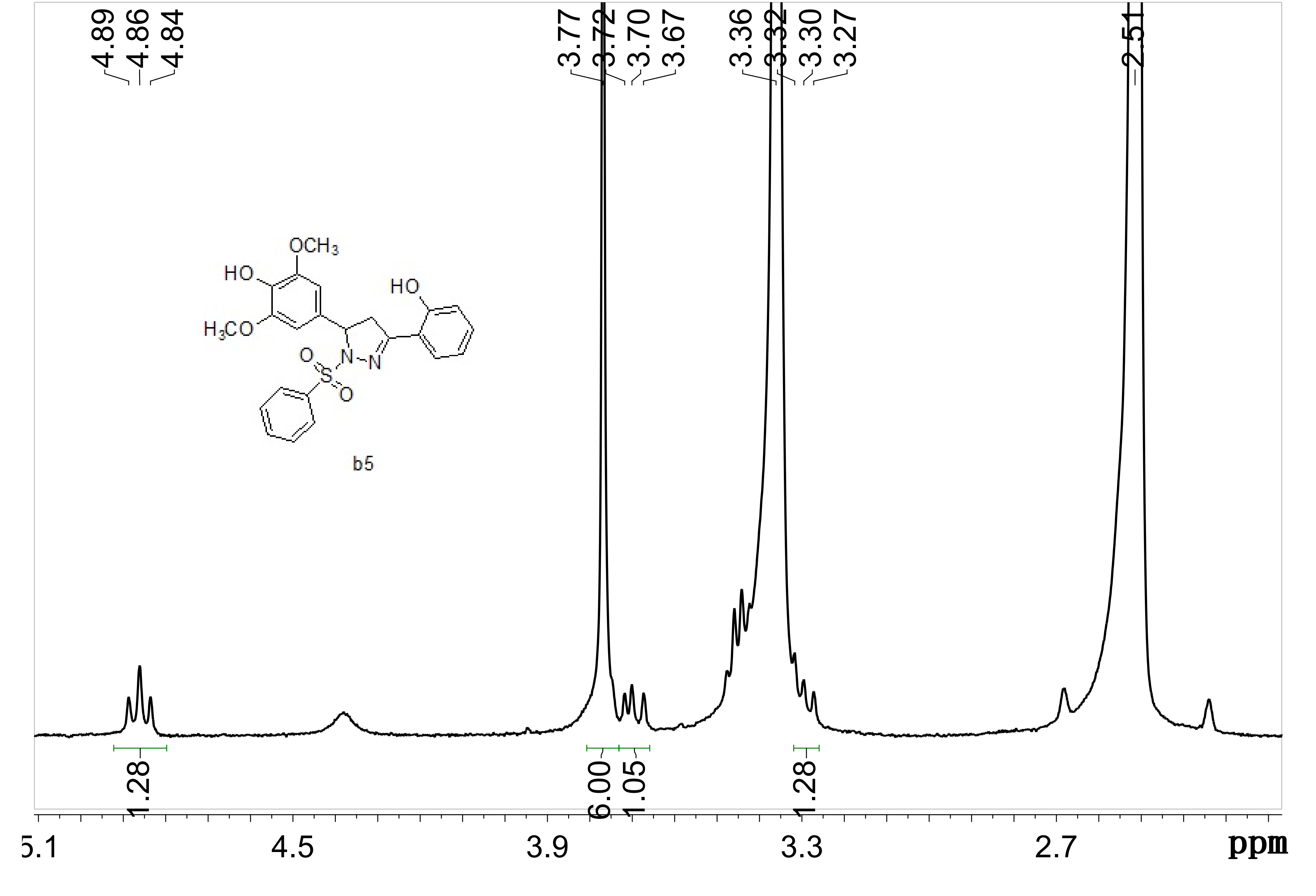
4.89
4.86
4.84
3.77
3.72
3.70
3.67
3.36
3.32
3.30
3.27
2.51
OCH3
HO
HO
H3CO
O
N
N
S
O
b5
1.28
6.00
1.05
1.28
5.1
4.5
3.9
3.3
2.7
ppm

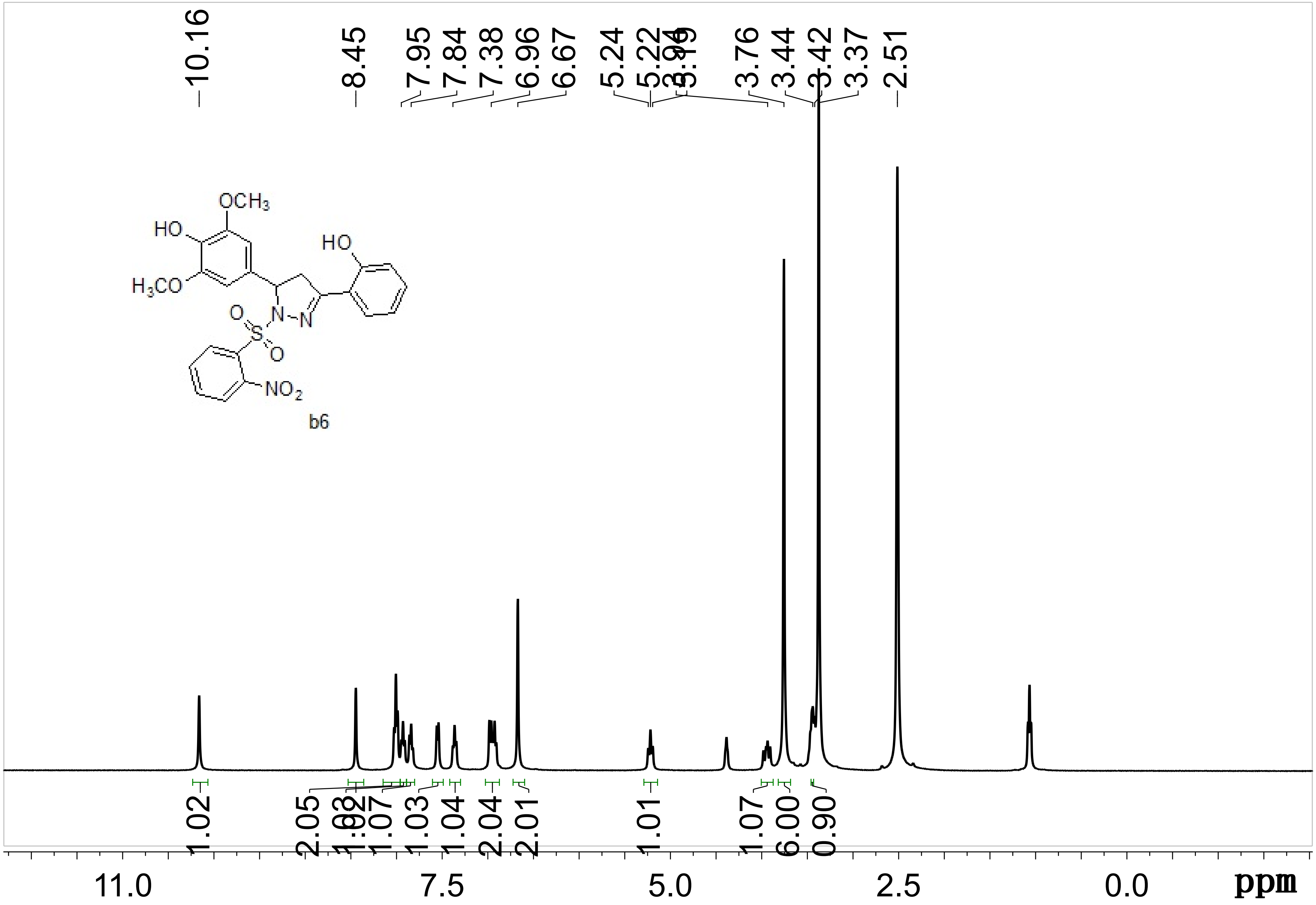

10.16
8.45
7.95
7.84
7.38
6.96
6.67
5.24
5.22
3.94
5.19
3.76
3.44
3.42
3.37
2.51
OCH3
HO
H3CO
HO
O
S
O
N
N
NO2
b6
1.02
2.05
1.03
1.92
1.07
1.03
1.04
2.04
2.01
1.01
1.07
6.00
0.90
11.0
7.5
5.0
2.5
0.0
ppm

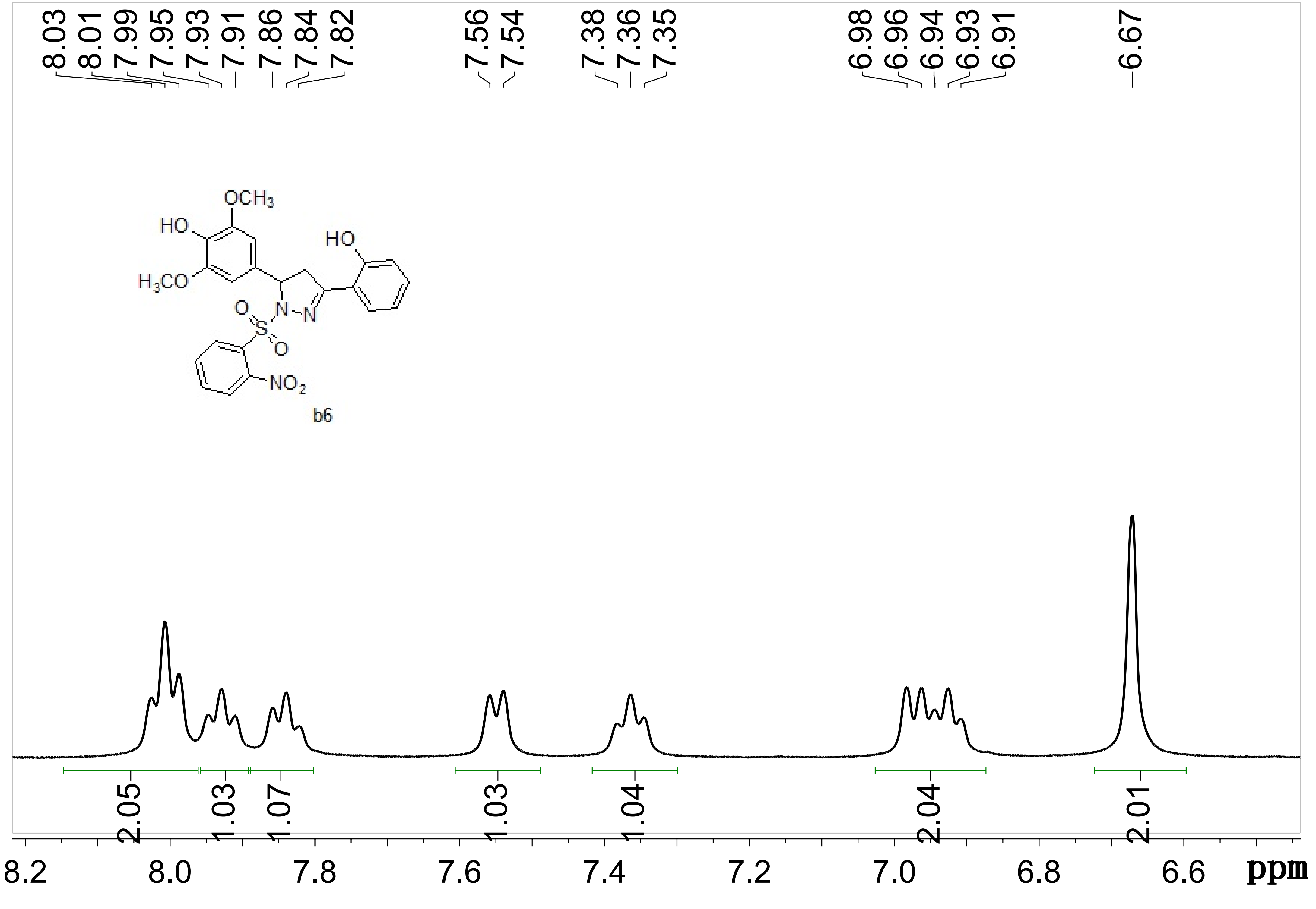

8.03
8.01
7.99
7.95
7.93
7.91
7.86
7.84
7.82
7.56
7.54
7.38
7.36
7.35
6.98
6.96
6.94
6.93
6.91
6.67
OCH3
HO
H3CO
HO
O
S
O
N
N
NO2
b6
2.05
1.03
1.07
1.03
1.04
2.04
2.01
8.2
8.0
7.8
7.6
7.4
7.2
7.0
6.8
6.6
ppm

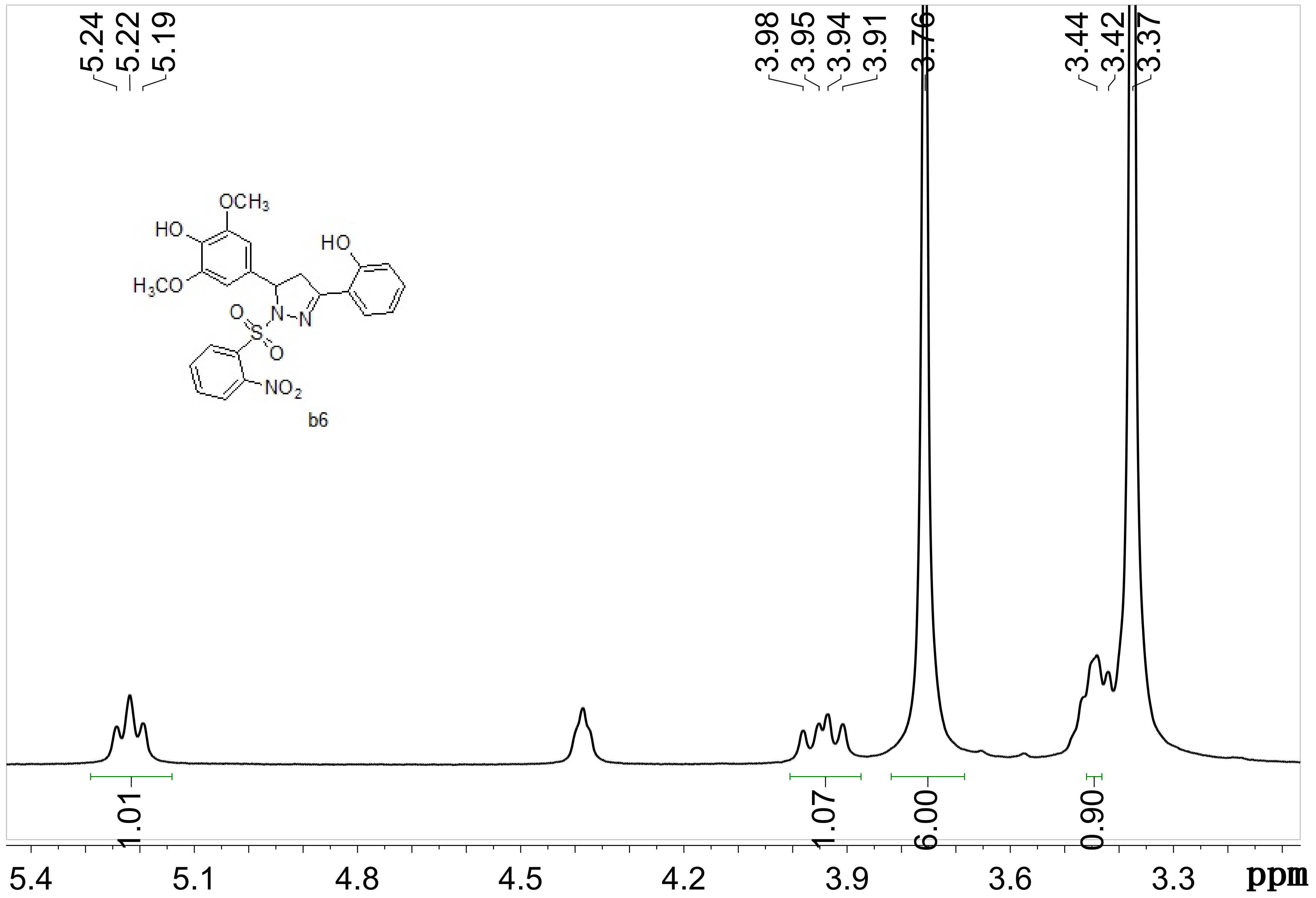

5.24
5.22
5.19
3.98
3.95
3.94
3.91
3.76
3.44
3.42
3.37
OCH3
HO
HO
H3CO
O
N
N
S
O
NO2
b6
1.01
1.07
6.00
0.90
5.4
5.1
4.8
4.5
4.2
3.9
3.6
3.3
ppm

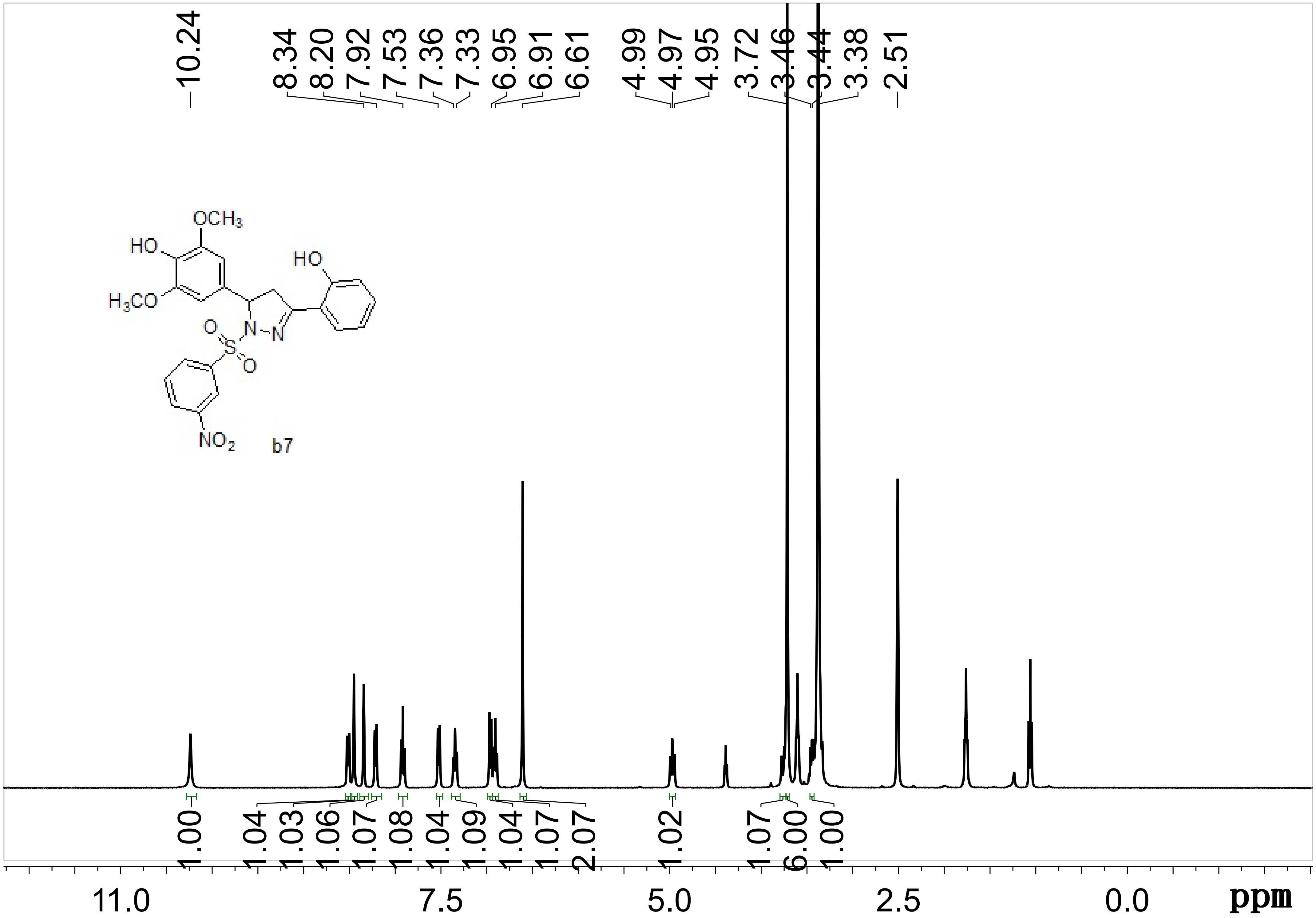
10.24
8.34
8.20
7.92
7.53
7.36
7.33
6.95
6.91
6.61
4.99
4.97
4.95
3.72
3.46
3.44
3.38
2.51
OCH3
HO
H3CO
HO
N-N
O
S
O
NO2
b7
1.00
1.04
1.03
1.06
1.07
1.08
1.04
1.09
1.04
1.07
2.07
1.02
1.07
6.00
1.00
11.0
7.5
5.0
2.5
0.0
ppm

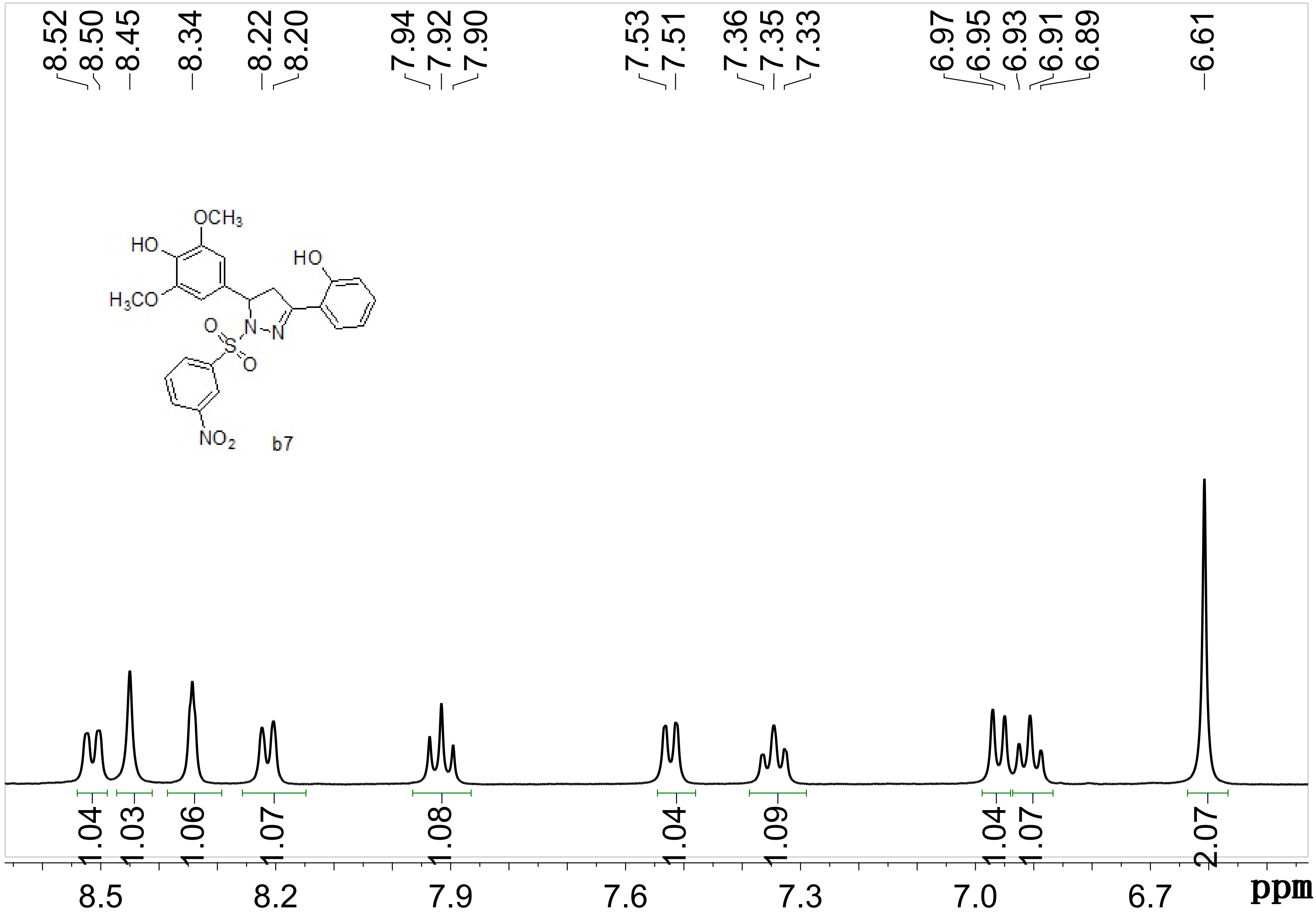

8.52
8.50
8.45
8.34
8.22
8.20
7.94
7.92
7.90
7.53
7.51
7.36
7.35
7.33
6.97
6.95
6.93
6.91
6.89
6.61
OCH3
HO
H3CO
HO
N
N
O
S
O
NO2
b7
1.04
1.03
1.06
1.07
1.08
1.04
1.09
1.04
1.07
2.07
8.5
8.2
7.9
7.6
7.3
7.0
6.7
ppm

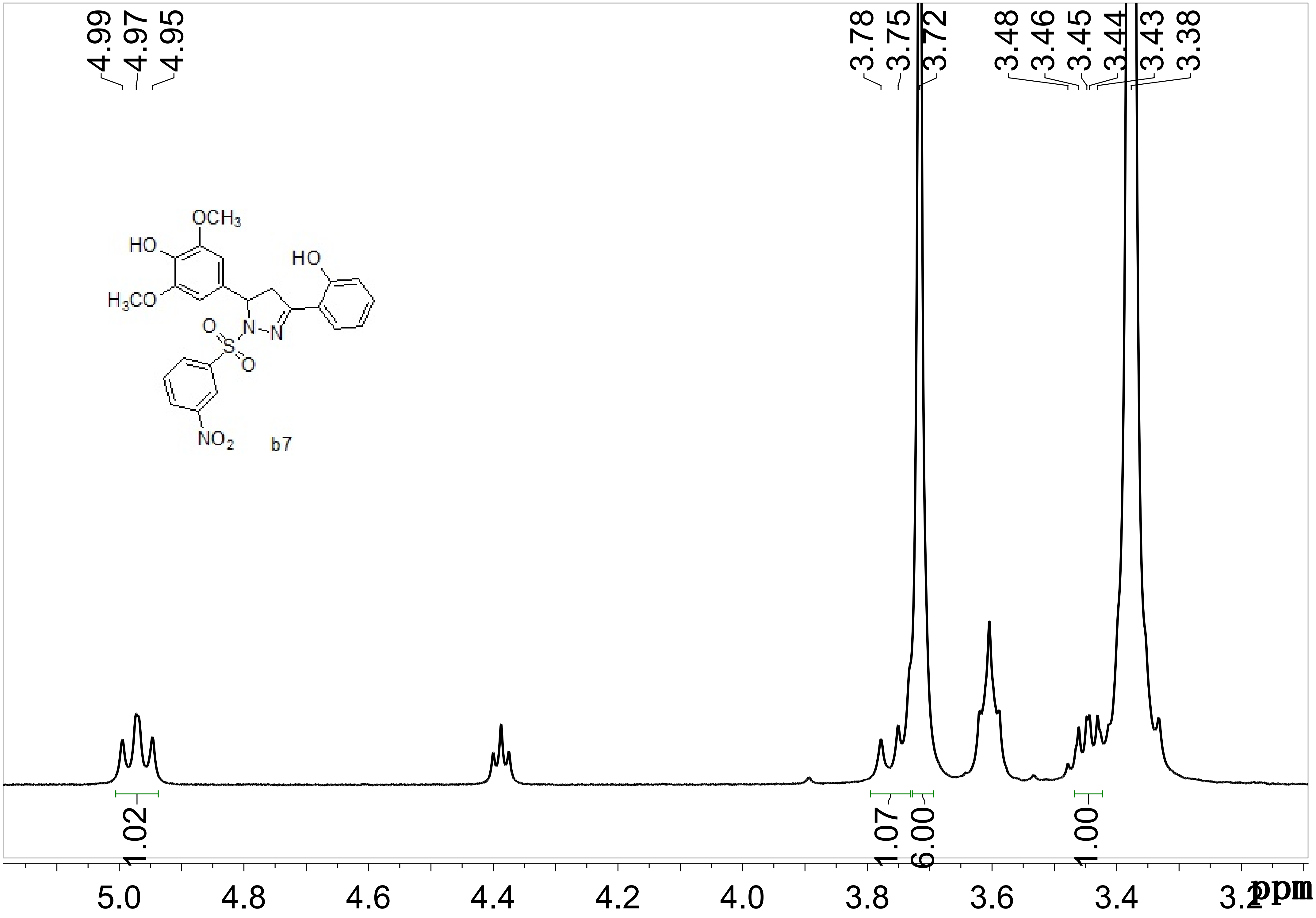

4.99
4.97
4.95
3.78
3.75
3.72
3.48
3.46
3.45
3.44
3.43
3.38
OCH3
HO
H3CO
HO
N
N
O
S
O
NO2
b7
1.02
1.07
6.00
1.00
5.0
4.8
4.6
4.4
4.2
4.0
3.8
3.6
3.4
3.2
ppm

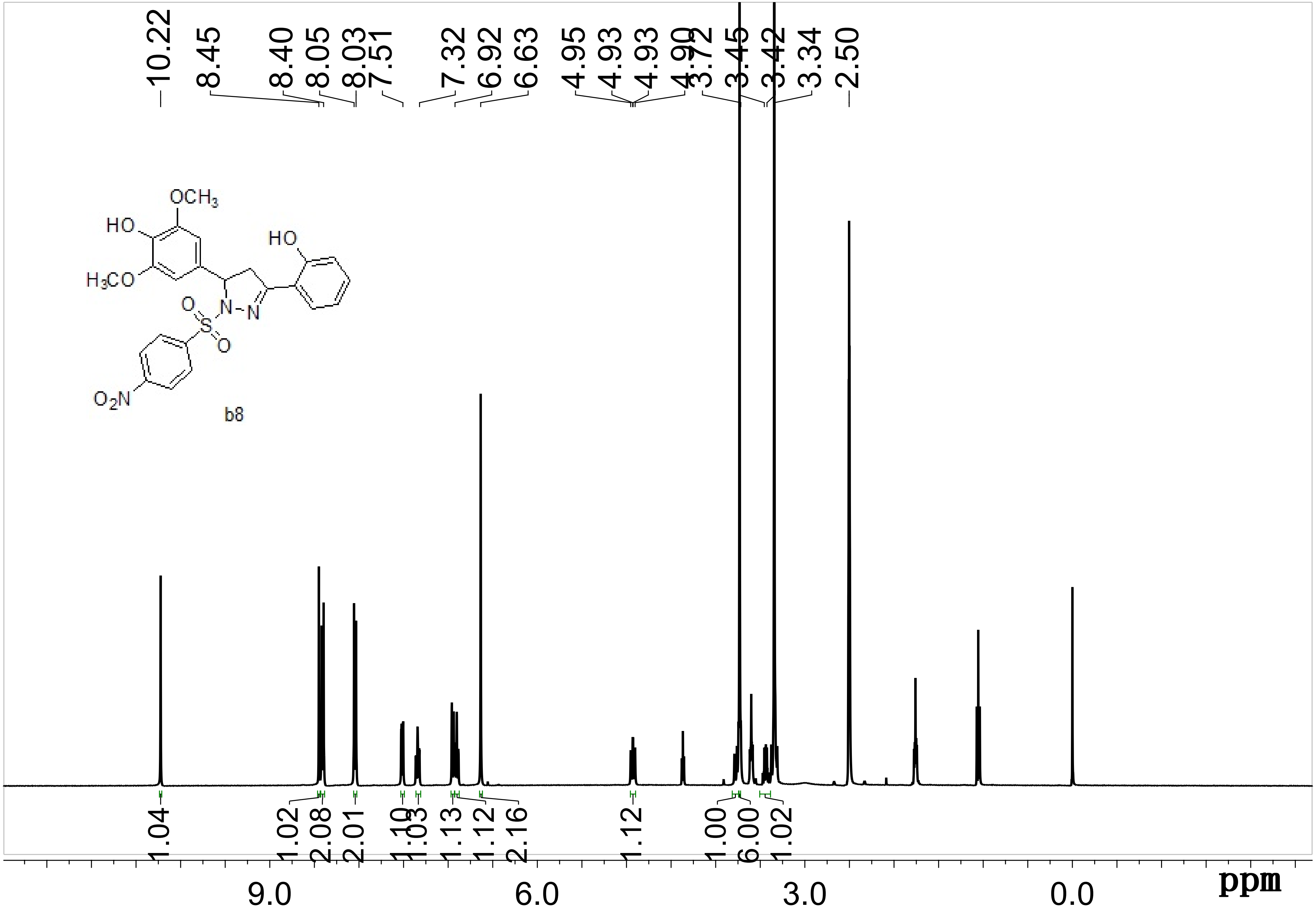
10.22
8.45
8.40
8.05
8.03
7.51
7.32
6.92
6.63
4.95
4.93
4.93
4.90
3.72
3.45
3.42
3.34
2.50
OCH3
HO
HO
H3CO
O
S
O
N
N
O2N
b8
1.04
1.02
2.08
2.01
1.10
1.03
1.13
1.12
2.16
1.12
1.00
6.00
1.02
9.0
6.0
3.0
0.0
ppm

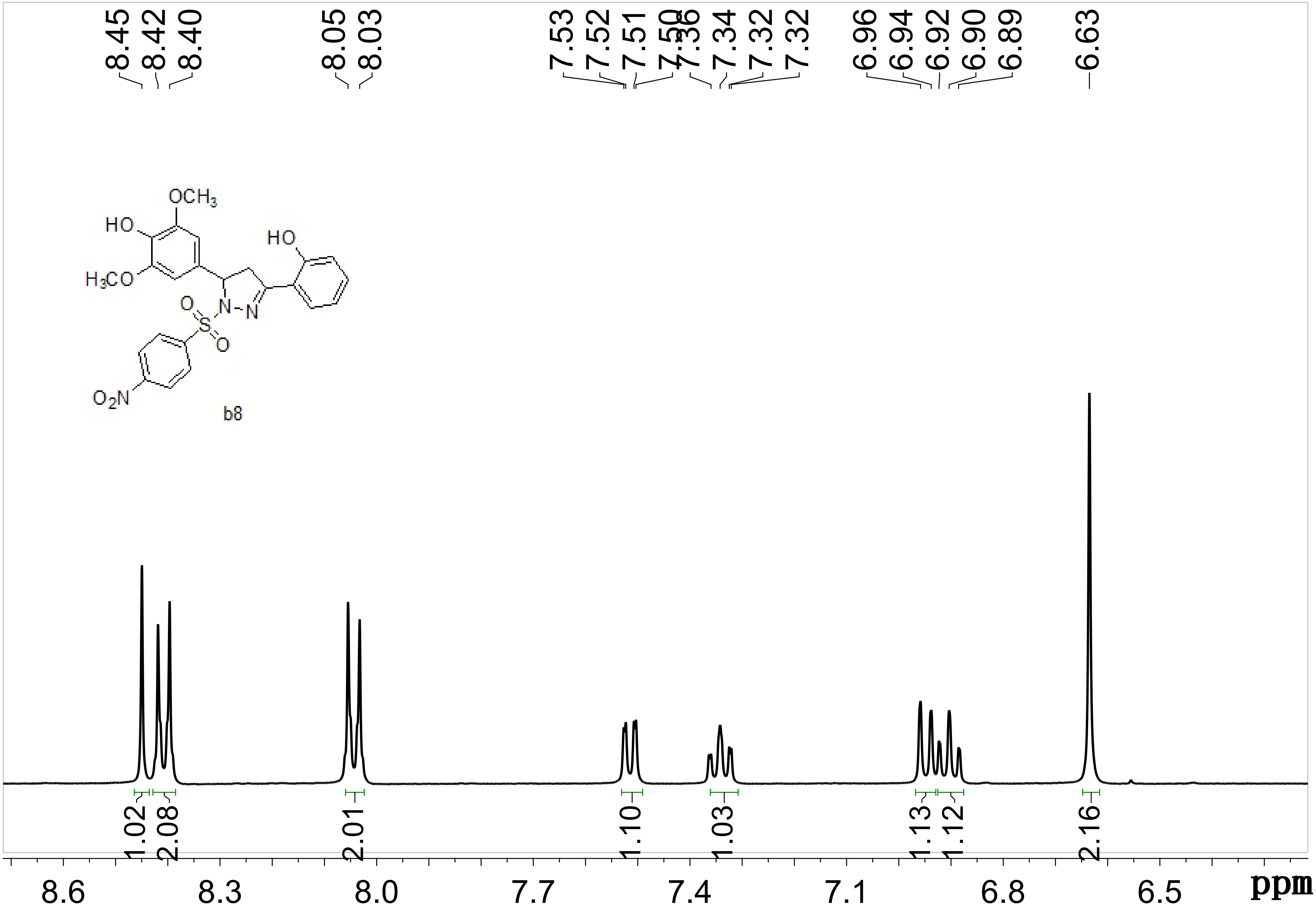
8.45
8.42
8.40
8.05
8.03
7.53
7.52
7.51
7.50
7.36
7.34
7.32
7.32
6.96
6.94
6.92
6.90
6.89
6.63
OCH3
HO
HO
H3CO
O
N-N
S
O
O2N
b8
1.02
2.08
2.01
1.10
1.03
1.13
1.12
2.16
8.6
8.3
8.0
7.7
7.4
7.1
6.8
6.5
ppm

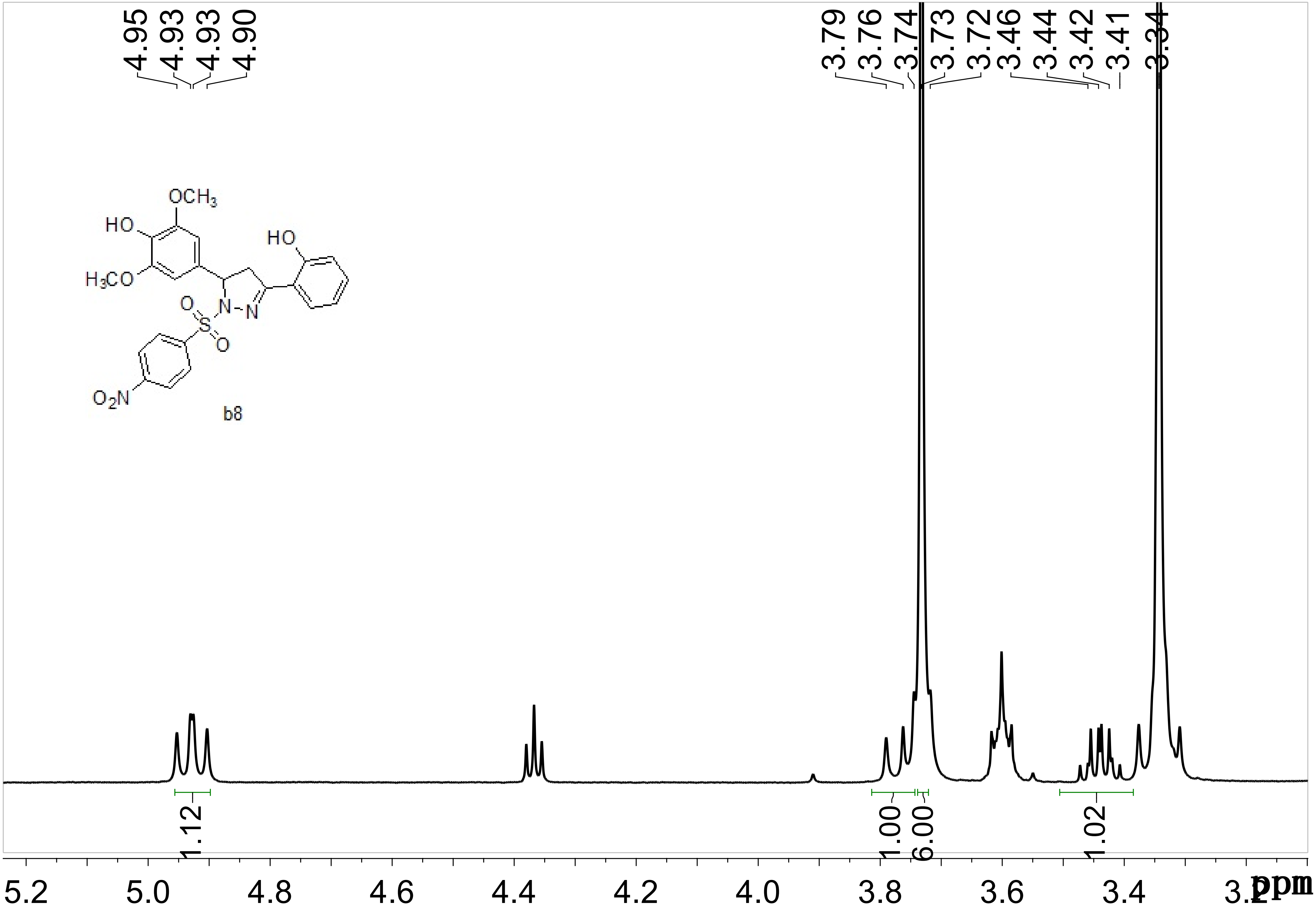
4.95
4.93
4.93
4.90
3.79
3.76
3.74
3.73
3.72
3.46
3.44
3.42
3.41
3.34
OCH3
HO
H3CO
HO
O
S
O
N
N
O2N
b8
1.12
1.00
6.00
1.02
5.2
5.0
4.8
4.6
4.4
4.2
4.0
3.8
3.6
3.4
3.2
ppm

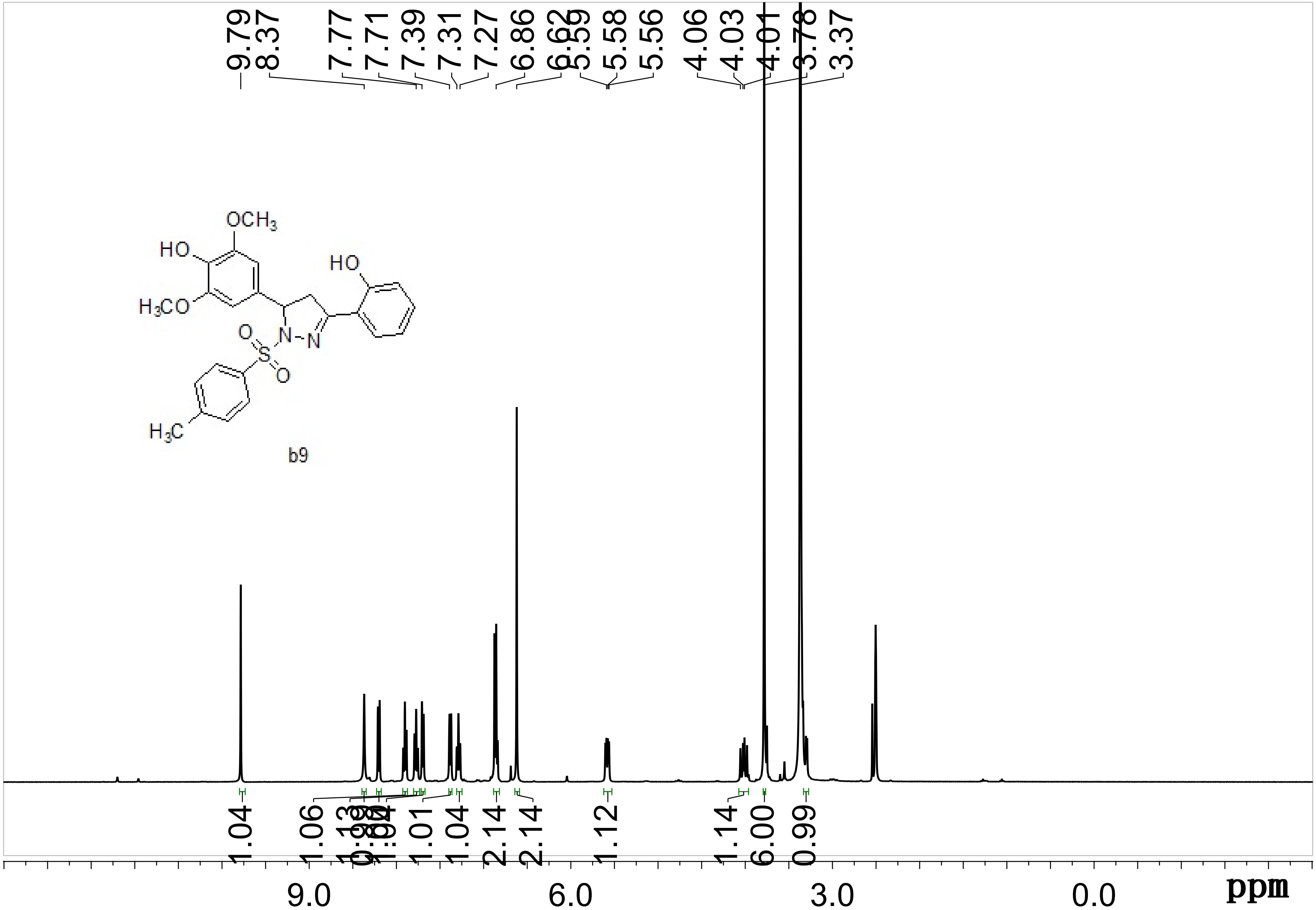

9.79
8.37
7.77
7.71
7.39
7.31
7.27
6.86
6.62
5.59
5.58
5.56
4.06
4.03
4.01
3.78
3.37
OCH3
HO
HO
H3CO
O
S
O
N
N
H3C
b9
1.04
1.06
1.01
1.04
2.14
2.14
1.12
1.14
6.00
0.99
9.0
6.0
3.0
0.0
ppm

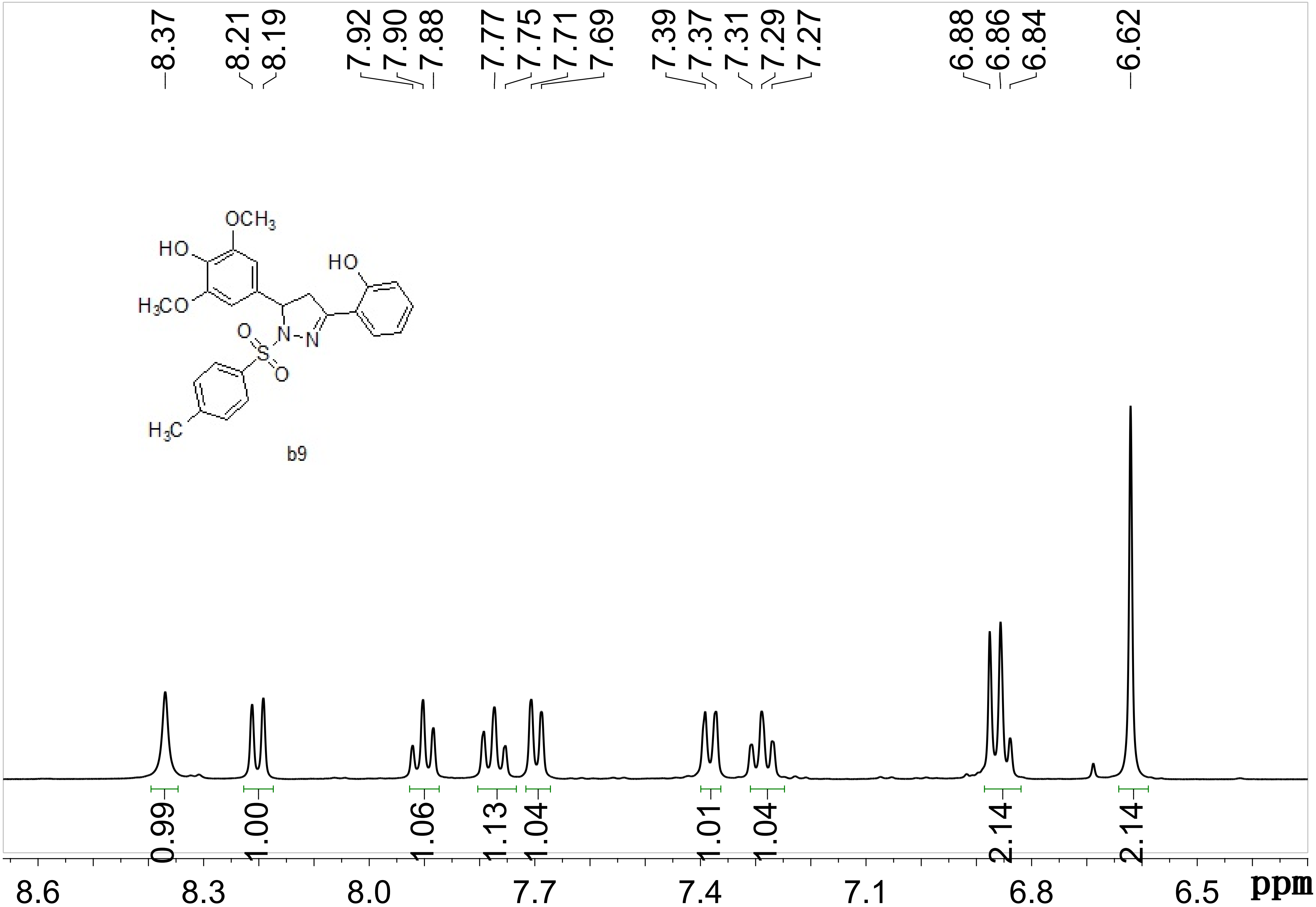
8.37
8.21
8.19
7.92
7.90
7.88
7.77
7.75
7.71
7.69
7.39
7.37
7.31
7.29
7.27
6.88
6.86
6.84
6.62
OCH3
HO
H3CO
HO
N-N
O
S
O
H3C
b9
0.99
1.00
1.06
1.13
1.04
1.01
1.04
2.14
2.14
8.6
8.3
8.0
7.7
7.4
7.1
6.8
6.5
ppm

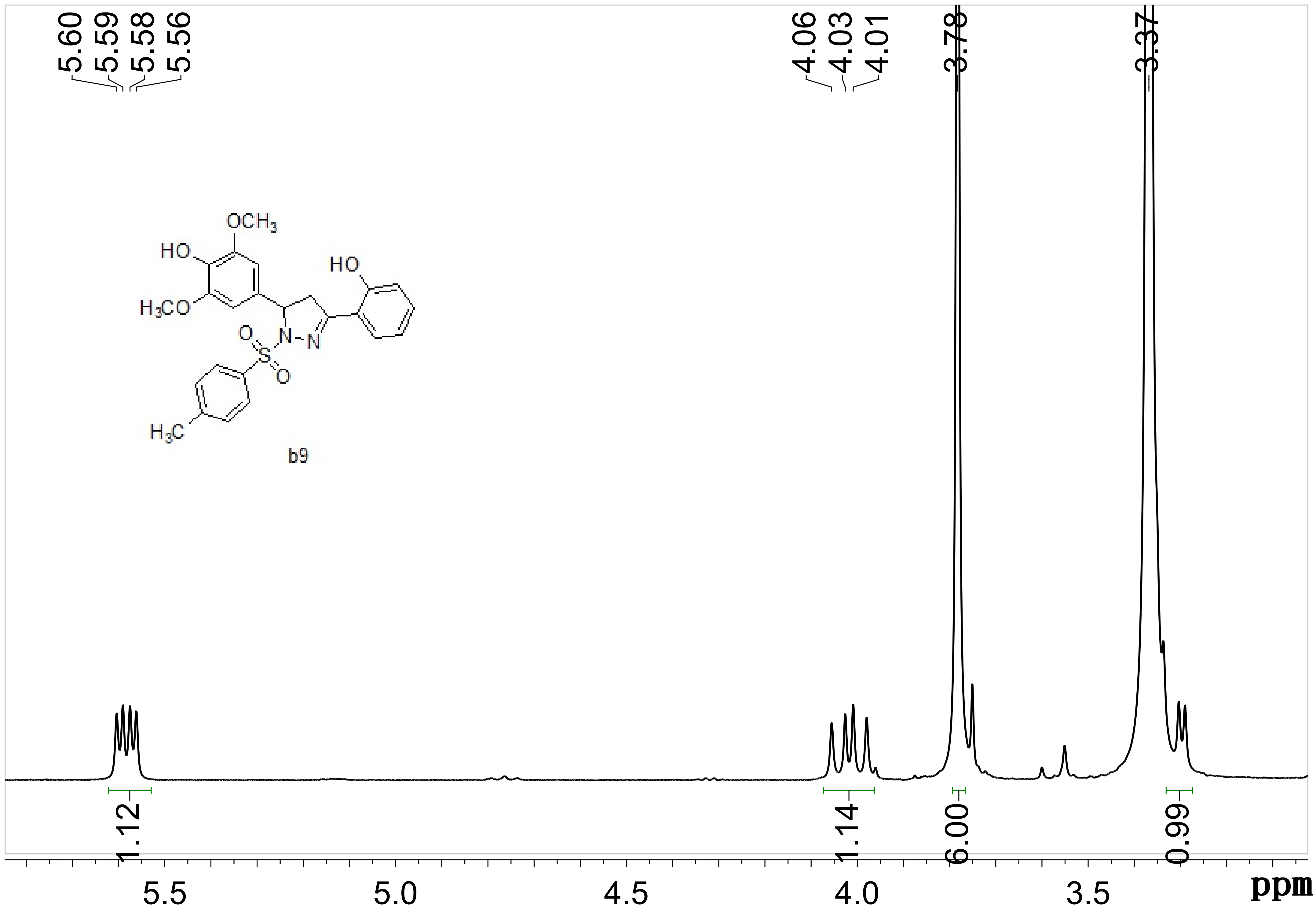
5.60
5.59
5.58
5.56
4.06
4.03
4.01
3.78
3.37
OCH3
HO
H3CO
HO
N
N
S
O
O
H3C
b9
1.12
1.14
6.00
0.99
5.5
5.0
4.5
4.0
3.5
ppm

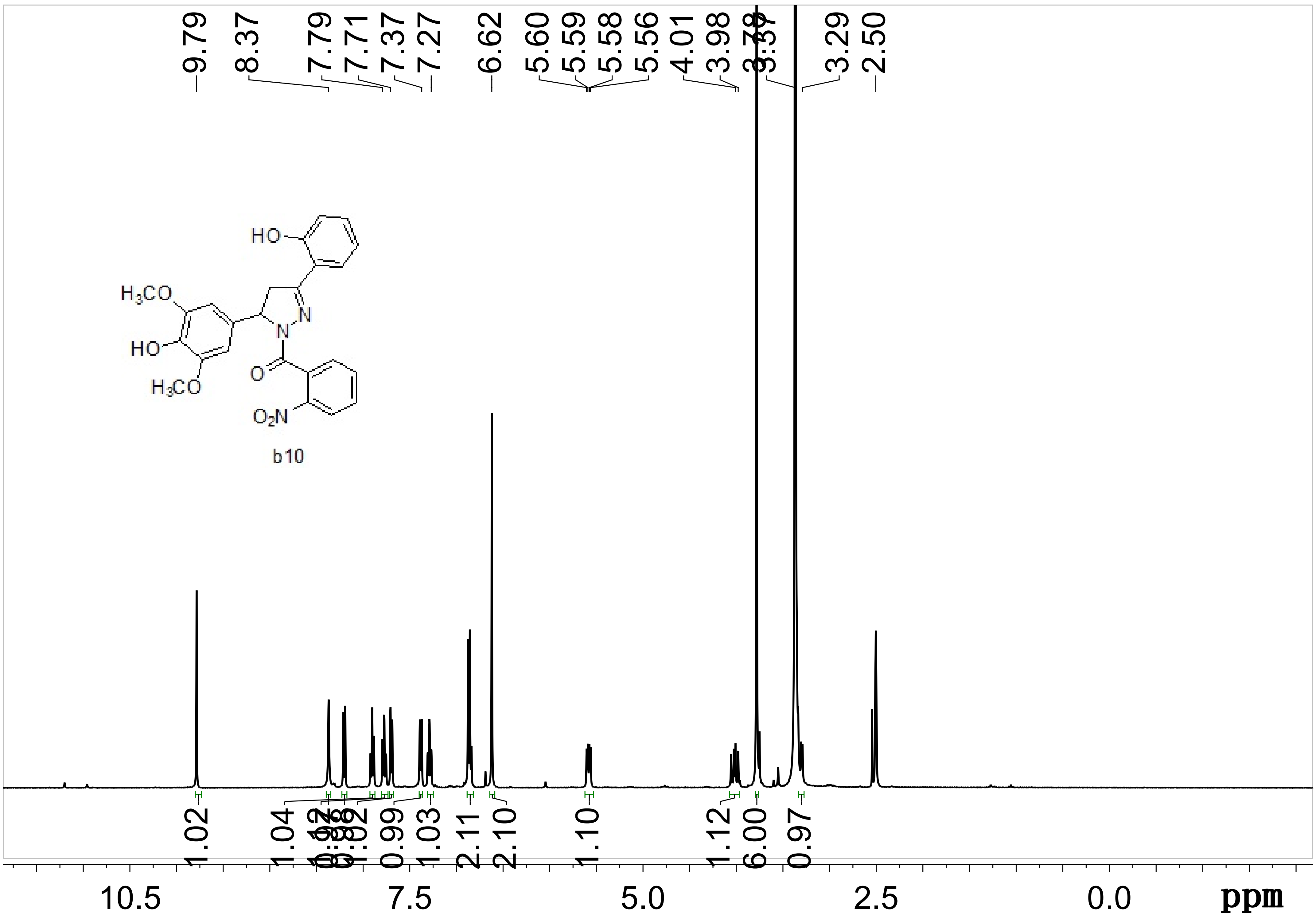
9.79
8.37
7.79
7.71
7.37
7.27
6.62
5.60
5.59
5.58
5.56
4.01
3.98
3.29
2.50
HO
H3CO
HO
H3CO
N
N
O
O2N
b10
1.02
1.04
0.99
1.03
2.11
2.10
1.10
1.12
6.00
0.97
10.5
7.5
5.0
2.5
0.0
ppm

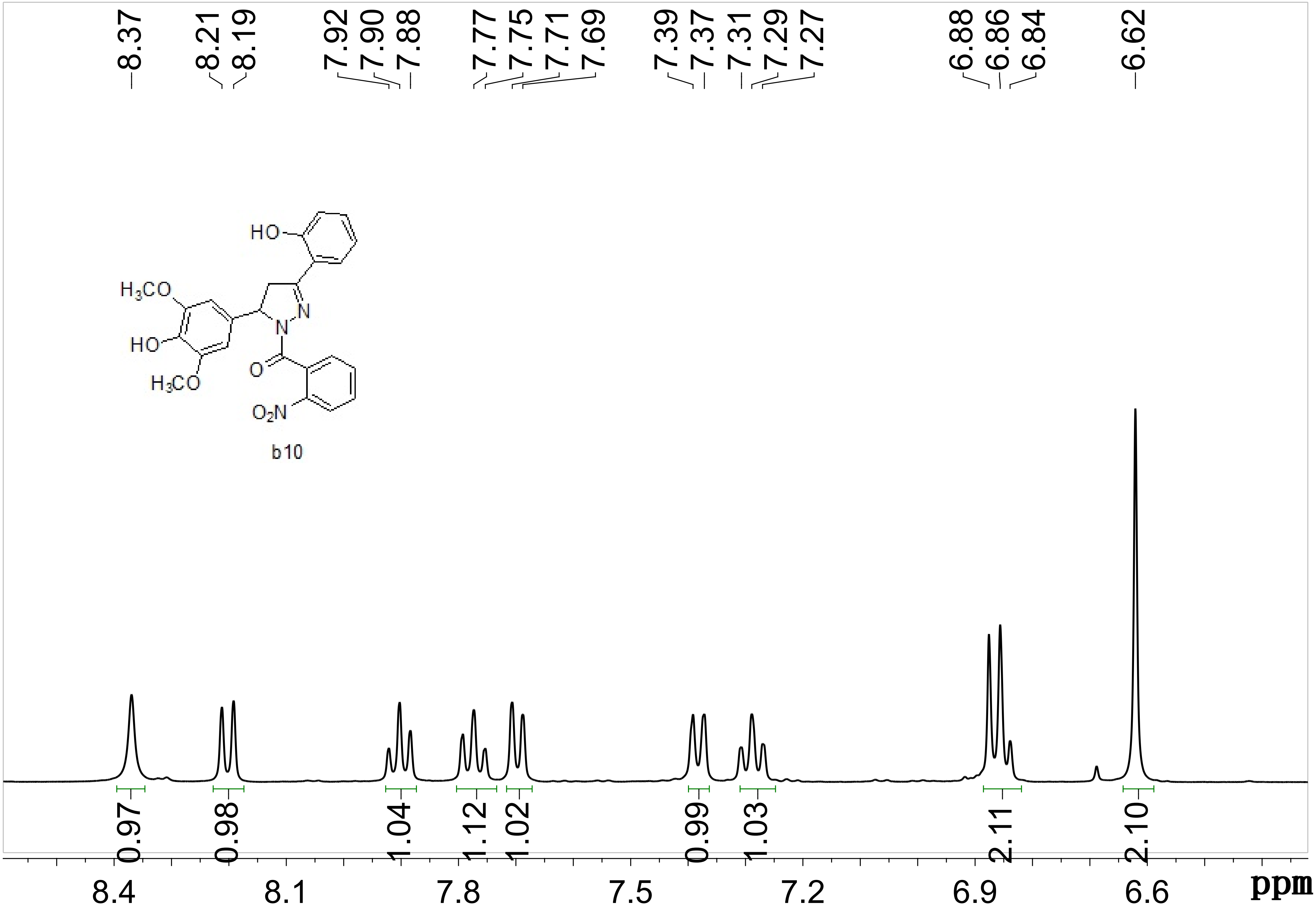
8.37
8.21
8.19
7.92
7.90
7.88
7.77
7.75
7.71
7.69
7.39
7.37
7.31
7.29
7.27
6.88
6.86
6.84
6.62
HO
H3CO
HO
H3CO
N
N
O
O2N
b10
0.97
0.98
1.04
1.12
1.02
0.99
1.03
2.11
2.10
8.4
8.1
7.8
7.5
7.2
6.9
6.6
ppm

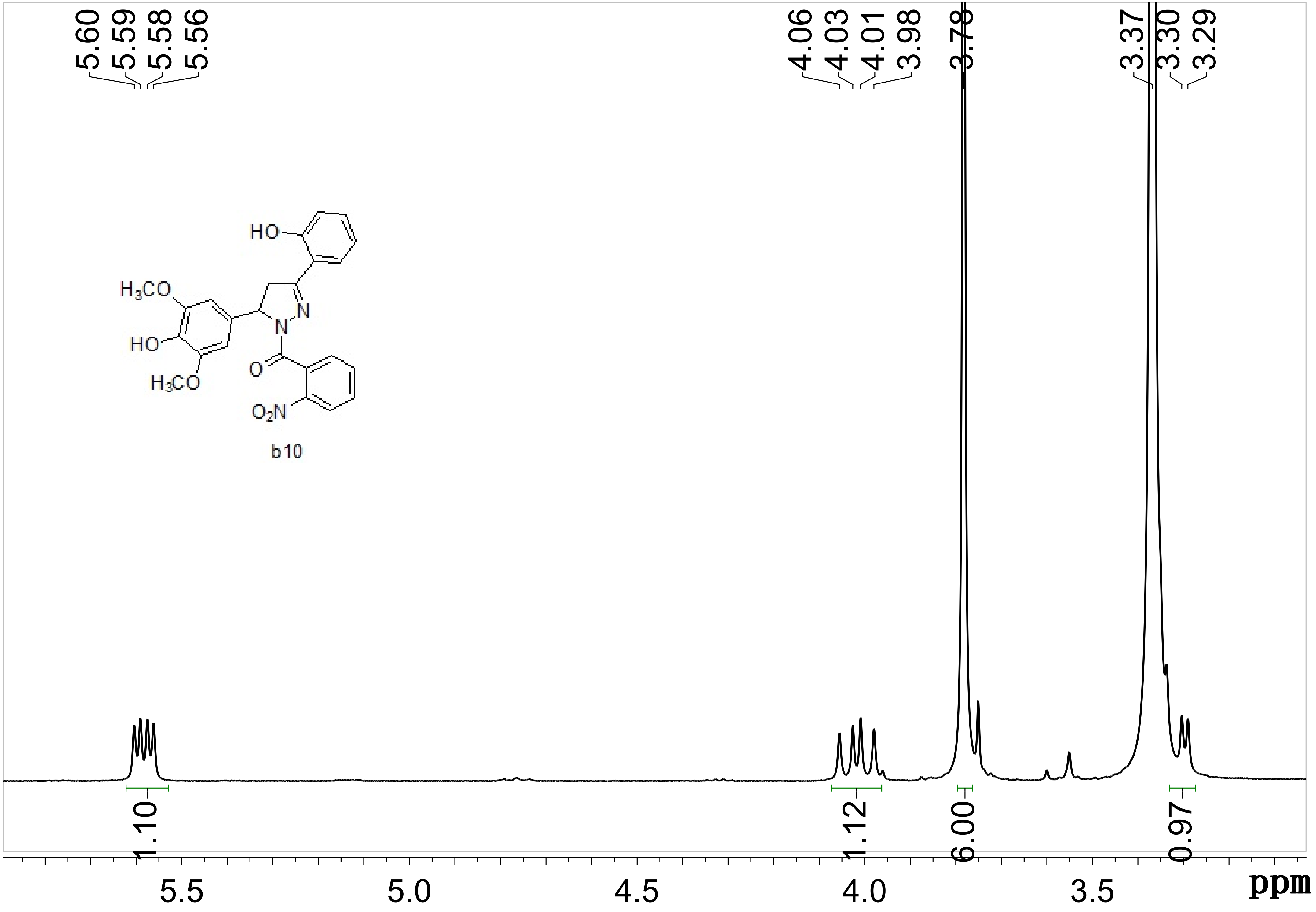

5.60
5.59
5.58
5.56
4.06
4.03
4.01
3.98
3.78
3.37
3.30
3.29
HO
H3CO
HO
H3CO
N
N
O
O2N
b10
1.10
1.12
6.00
0.97
5.5
5.0
4.5
4.0
3.5
ppm

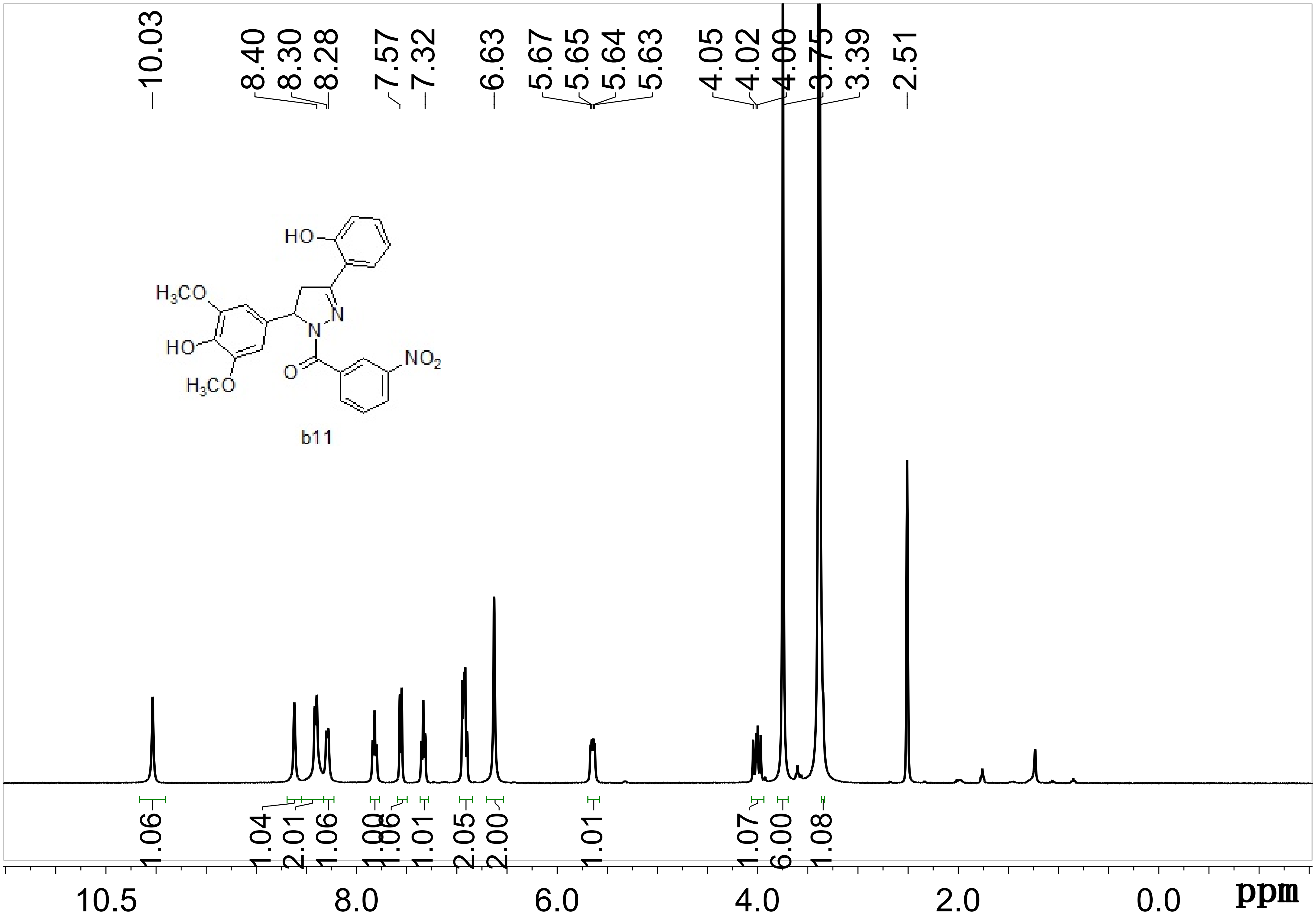
10.03
8.40
8.30
8.28
7.57
7.32
6.63
5.67
5.65
5.64
5.63
4.05
4.02
4.00
3.75
3.39
2.51
HO
H3CO
HO
H3CO
N
N
O
NO2
b11
1.06
1.04
2.01
1.06
1.00
1.06
1.01
2.05
2.00
1.01
1.07
6.00
1.08
10.5
8.0
6.0
4.0
2.0
0.0
ppm

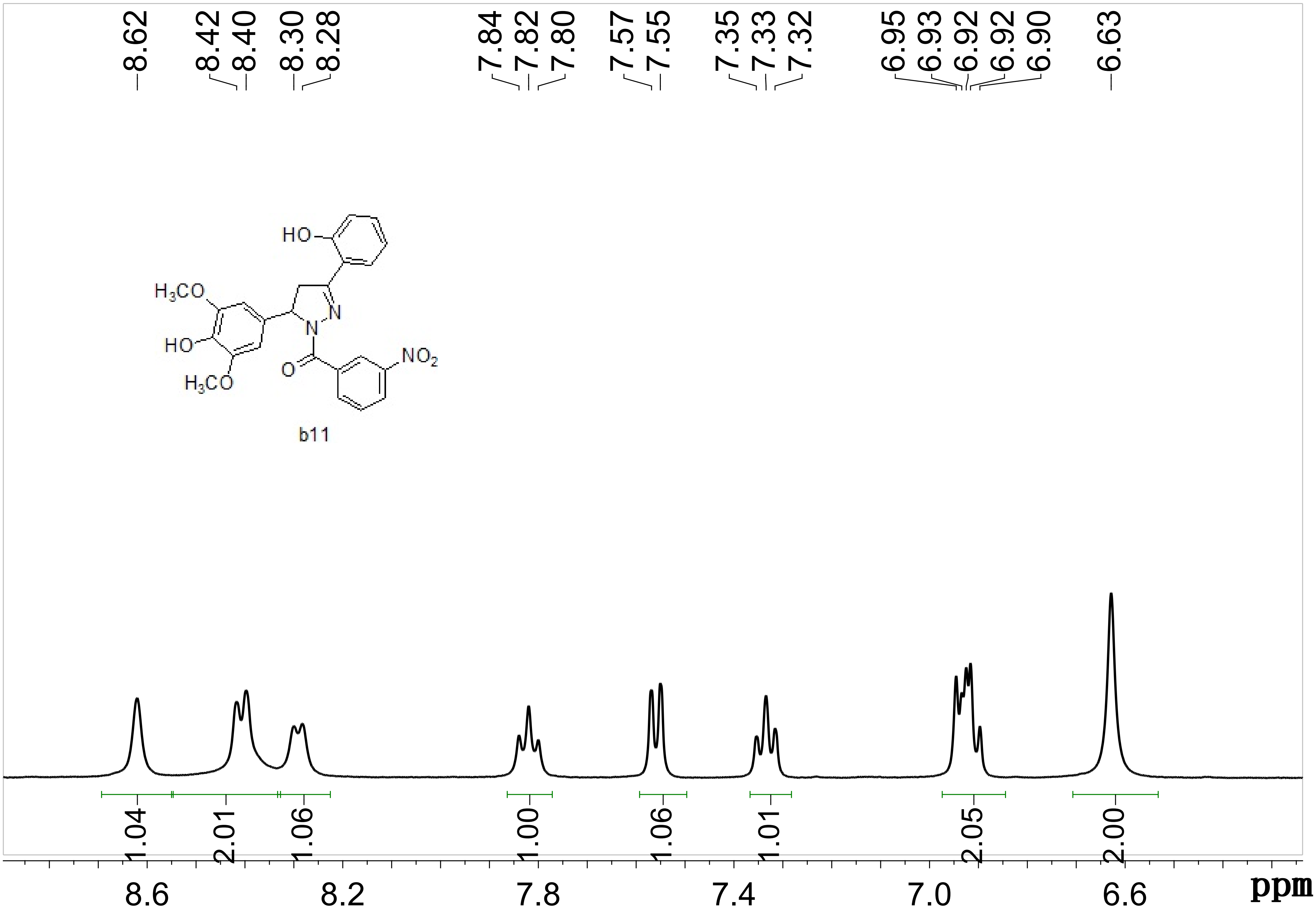
8.62
8.42
8.40
8.30
8.28
7.84
7.82
7.80
7.57
7.55
7.35
7.33
7.32
6.95
6.93
6.92
6.92
6.90
6.63
HO
H_3CO
HO
H_3CO
N
N
O
NO_2
b11
1.04
2.01
1.06
1.00
1.06
1.01
2.05
2.00
8.6
8.2
7.8
7.4
7.0
6.6
ppm

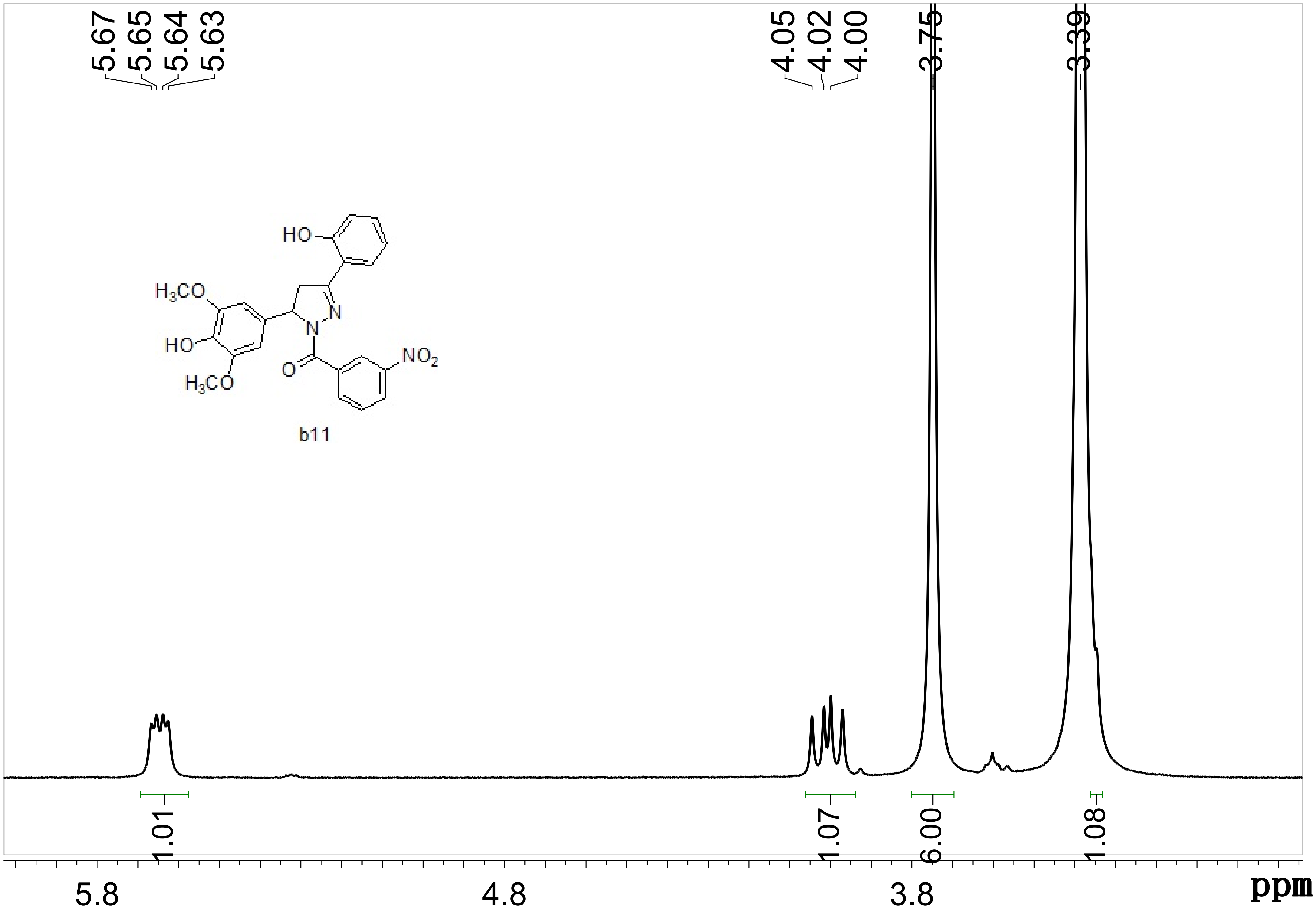

5.67
5.65
5.64
5.63
4.05
4.02
4.00
3.75
3.39
HO
H_3CO
HO
H_3CO
N
N
O
NO_2
b11
1.01
1.07
6.00
1.08
5.8
4.8
3.8
ppm

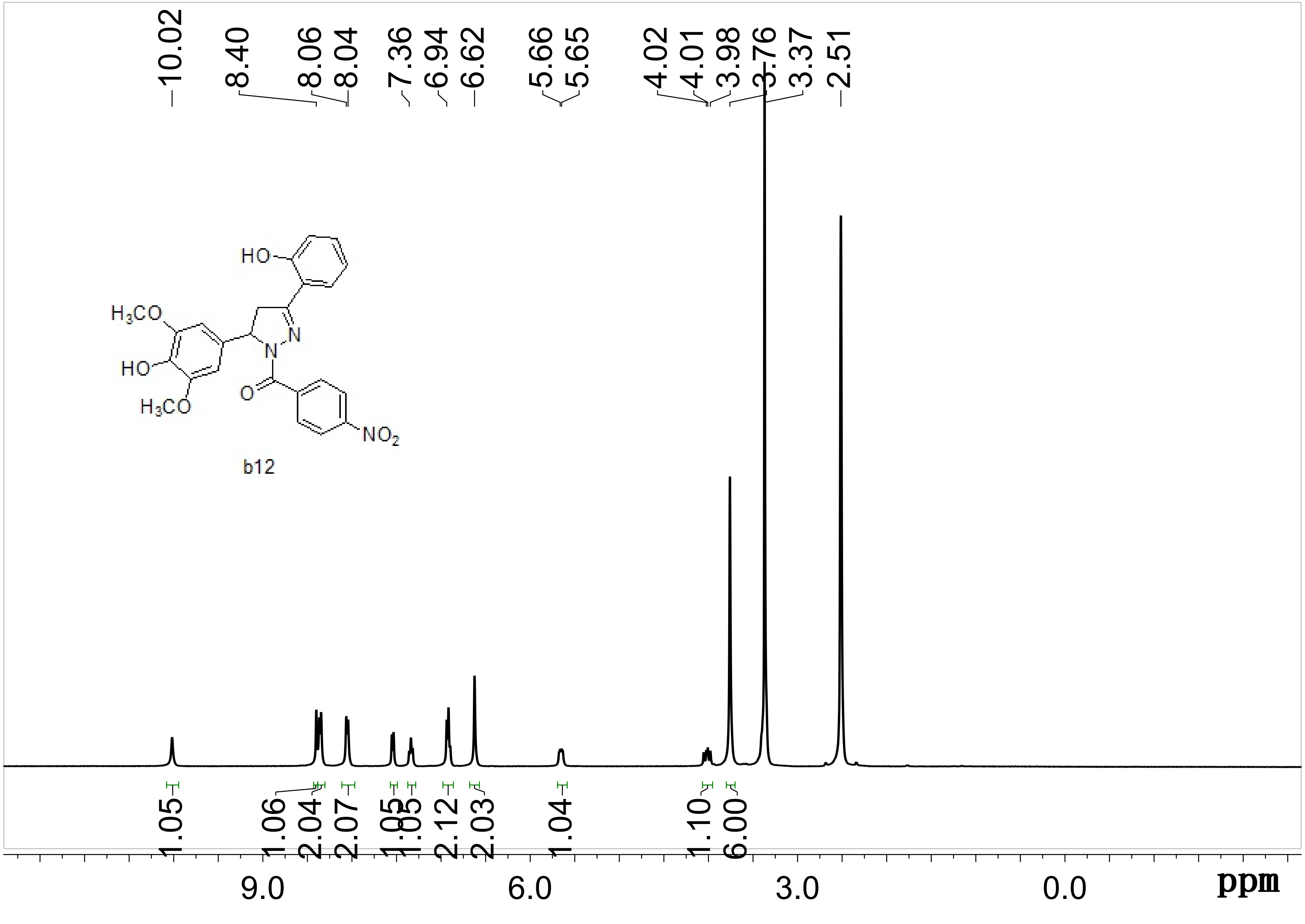

10.02
8.40
8.06
8.04
7.36
6.94
6.62
5.66
5.65
4.02
4.01
3.98
3.76
3.37
2.51
HO
H3CO
HO
H3CO
N
N
O
NO2
b12
1.05
1.06
2.04
2.07
1.05
1.05
2.12
2.03
1.04
1.10
6.00
9.0
6.0
3.0
0.0
ppm

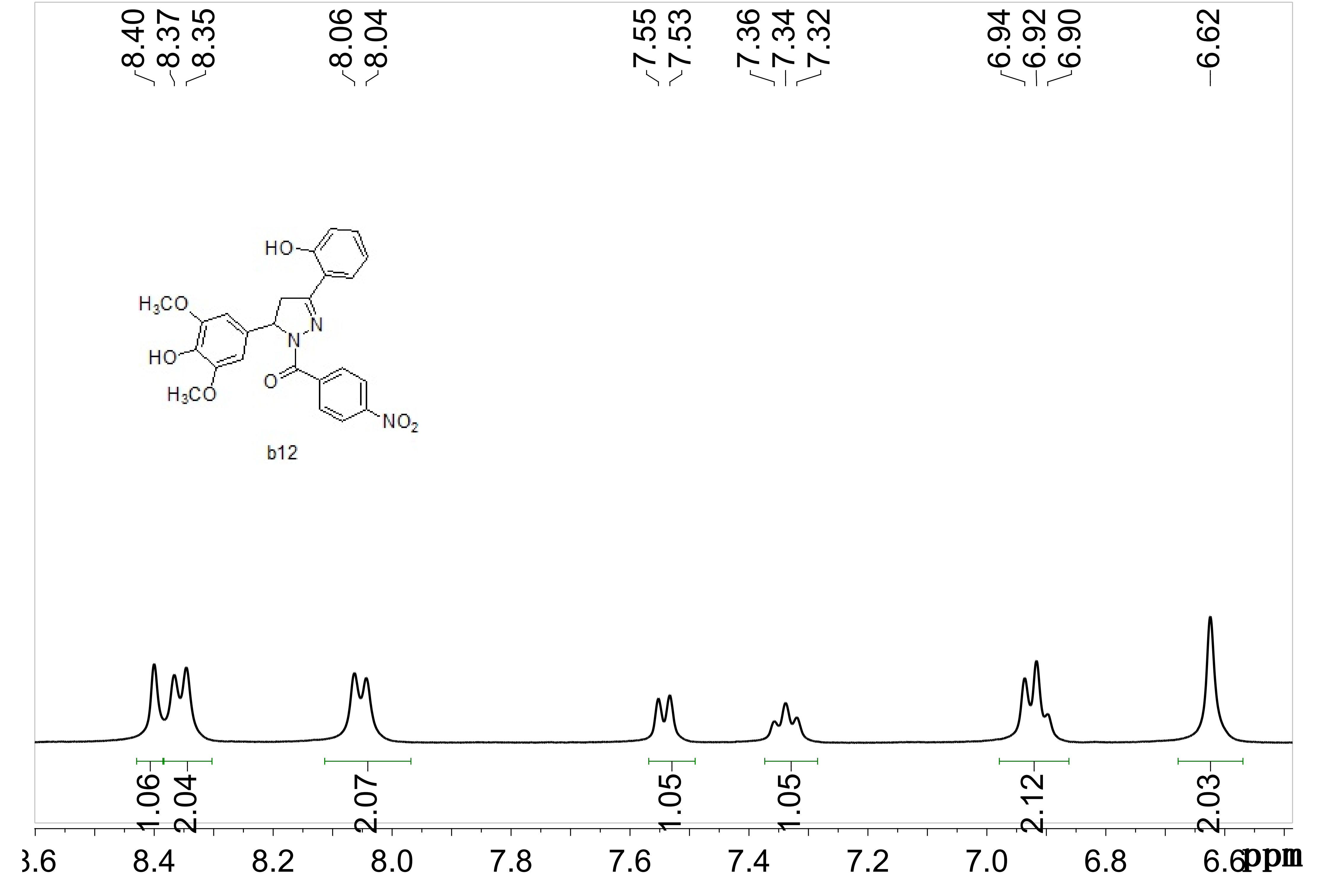

8.40
8.37
8.35
8.06
8.04
7.55
7.53
7.36
7.34
7.32
6.94
6.92
6.90
6.62
HO
H_3CO
HO
H_3CO
N
N
O
NO_2
b12
1.06
2.04
2.07
1.05
1.05
2.12
2.03
8.4
8.2
8.0
7.8
7.6
7.4
7.2
7.0
6.8
ppm

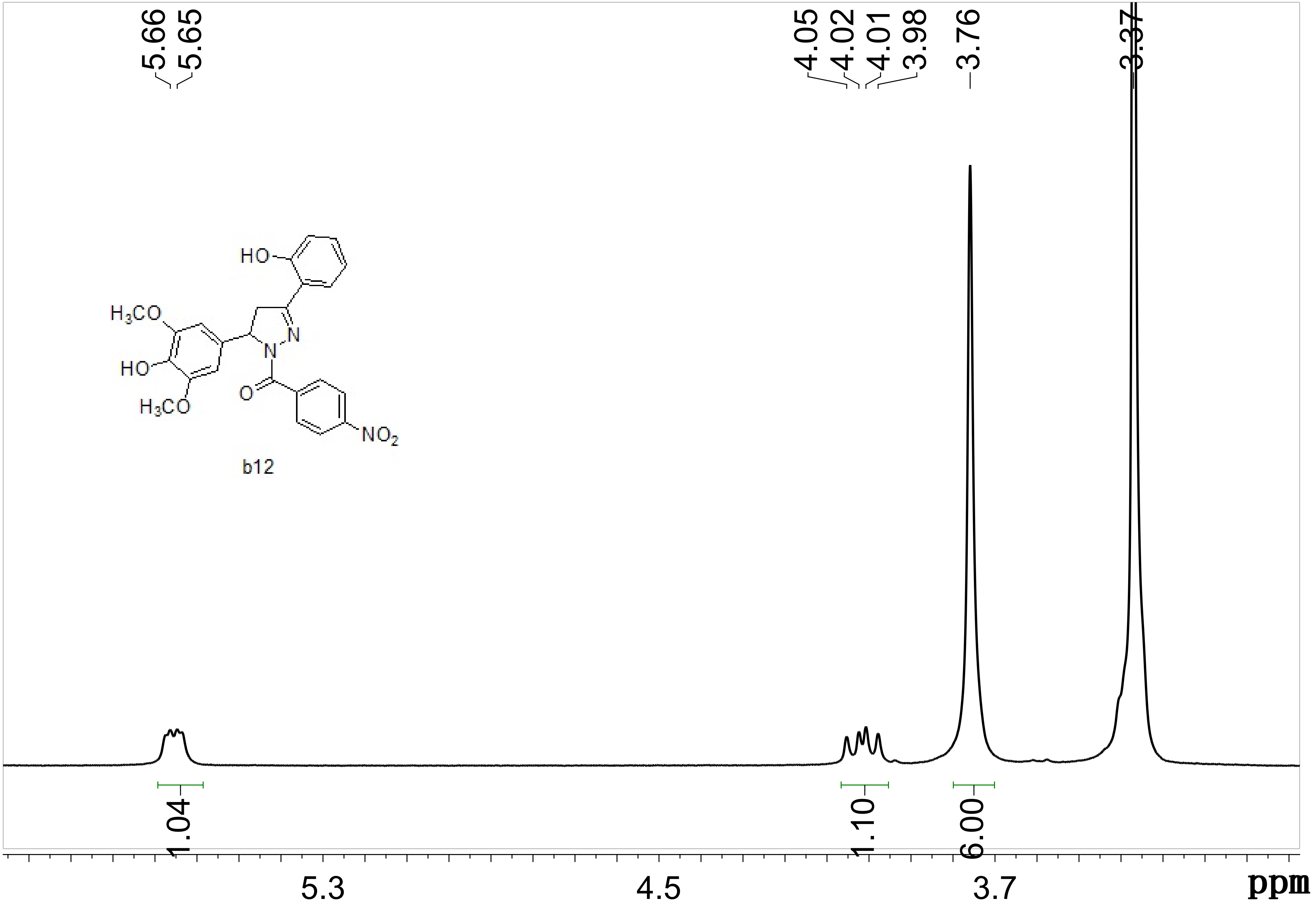

5.66
5.65
4.05
4.02
4.01
3.98
3.76
3.37
HO
H_3CO
HO
H_3CO
N
N
O
NO_2
b12
1.04
1.10
6.00
5.3
4.5
3.7
ppm

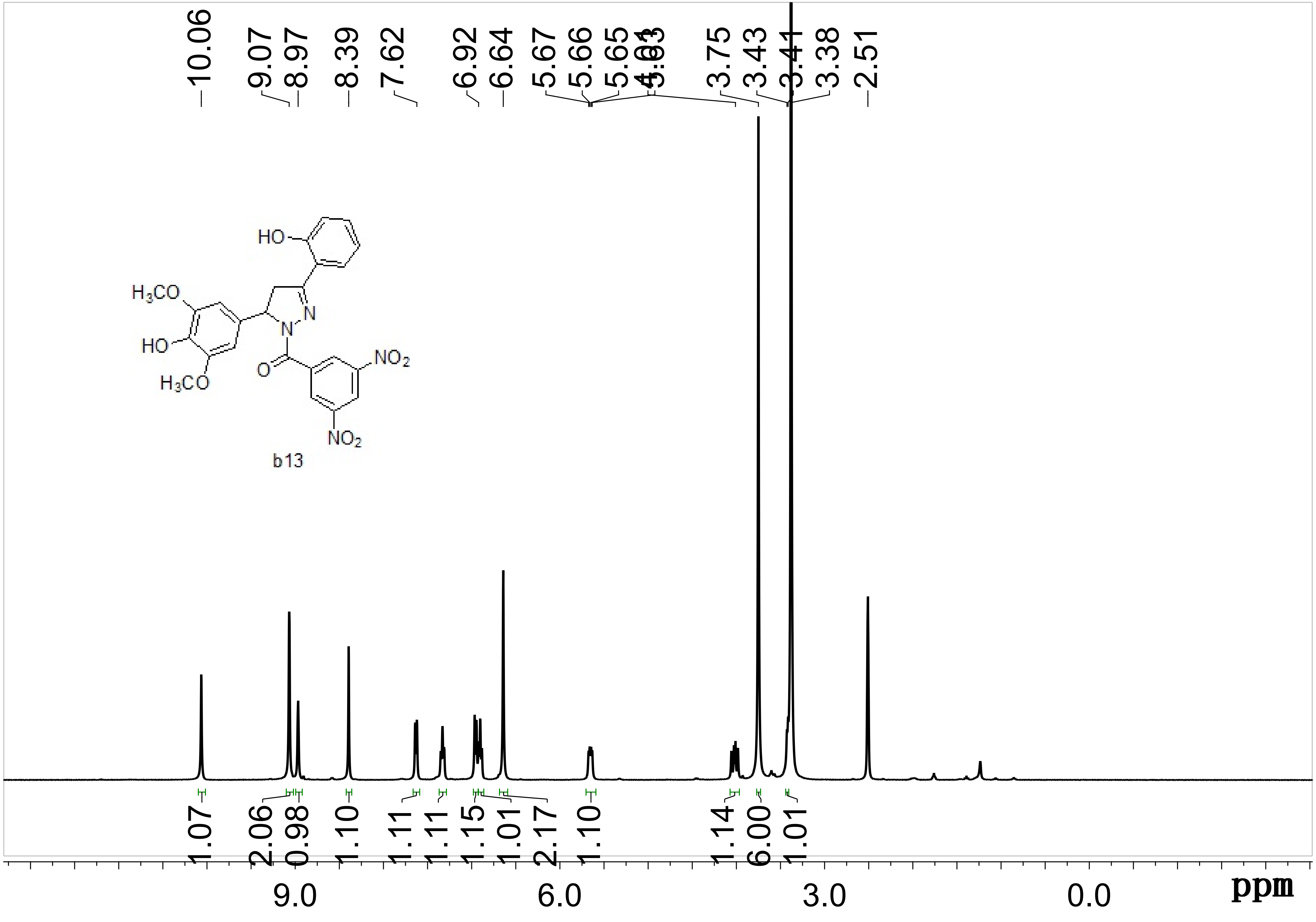
10.06
9.07
8.97
8.39
7.62
6.92
6.64
5.67
5.66
5.65
3.75
3.43
3.41
3.38
2.51
HO
H3CO
HO
H3CO
N
N
O
NO2
NO2
b13
1.07
2.06
0.98
1.10
1.11
1.11
1.15
1.01
2.17
1.10
1.14
6.00
1.01
9.0
6.0
3.0
0.0
ppm

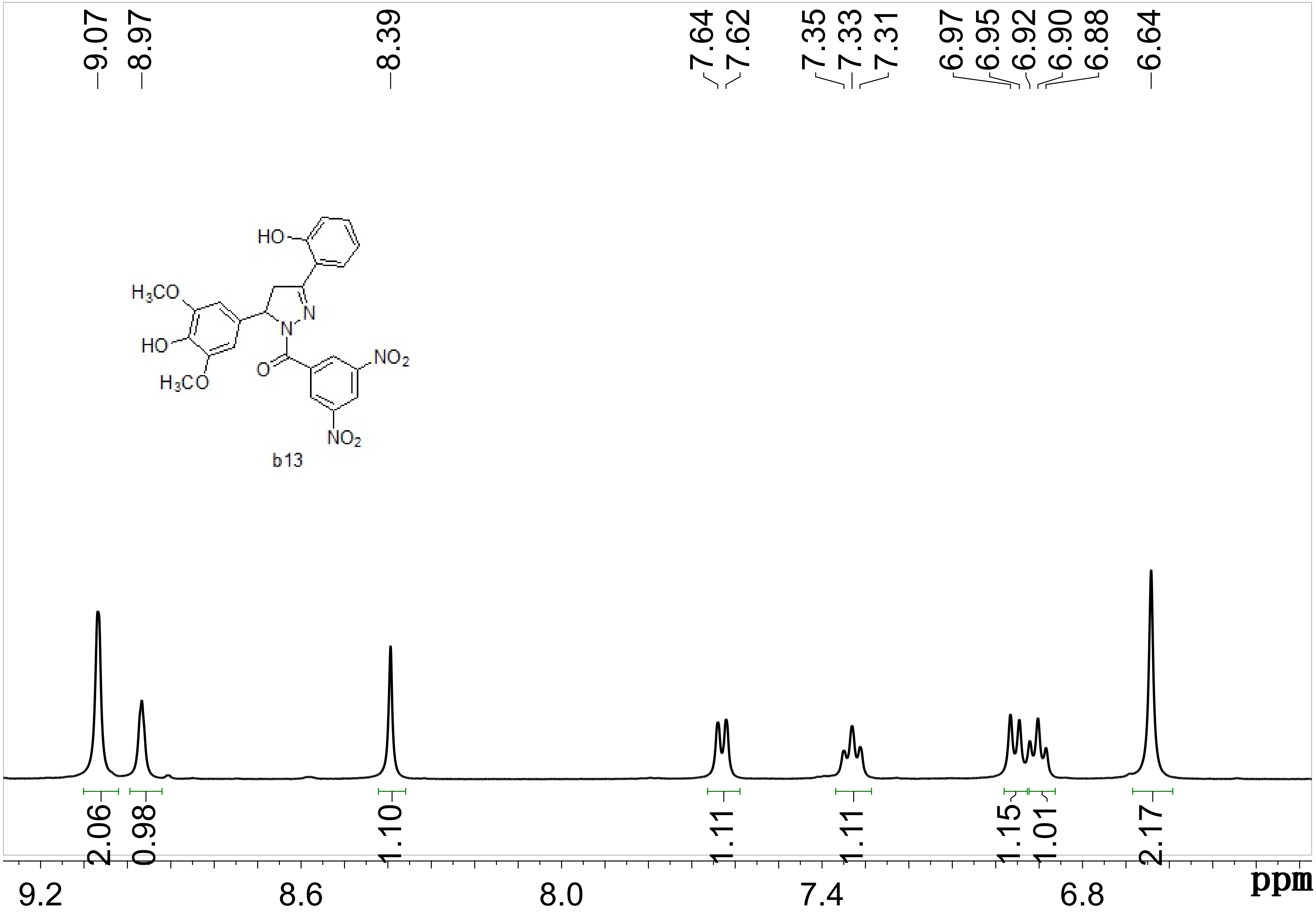
9.07
8.97
8.39
7.64
7.62
7.35
7.33
7.31
6.97
6.95
6.92
6.90
6.88
6.64
HO
H3CO
HO
H3CO
N
N
O
NO2
NO2
b13
2.06
0.98
1.10
1.11
1.11
1.15
1.01
2.17
9.2
8.6
8.0
7.4
6.8
ppm

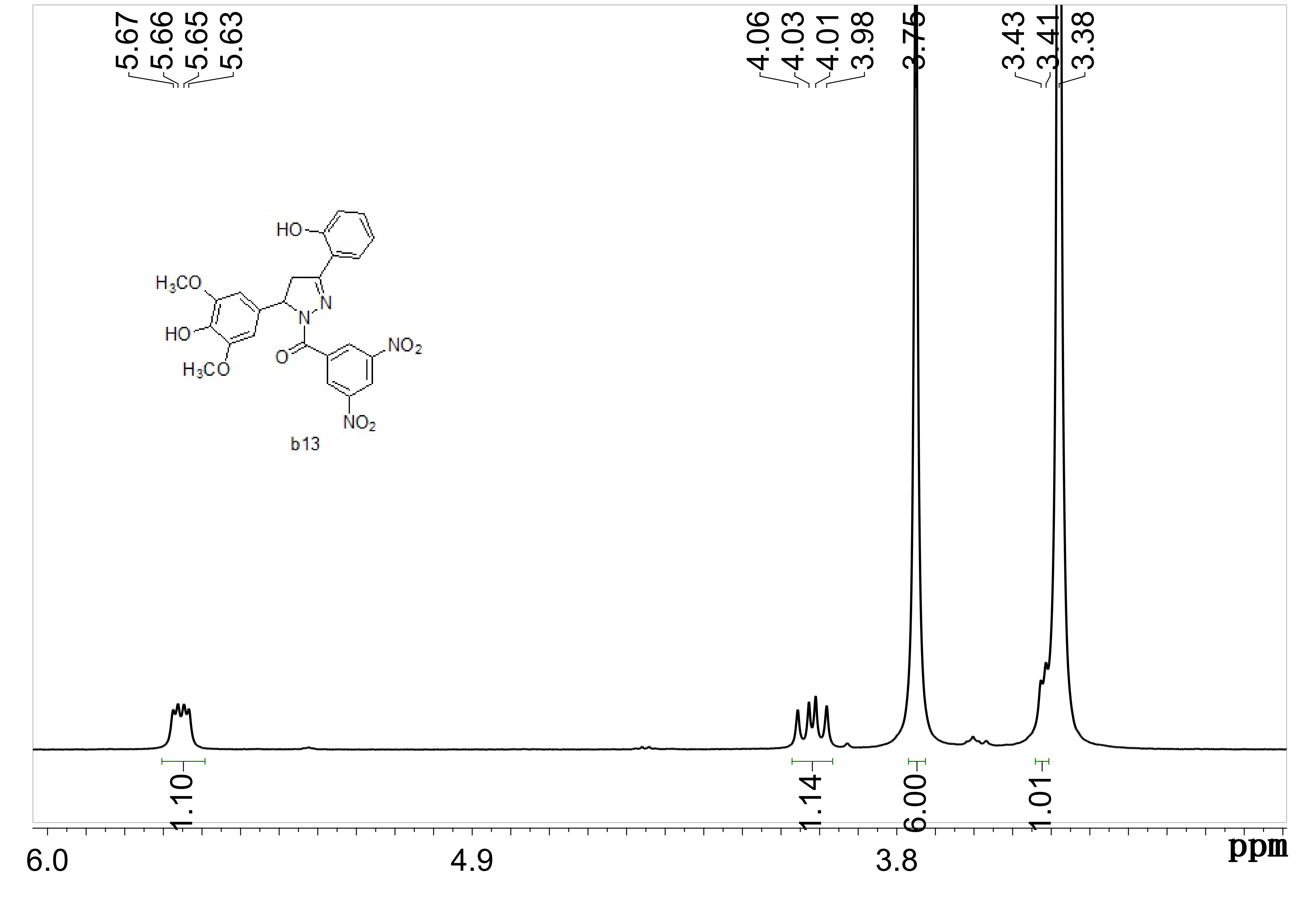

5.67
5.66
5.65
5.63
4.06
4.03
4.01
3.98
3.75
3.43
3.41
3.38
HO
H3CO
HO
H3CO
N
N
O
NO2
NO2
b13
1.10
1.14
6.00
1.01
6.0
4.9
3.8
ppm

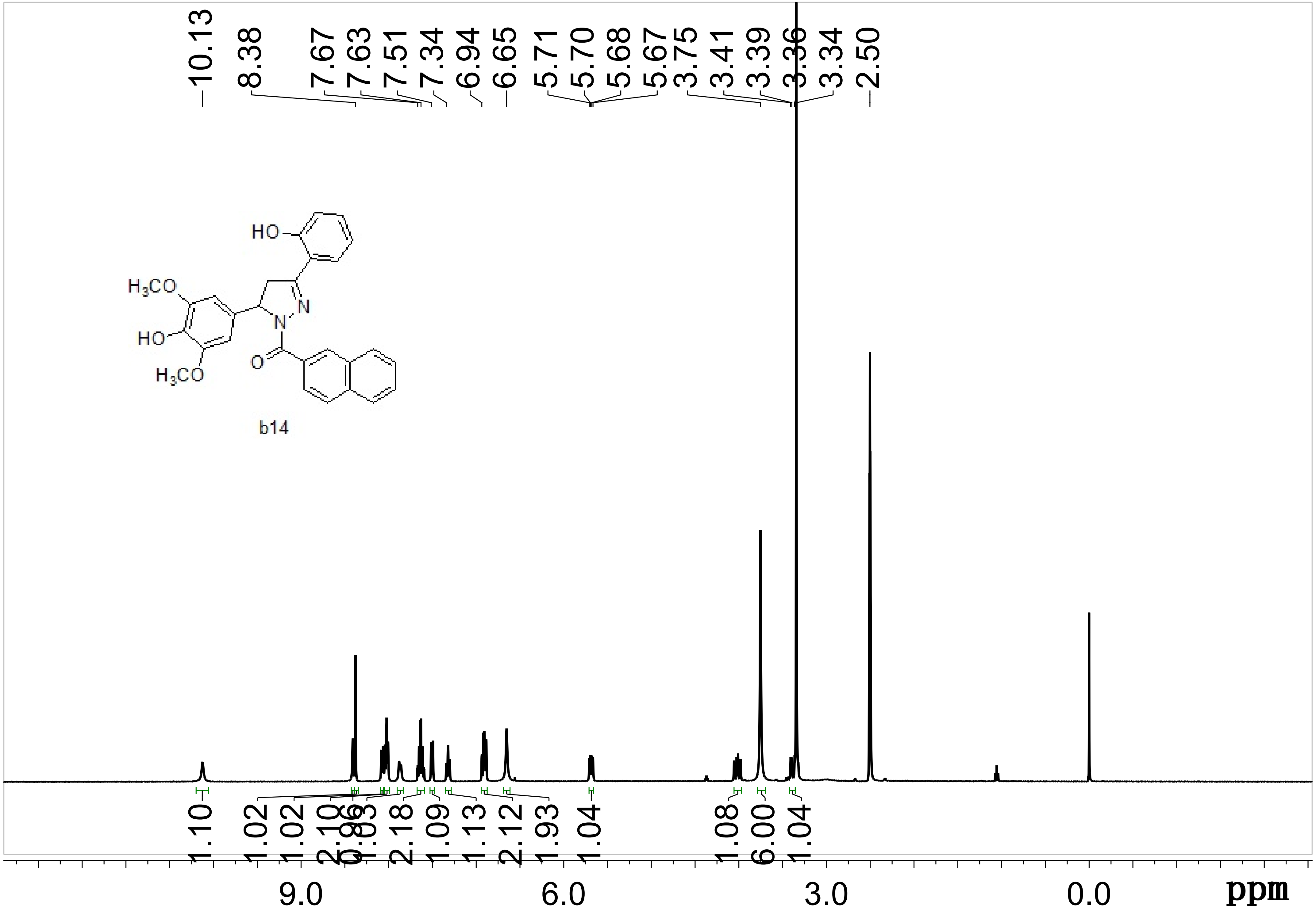
10.13
8.38
7.67
7.63
7.51
7.34
6.94
6.65
5.71
5.70
5.68
5.67
3.75
3.41
3.39
3.36
3.34
2.50
HO
H3CO
HO
H3CO
N
N
O
b14
1.10
1.02
1.02
2.10
1.93
2.18
1.09
1.13
2.12
1.93
1.04
1.08
6.00
1.04
9.0
6.0
3.0
0.0
ppm

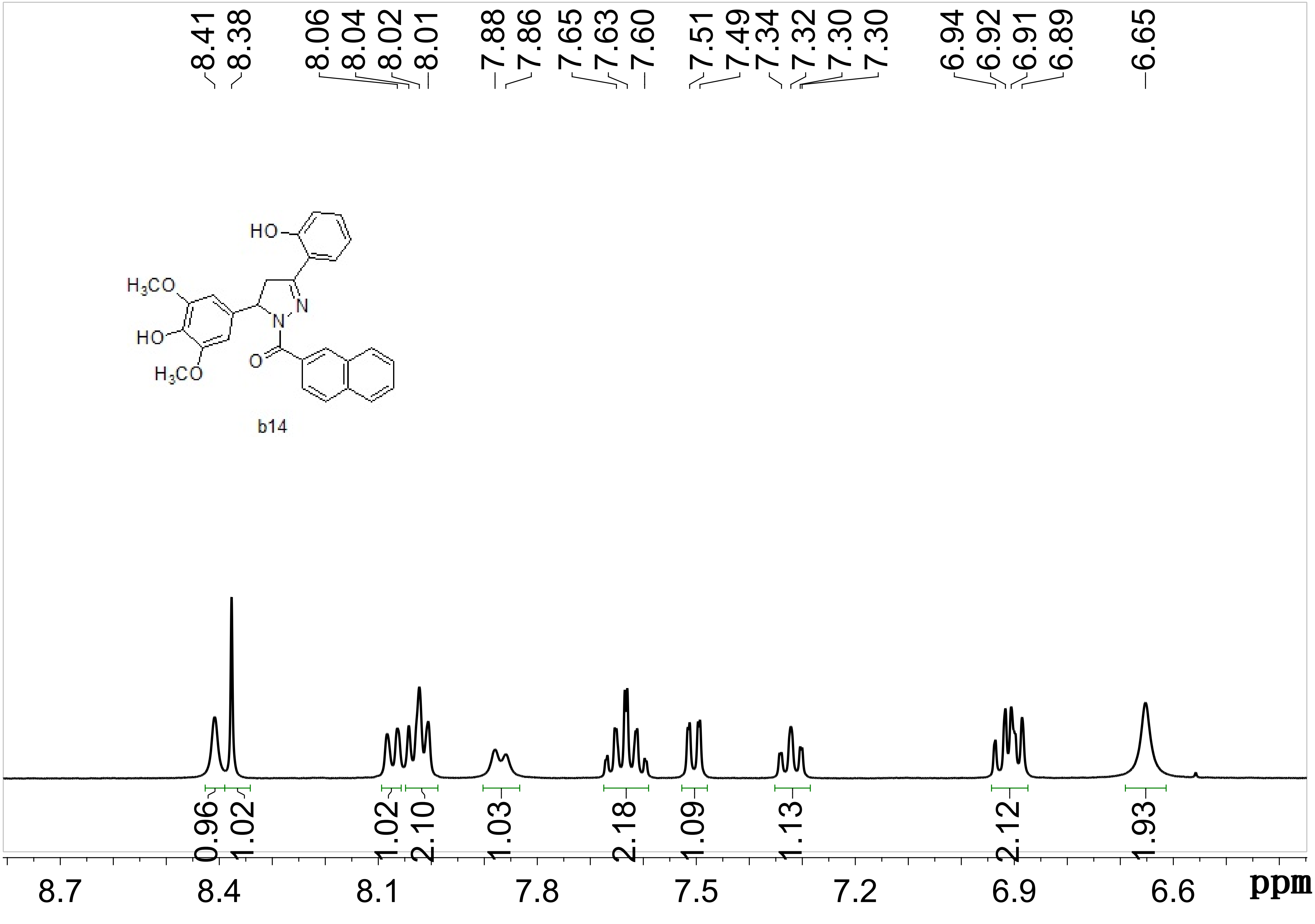
8.41 8.38 8.06 8.04 8.02 8.01 7.88 7.86 7.65 7.63 7.60 7.51 7.49 7.34 7.32 7.30 7.30 6.94 6.92 6.91 6.89 6.65
HO
H3CO
HO
H3CO
N
N
O
b14
0.96 1.02 1.02 2.10 1.03 2.18 1.09 1.13 2.12 1.93
8.7 8.4 8.1 7.8 7.5 7.2 6.9 6.6
ppm

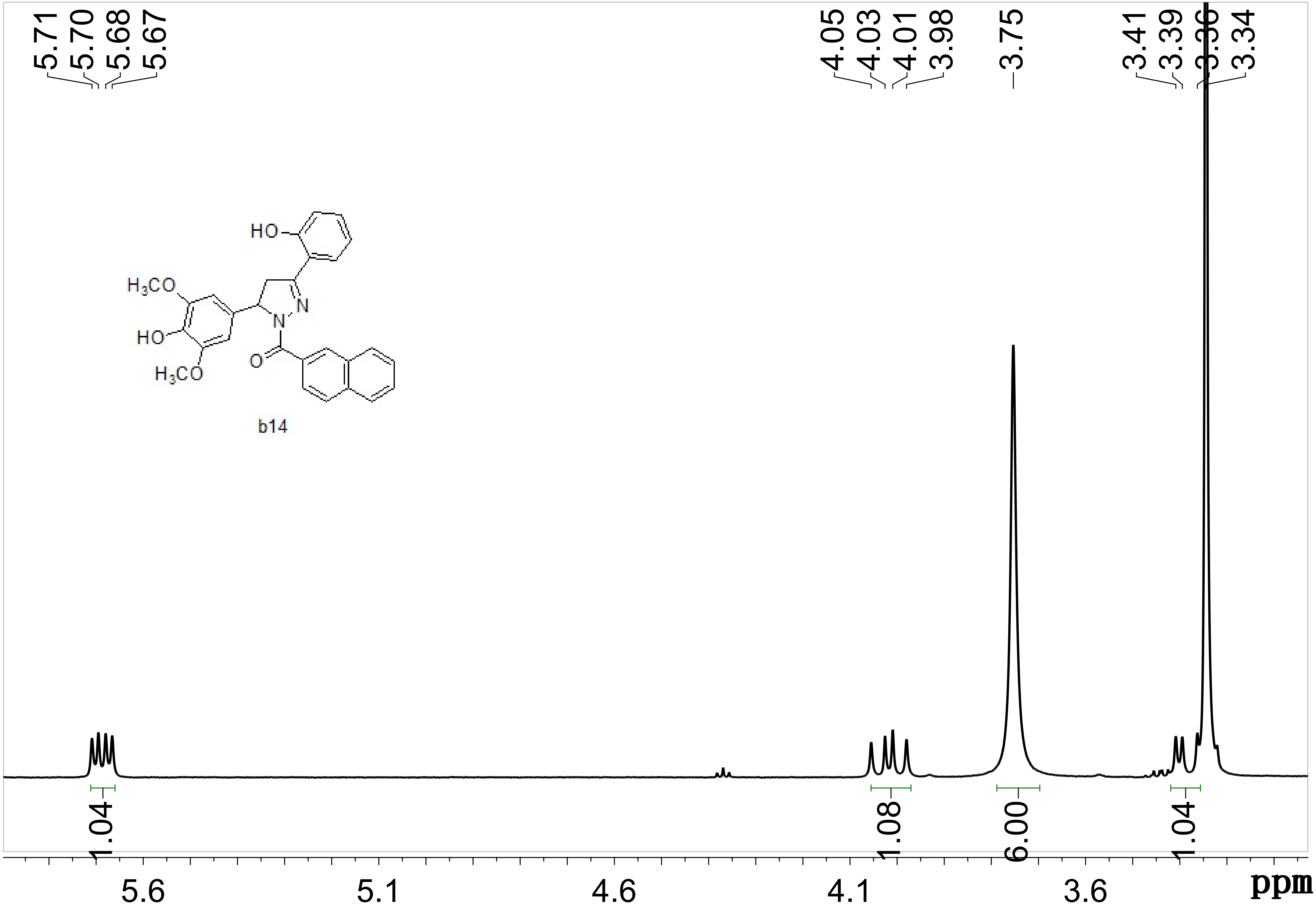
5.71
5.70
5.68
5.67
4.05
4.03
4.01
3.98
3.75
3.41
3.39
3.36
3.34
HO
H3CO
N
N
HO
O
H3CO
b14
1.04
1.08
6.00
1.04
5.6
5.1
4.6
4.1
3.6
ppm

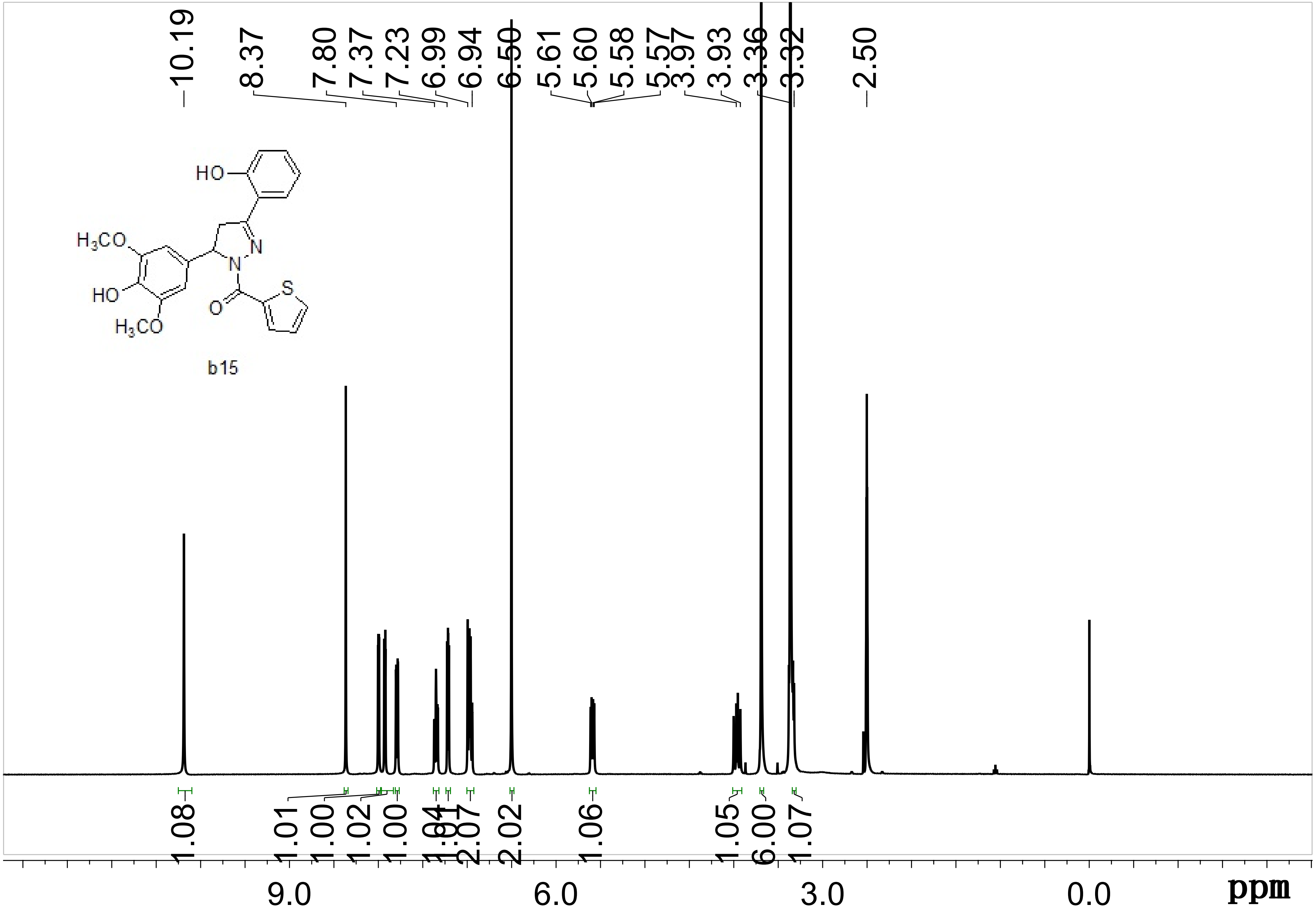

HO
H3CO
HO
H3CO
N
N
S
O
b15
10.19
8.37
7.80
7.37
7.23
6.99
6.94
6.50
5.61
5.60
5.58
5.57
3.97
3.93
3.36
3.32
2.50
1.08
1.01
1.00
1.02
1.00
1.04
1.01
2.07
2.02
1.06
1.05
6.00
1.07
9.0
6.0
3.0
0.0
ppm

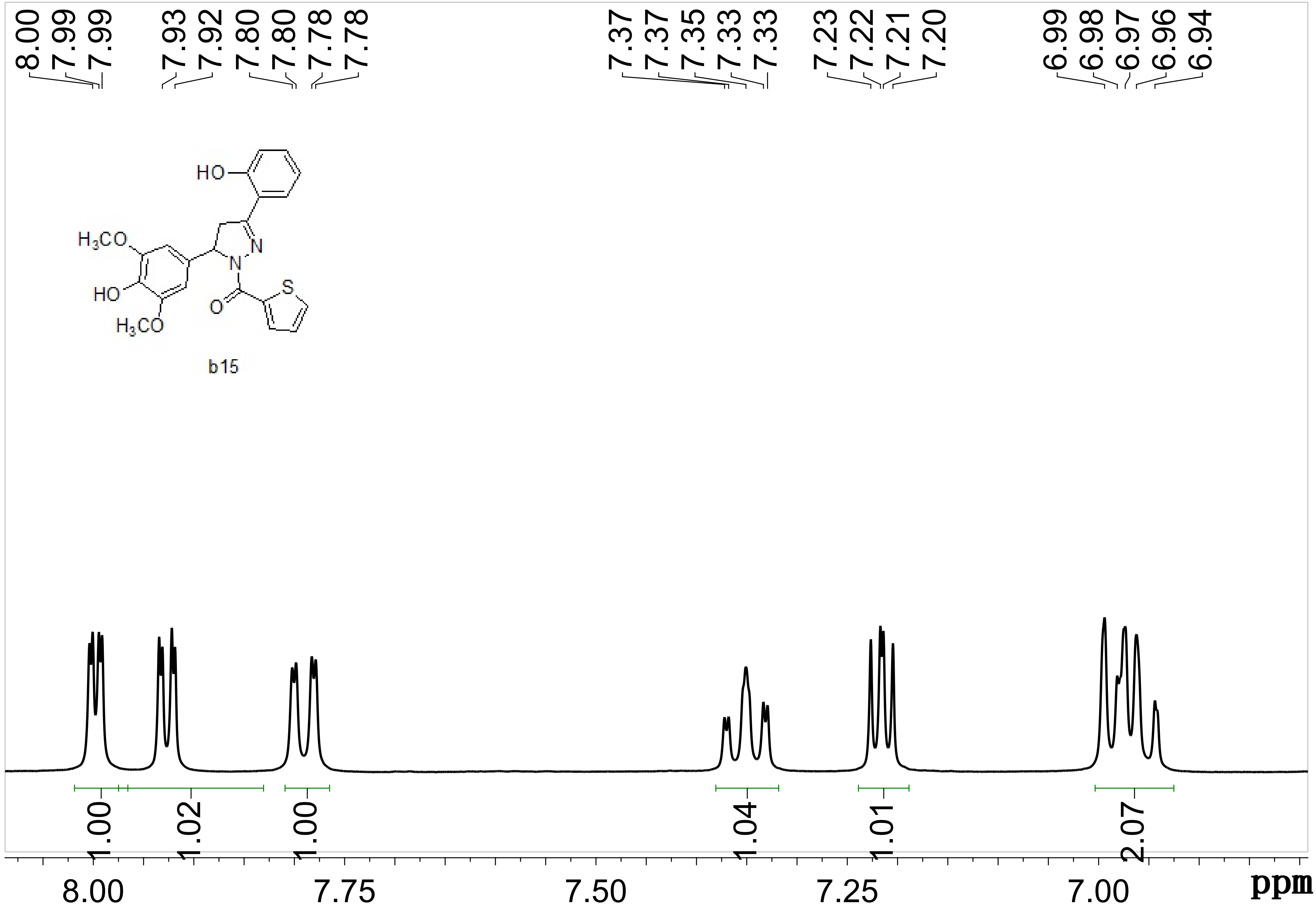
8.00 7.99 7.99 7.93 7.92 7.80 7.80 7.78 7.78
7.37 7.37 7.35 7.33 7.33 7.23 7.22 7.21 7.20
6.99 6.98 6.97 6.96 6.94
HO
H_3CO
HO
H_3CO
N
N
S
O
b15
1.00 1.02 1.00 1.04 1.01 2.07
8.00 7.75 7.50 7.25 7.00
ppm

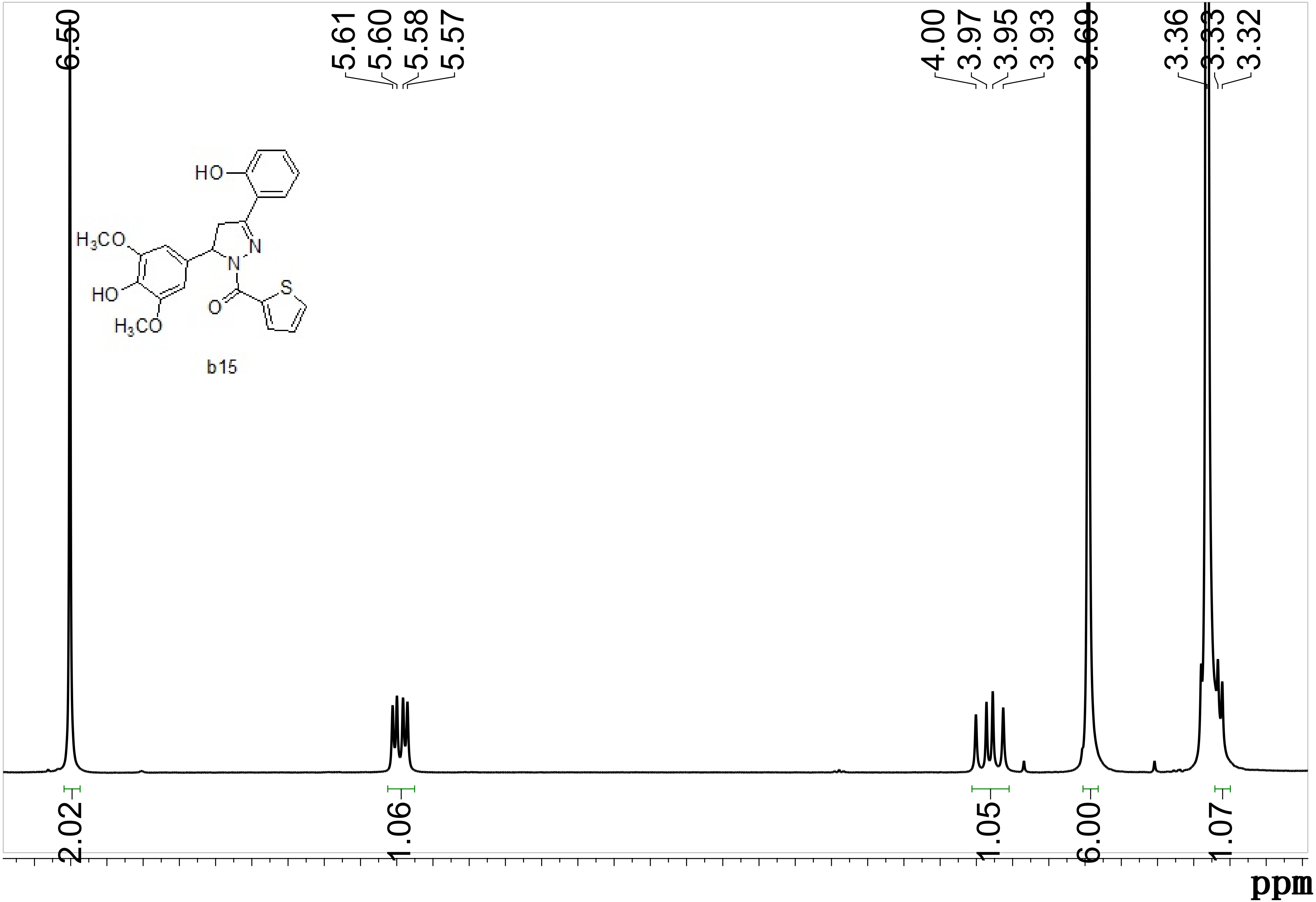
6.50
5.61
5.60
5.58
5.57
4.00
3.97
3.95
3.93
3.69
3.36
3.33
3.32
HO
H_3CO
HO
H_3CO
N
N
O
S
b15
2.02
1.06
1.05
6.00
1.07
ppm

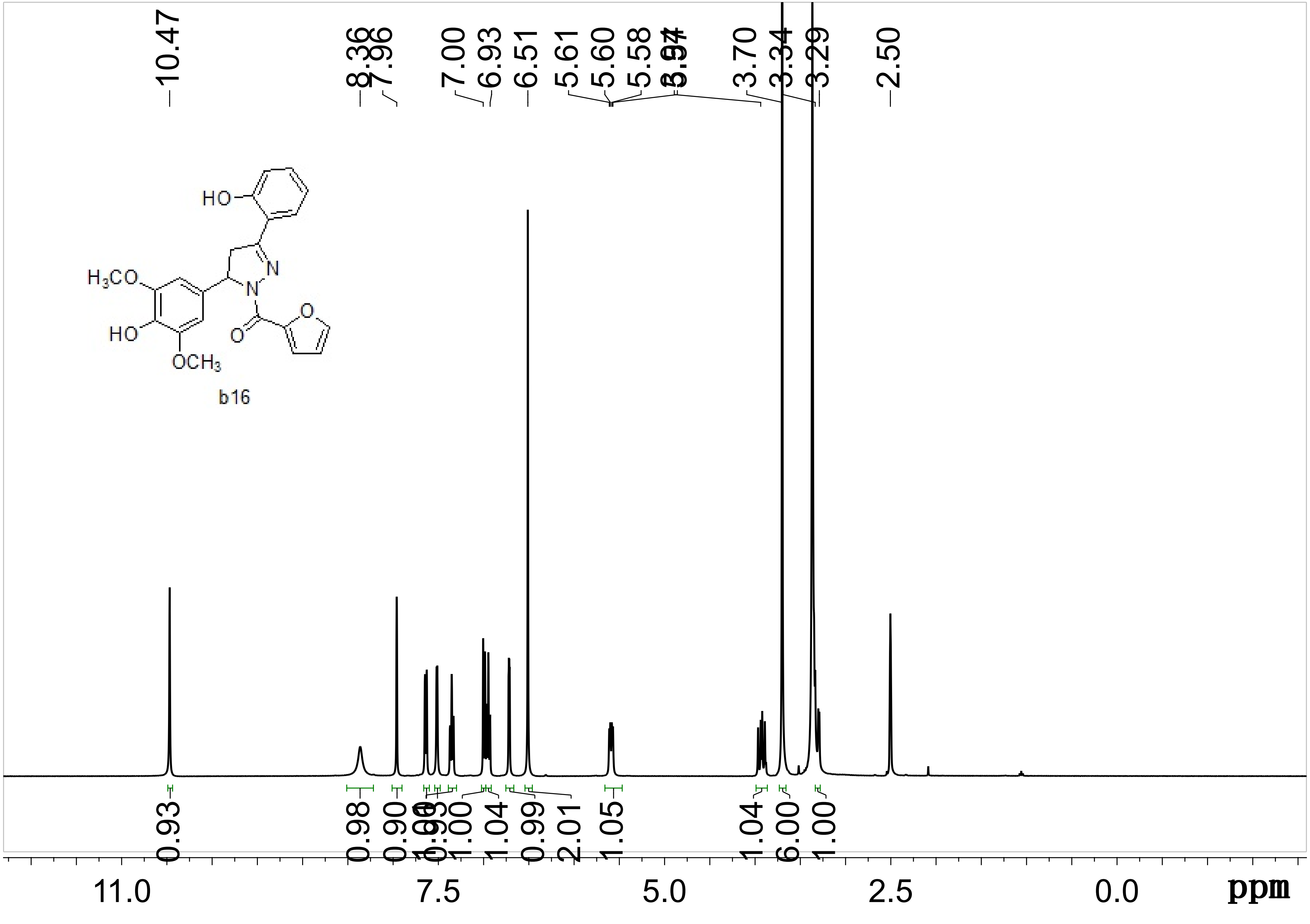
10.47
8.36
7.96
7.00
6.93
6.51
5.61
5.60
5.58
3.70
3.34
3.29
2.50
HO
H_3CO
HO
OCH_3
N
N
O
O
b16
0.93
0.98
0.90
1.00
1.04
0.99
2.01
1.05
1.04
6.00
1.00
11.0
7.5
5.0
2.5
0.0
ppm

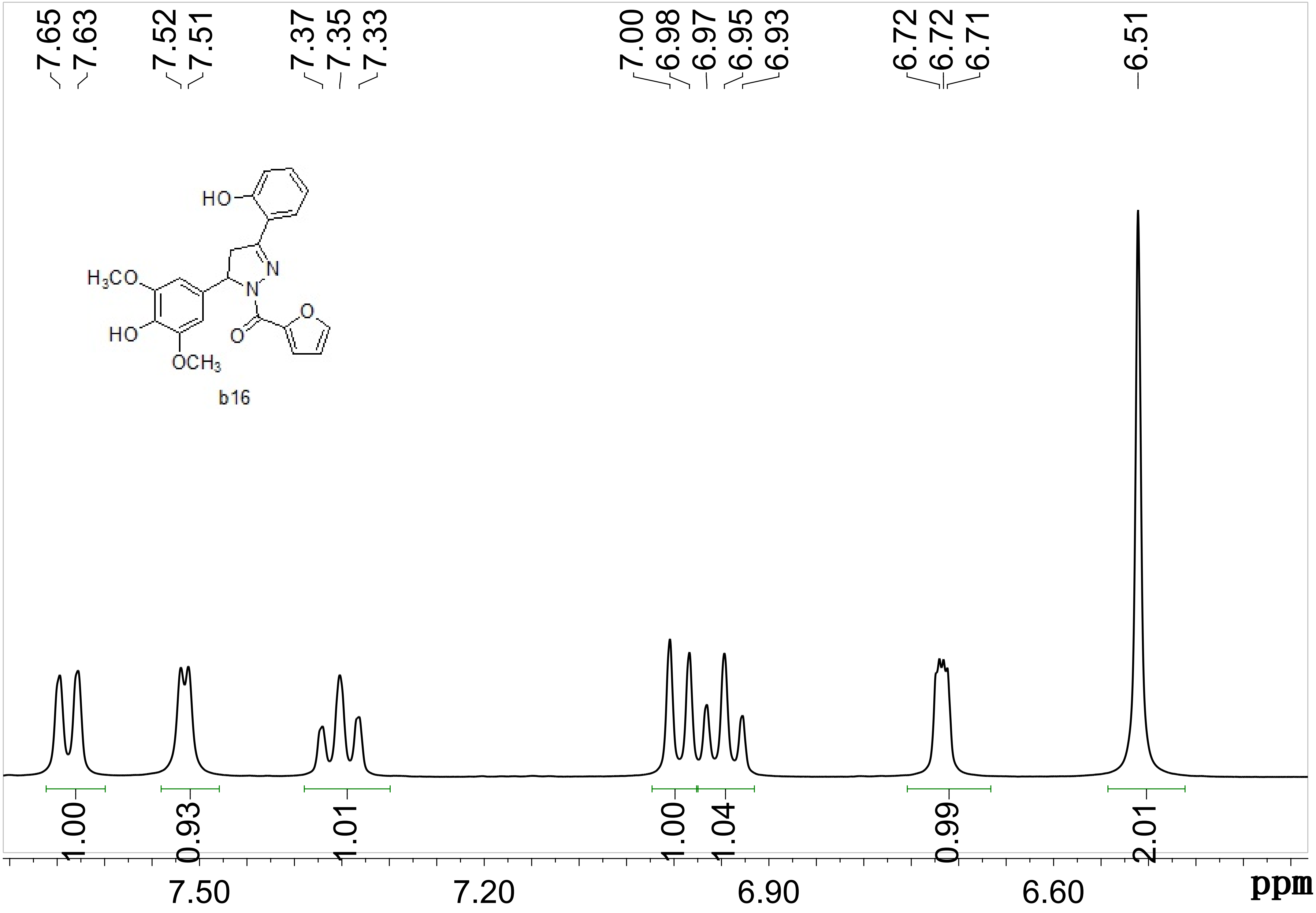

7.65
7.63
7.52
7.51
7.37
7.35
7.33
7.00
6.98
6.97
6.95
6.93
6.72
6.72
6.71
6.51
HO
H3CO
HO
OCH3
N
N
O
O
b16
1.00
0.93
1.01
1.00
1.04
0.99
2.01
7.50
7.20
6.90
6.60
ppm

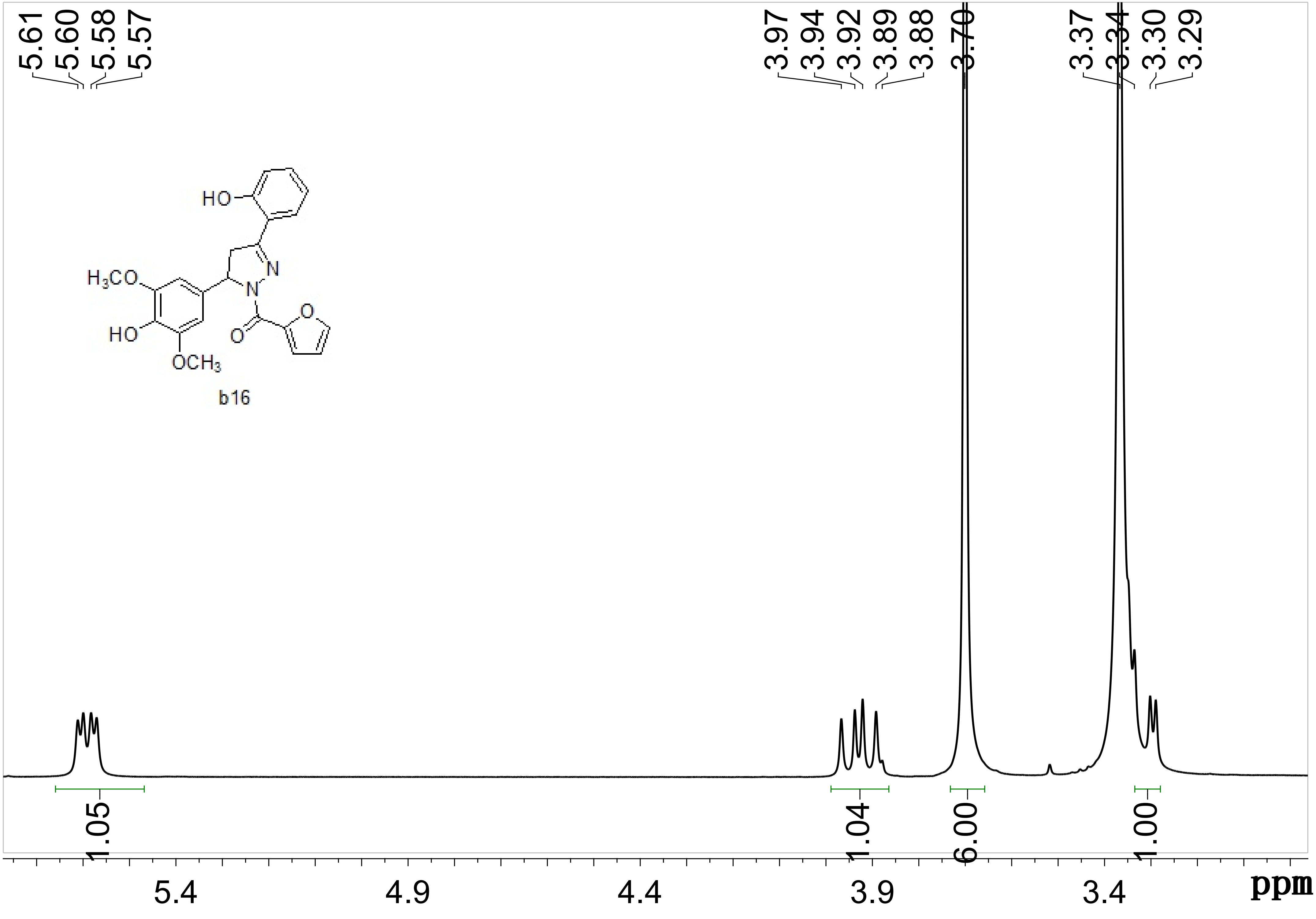
5.61
5.60
5.58
5.57
3.97
3.94
3.92
3.89
3.88
3.70
3.37
3.34
3.30
3.29
HO
H3CO
HO
OCH3
N
N
O
O
b16
1.05
1.04
6.00
1.00
5.4
4.9
4.4
3.9
3.4
ppm

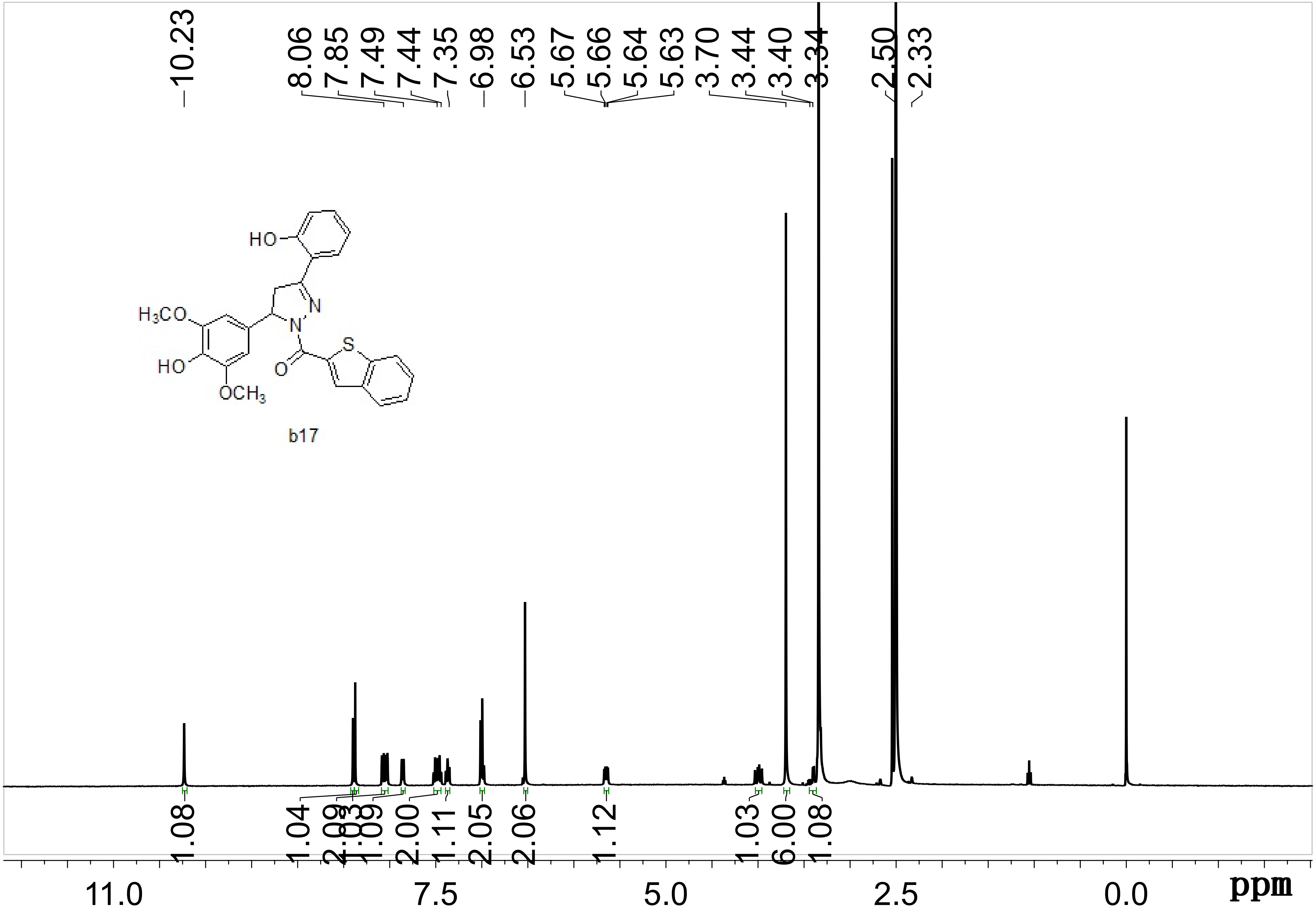
10.23
8.06
7.85
7.49
7.44
7.35
6.98
6.53
5.67
5.66
5.64
5.63
3.70
3.44
3.40
3.34
2.50
2.33
HO
H_3CO
HO
OCH_3
N
N
S
O
b17
1.08
1.04
2.09
1.03
1.09
2.00
1.11
2.05
2.06
1.12
1.03
6.00
1.08
11.0
7.5
5.0
2.5
0.0
ppm

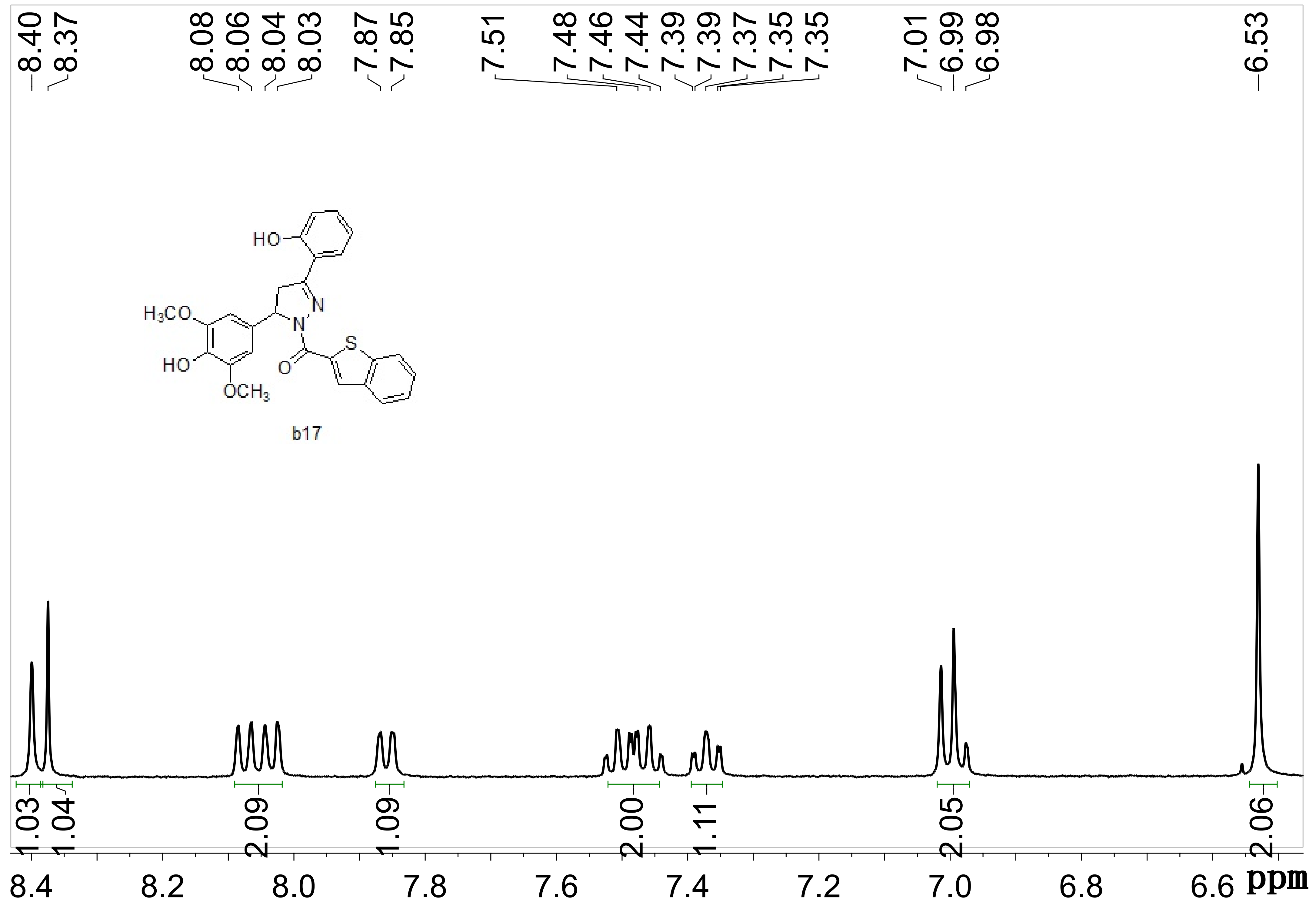

8.40
8.37
8.08
8.06
8.04
8.03
7.87
7.85
7.51
7.48
7.46
7.44
7.39
7.39
7.37
7.35
7.35
7.01
6.99
6.98
6.53
HO
H3CO
N
N
S
HO
O
OCH3
b17
1.03
1.04
2.09
1.09
2.00
1.11
2.05
2.06
8.4
8.2
8.0
7.8
7.6
7.4
7.2
7.0
6.8
6.6
ppm

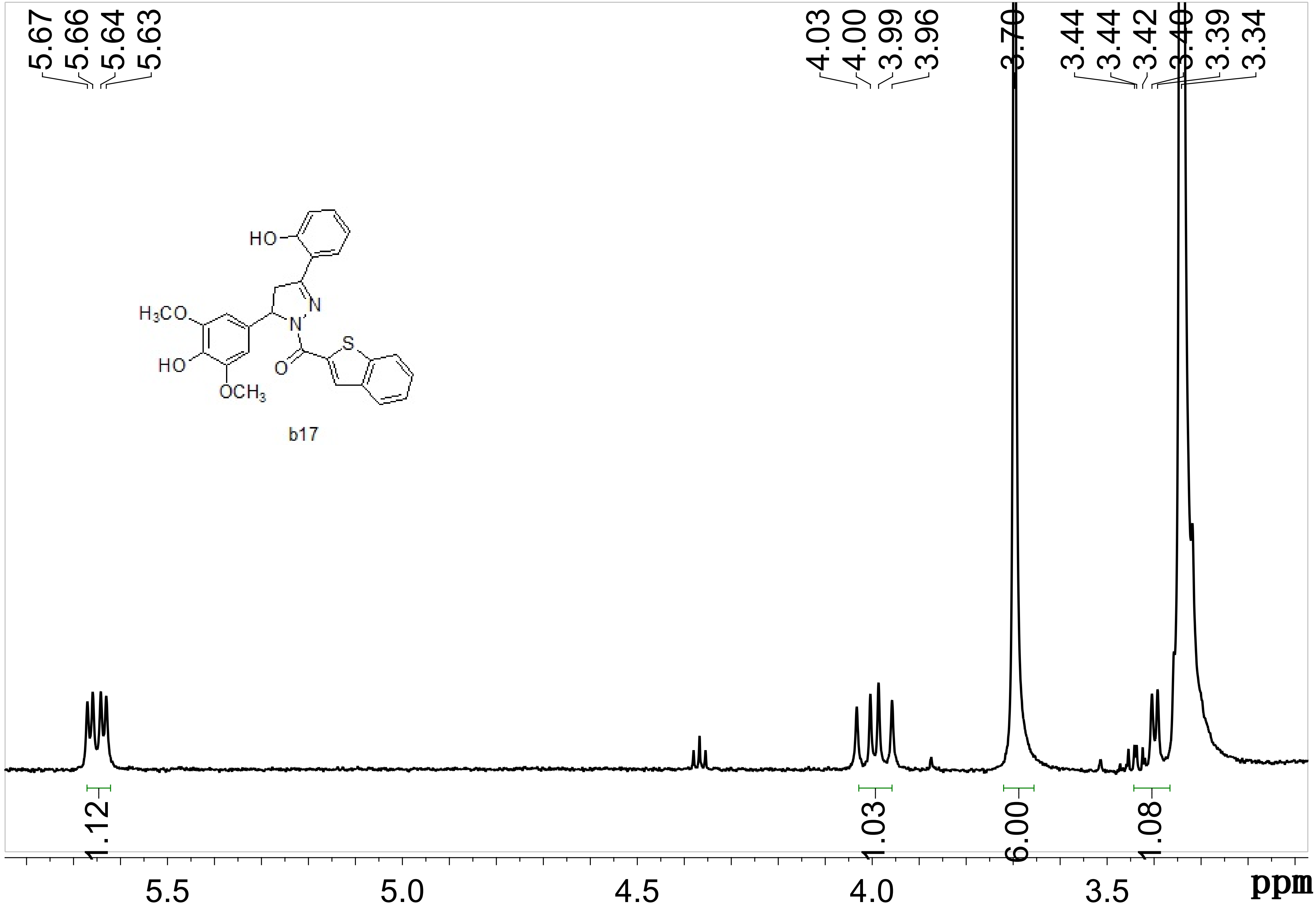
5.67 5.66 5.64 5.63
4.03 4.00 3.99 3.96
3.70
3.44 3.44 3.42 3.40 3.39 3.34
HO
H_3CO
HO
OCH_3
N
N
S
O
b17
1.12
1.03
6.00
1.08
5.5
5.0
4.5
4.0
3.5
ppm

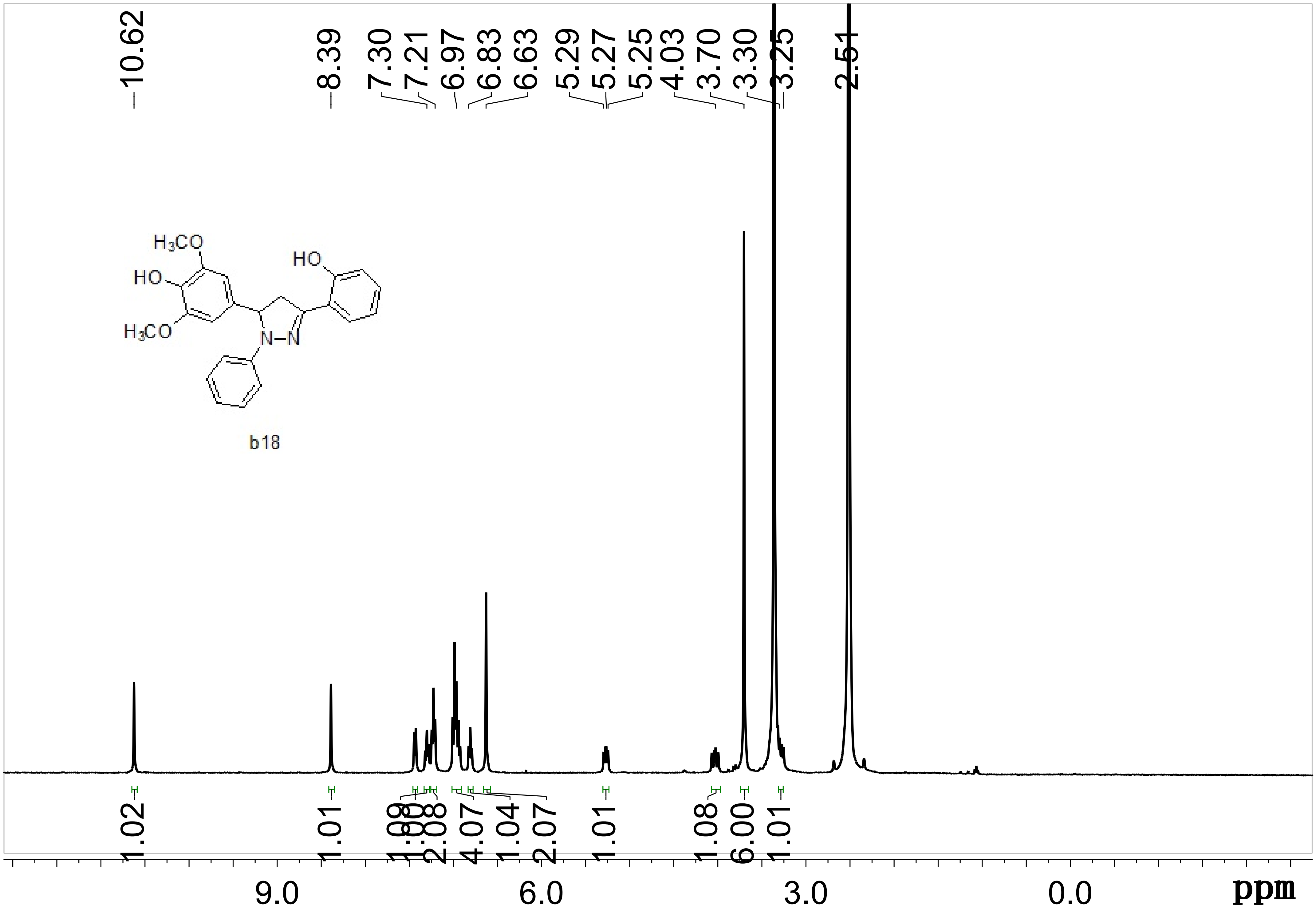

10.62
8.39
7.30
7.21
6.97
6.83
6.63
5.29
5.27
5.25
4.03
3.70
3.30
3.25
2.51
H3CO
HO
HO
H3CO
N–N
b18
1.02
1.01
2.08
4.07
1.04
2.07
1.01
1.08
6.00
1.01
9.0
6.0
3.0
0.0
ppm

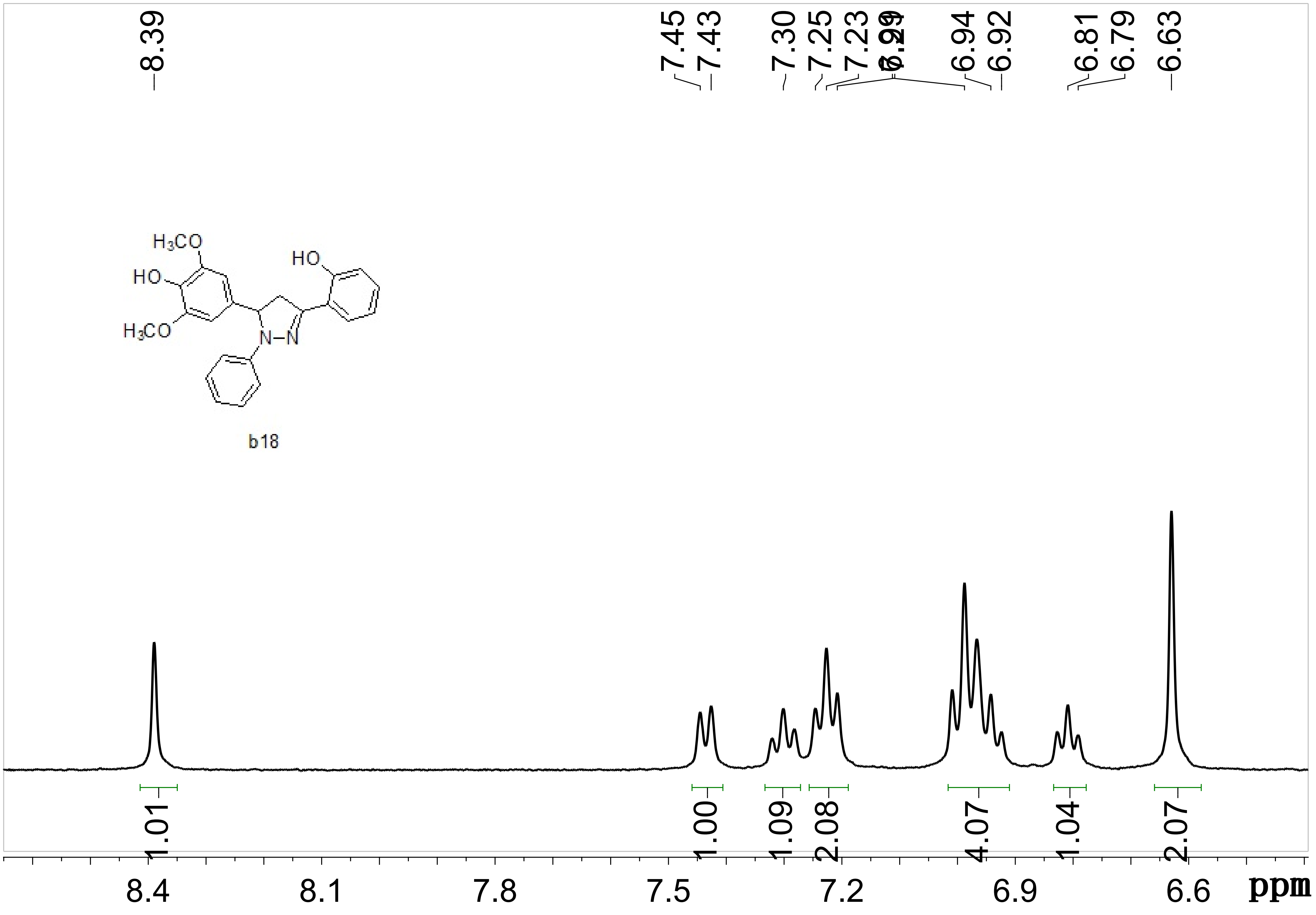

8.39
7.45
7.43
7.30
7.25
7.23
6.94
6.92
6.81
6.79
6.63
H3CO
HO
H3CO
HO
N–N
b18
1.01
1.00
1.09
2.08
4.07
1.04
2.07
8.4
8.1
7.8
7.5
7.2
6.9
6.6
ppm

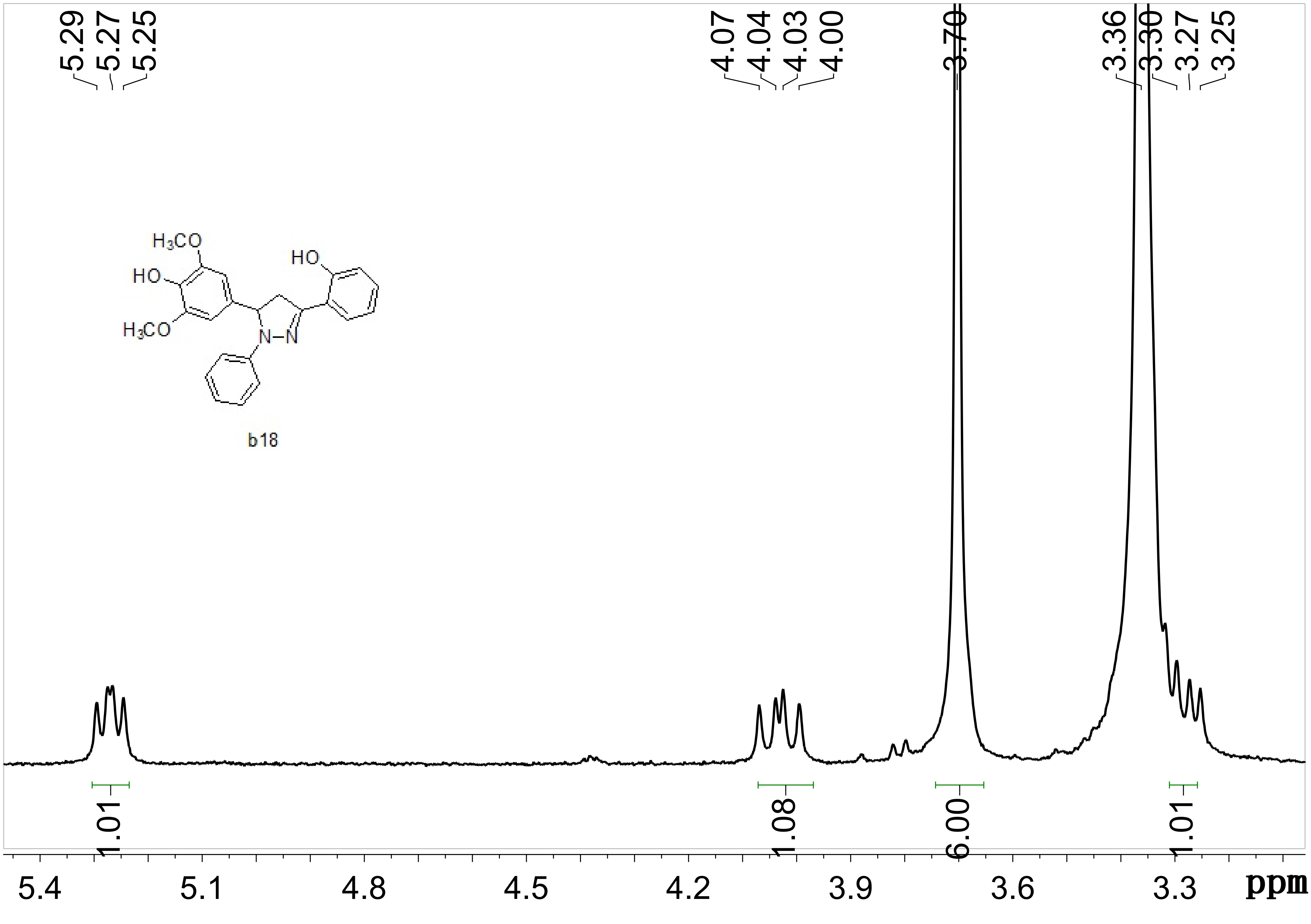
5.29
5.27
5.25
4.07
4.04
4.03
4.00
3.70
3.36
3.30
3.27
3.25
H3CO
HO
H3CO
HO
N–N
b18
1.01
1.08
6.00
1.01
5.4
5.1
4.8
4.5
4.2
3.9
3.6
3.3
ppm

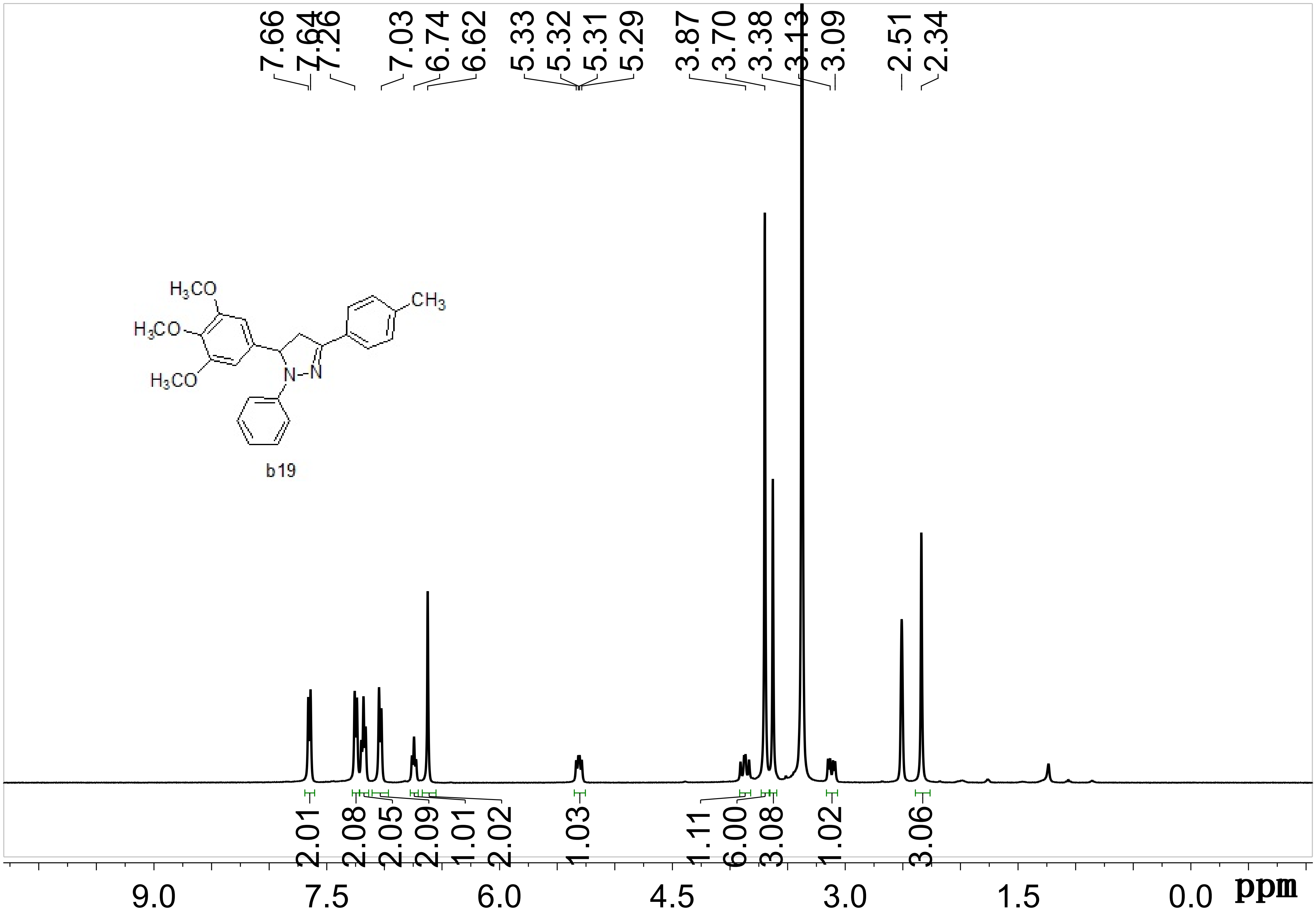
7.66
7.64
7.26
7.03
6.74
6.62
5.33
5.32
5.31
5.29
3.87
3.70
3.38
3.13
3.09
2.51
2.34
H3CO
H3CO
H3CO
CH3
N-N
b19
2.01
2.08
2.05
2.09
1.01
2.02
1.03
1.11
6.00
3.08
1.02
3.06
9.0
7.5
6.0
4.5
3.0
1.5
0.0
ppm

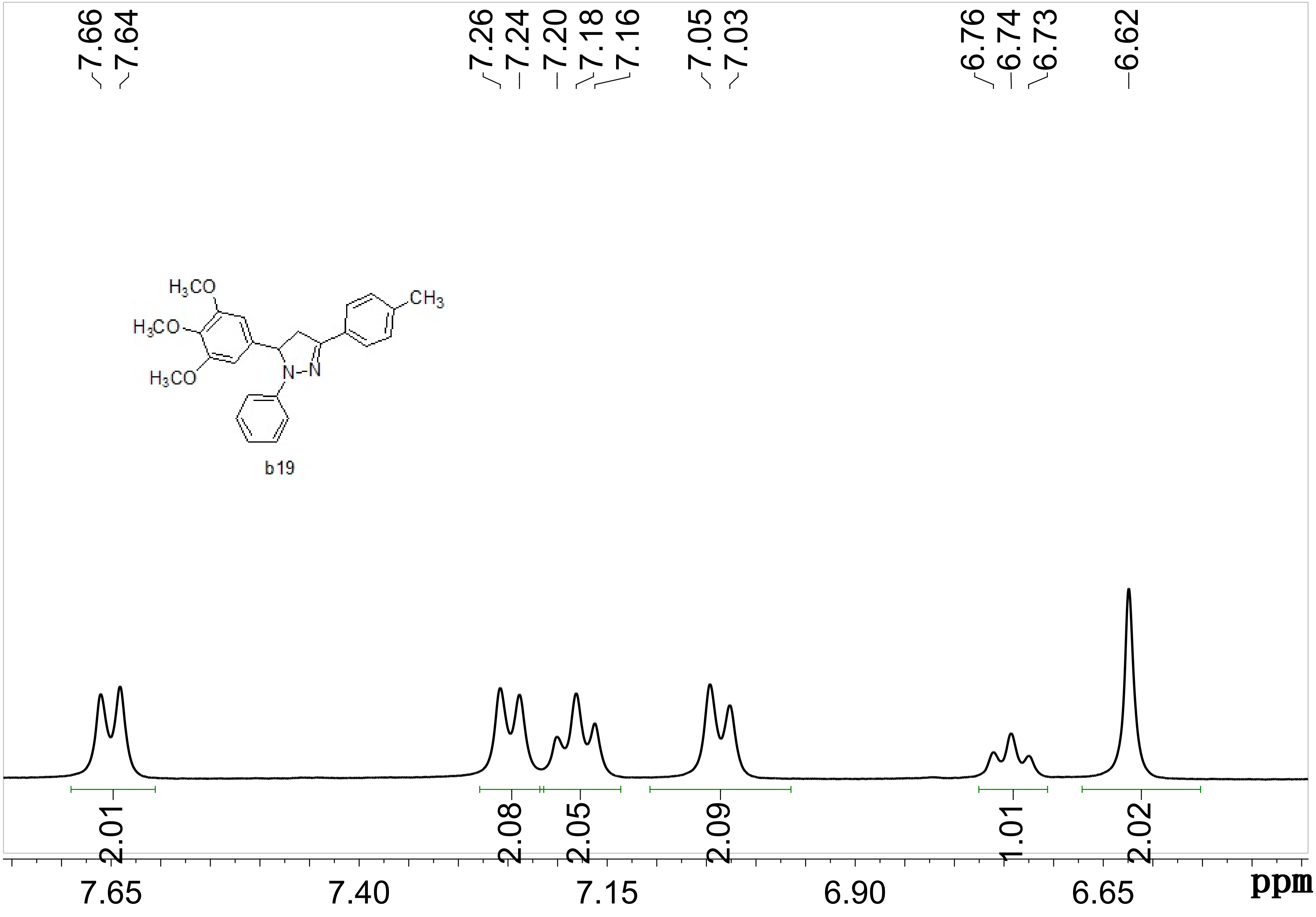
7.66
7.64
7.26
7.24
7.20
7.18
7.16
7.05
7.03
6.76
6.74
6.73
6.62
H3CO
H3CO
H3CO
CH3
N
N
b19
2.01
2.08
2.05
2.09
1.01
2.02
7.65
7.40
7.15
6.90
6.65
ppm

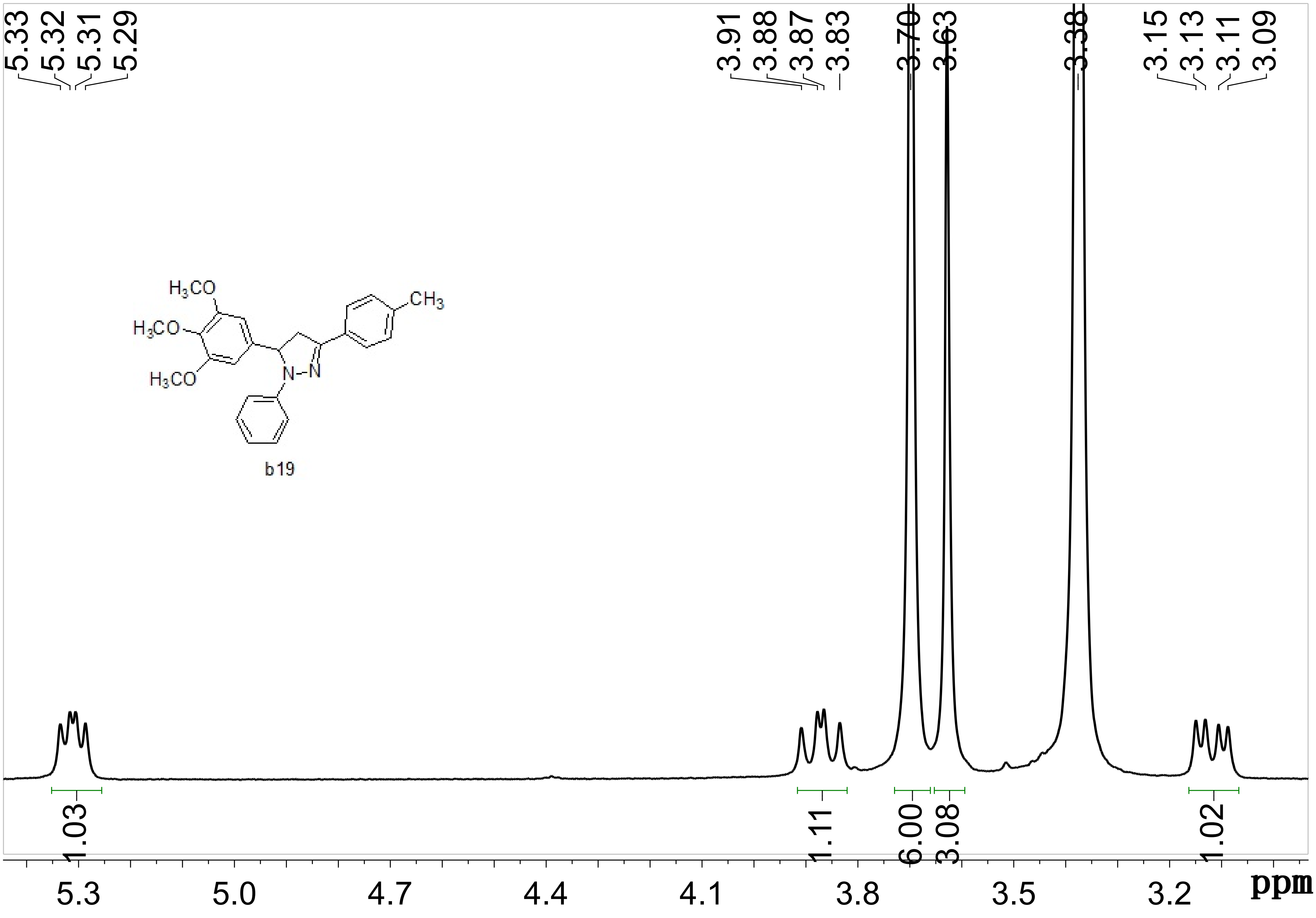
5.33
5.32
5.31
5.29
3.91
3.88
3.87
3.83
3.70
3.63
3.38
3.15
3.13
3.11
3.09
H3CO
H3CO
H3CO
CH3
N
N
b19
1.03
1.11
6.00
3.08
1.02
5.3
5.0
4.7
4.4
4.1
3.8
3.5
3.2
ppm
