# Supplementary material for: Synthesis and Evaluation of New Pyrazoline Derivatives as Potential Anticancer Agents in HepG-2 Cell Line
Source: Molecules. 2017 Mar 16;22(3):467. doi: 10.3390/molecules22030467 (PMC6155299; doi:10.3390/molecules22030467)
Supplement: Supplementary file 1 [file molecules-22-00467-s001.zip › Supplementary Materials/b17(13C-NMR).pdf]

Synthesis and Evaluation of New Pyrazoline Derivatives as Potential Anticancer Agents in HepG-2 Cell Line

Weijie Xu [1,†], Ying Pan [1,†], Hong Wang [1,†], Haiyan Li [2], Qing Peng [3], Duncan Wei [1], Cheng Chen [1] and Jinhong Zheng [1,*]

1 Department of Chemistry, Shantou University Medical College, Shantou 515041, Guangdong, China; 13536920534@163.com (W.X.); ypan@stu.edu.cn (Y.P.); 13592851552@163.com (H.W.); weiduncan2012@163.com (D.W.); 13411963192@163.com (C.C.); jhzheng@stu.edu.cn (J.Z)

2 Department of Pharmacology, Shantou University Medical College, Shantou 515041, Guangdong, China; 13066360950@163.com (H.L.)

3 Department of Hepatobiliary Surgery II, Zhujiang Hospital of Southern Medical University, Guangzhou 510280, Guangdong, China; zhujiangzhuanhua@163.com (Q.P.)

* Correspondence: jhzheng@stu.edu.cn; Tel.: +86-754-8890-0499; Fax: +86-754-8855-7562

† These authors contributed equally to this work.

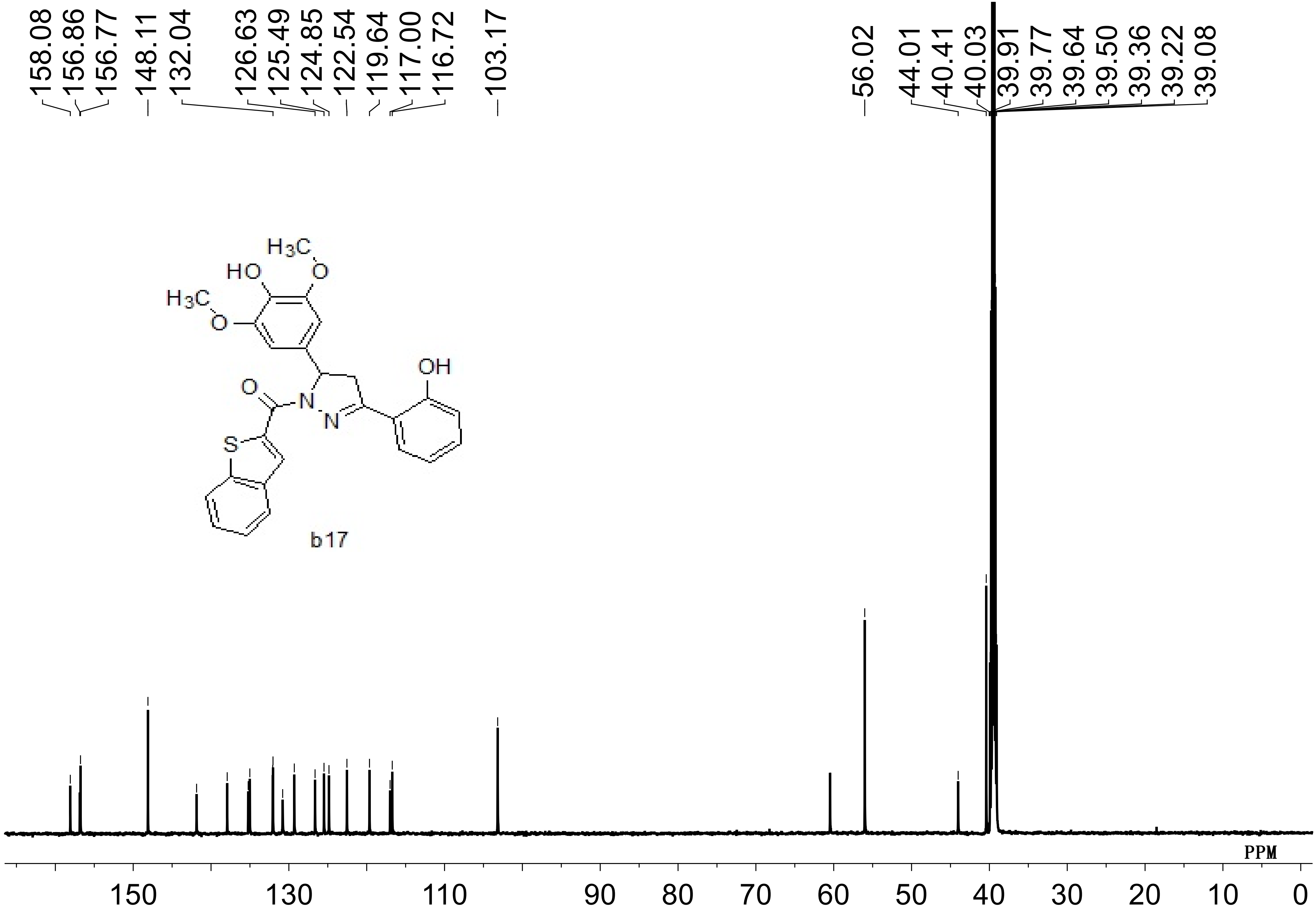
158.08
156.86
156.77
148.11
132.04
126.63
125.49
124.85
122.54
119.64
117.00
116.72
103.17
56.02
44.01
40.41
40.03
39.91
39.77
39.64
39.50
39.36
39.22
39.08
H3C
HO
O
H3C
O
OH
O
N
N
S
b17
PPM
150
130
110
90
80
70
60
50
40
30
20
10
0

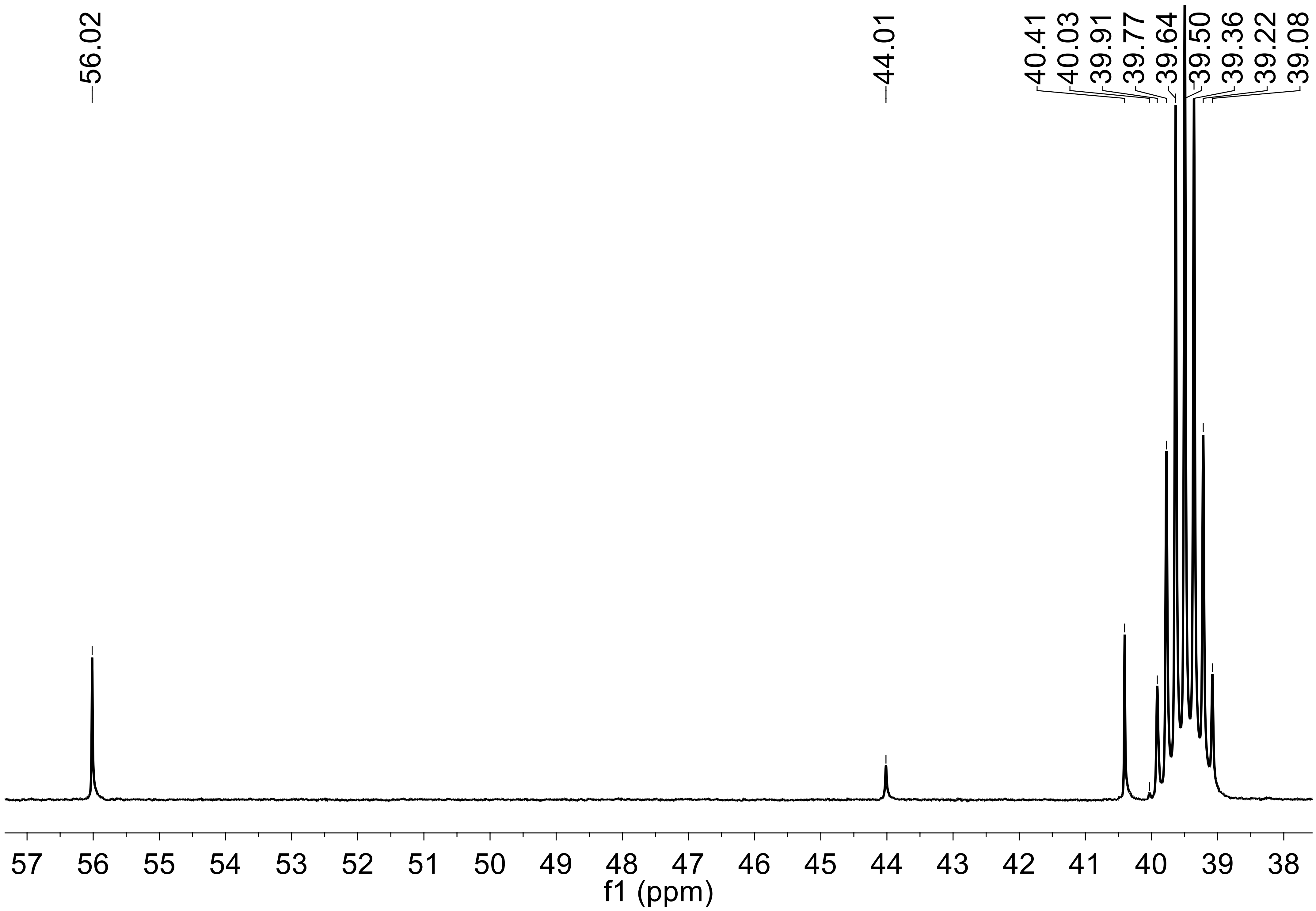
56.02
44.01
40.41
40.03
39.91
39.77
39.64
39.50
39.36
39.22
39.08
57 56 55 54 53 52 51 50 49 48 47 46 45 44 43 42 41 40 39 38
f1 (ppm)

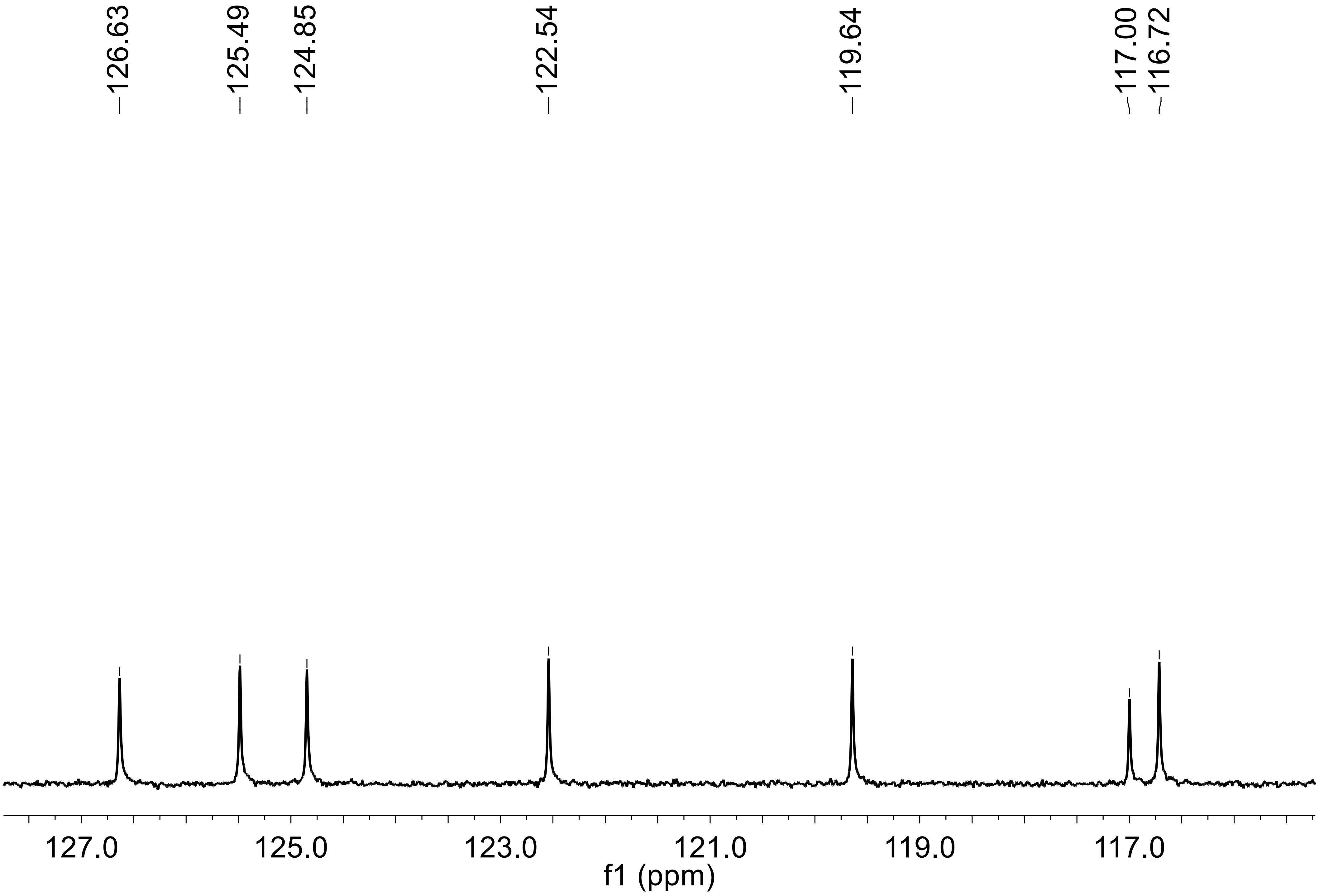
126.63
125.49
124.85
122.54
119.64
117.00
116.72
127.0
125.0
123.0
121.0
119.0
117.0
f1 (ppm)

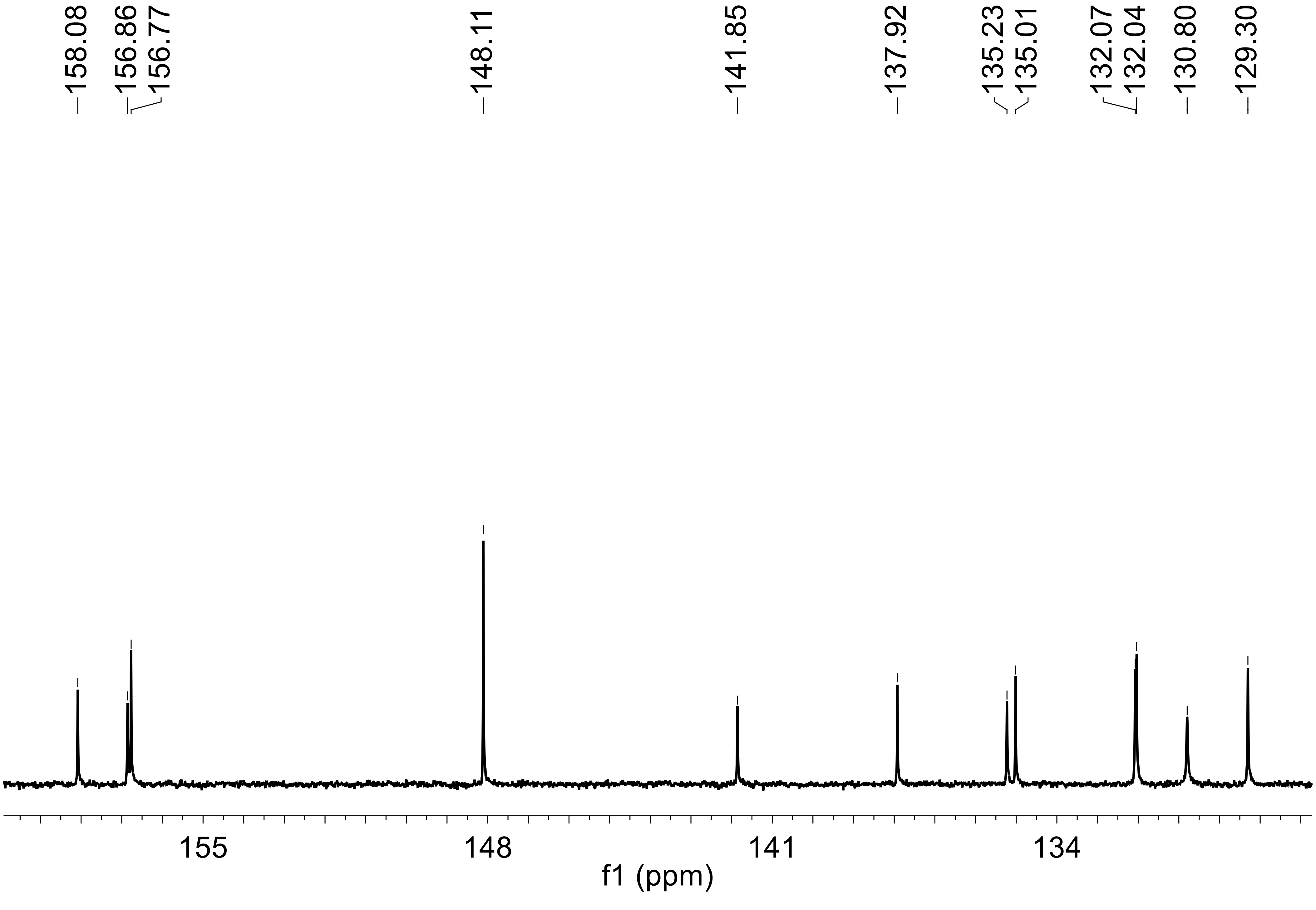

158.08
156.86
156.77
148.11
141.85
137.92
135.23
135.01
132.07
132.04
130.80
129.30
155
148
141
134
f1 (ppm)
