# Supplementary material for: Synthesis and Evaluation of New Pyrazoline Derivatives as Potential Anticancer Agents in HepG-2 Cell Line
Source: Molecules. 2017 Mar 16;22(3):467. doi: 10.3390/molecules22030467 (PMC6155299; doi:10.3390/molecules22030467)

Synthesis and Evaluation of New Pyrazoline Derivatives as Potential Anticancer Agents in HepG-2 Cell Line

Weijie Xu [1,†], Ying Pan [1,†], Hong Wang [1,†], Haiyan Li [2], Qing Peng [3], Duncan Wei [1], Cheng Chen [1] and Jinhong Zheng [1,*]

[1] Department of Chemistry, Shantou University Medical College, Shantou 515041, Guangdong, China; 13536920534@163.com (W.X.); ypan@stu.edu.cn (Y.P.); 13592851552@163.com (H.W.); weiduncan2012@163.com (D.W.); 13411963192@163.com (C.C.); jhzheng@stu.edu.cn (J.Z)

[2] Department of Pharmacology, Shantou University Medical College, Shantou 515041, Guangdong, China; 13066360950@163.com (H.L.)

[3] Department of Hepatobiliary Surgery II, Zhujiang Hospital of Southern Medical University, Guangzhou 510280, Guangdong, China; zhujiangzhuanhua@163.com (Q.P.)

* Correspondence: jhzheng@stu.edu.cn; Tel.: +86-754-8890-0499; Fax: +86-754-8855-7562

† These authors contributed equally to this work.

Structure of compound **b17**:

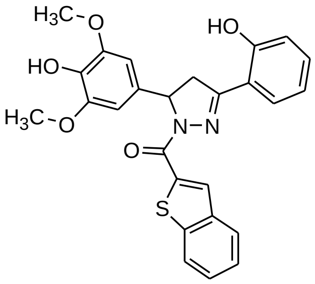

Analysis:	b17	Acquisition Date:	2/28/2017
Name: b17			
Comment:	ESI Negative	Operator:	TIPC

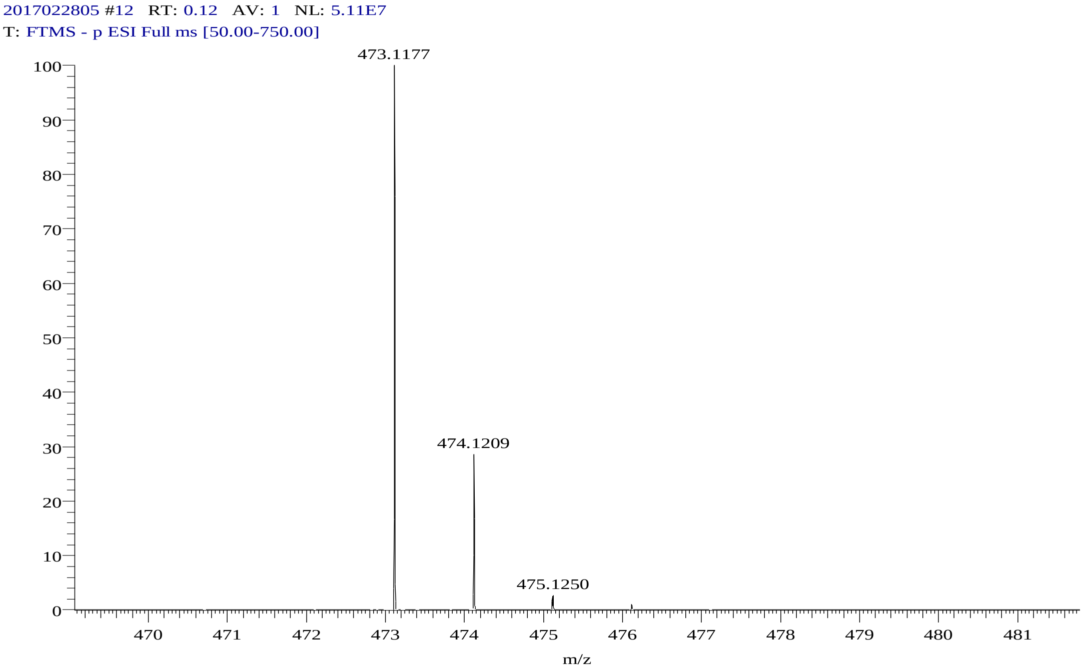

Supplement: Supplementary file 1 [file molecules-22-00467-s001.zip › Supplementary Materials/b17(HRMS).pdf]
